# Development of methodologies for the regioselective synthesis of four series of regioisomer isoxazoles from β-enamino diketones†

**DOI:** 10.1039/c7ra13343j

**Published:** 2018-01-25

**Authors:** Raí G. M. Silva, Michael J. V. da Silva, Andrey P. Jacomini, Sidnei Moura, Davi F. Back, Ernani A. Basso, Fernanda A. Rosa

**Affiliations:** Departamento de Química, Universidade Estadual de Maringá (UEM) 87030-900 Maringá PR Brazil farosa@uem.br; Laboratório de Produtos Naturais e Sintéticos, Instituto de Biotecnologia, Universidade de Caxias do Sul (UCS) 95070-560 Caxias do Sul RS Brazil; Departamento de Química, Universidade Federal de Santa Maria (UFSM) 97110-970 Santa Maria RS Brazil

## Abstract

Four methodologies are reported for the regioselective synthesis of four series of regioisomer isoxazoles from cyclocondensation of β-enamino diketones and hydroxylamine hydrochloride. Regiochemical control was achieved by varying reaction conditions and substrate structure. The mild reaction conditions used to access 4,5-disubstituted, 3,4-disubtituted, and 3,4,5-trisubstituted regioisomer isoxazoles, as well as the pharmacological and synthetic potential of the products, make these novel methodologies very powerful.

## Abstract 2

Efficient and regioselective synthesis of isoxazoles was demonstrated through cyclocondensation of β-enamino diketones with hydroxylamine: four of the six possible regioisomers were obtained.

## Graphical abstract

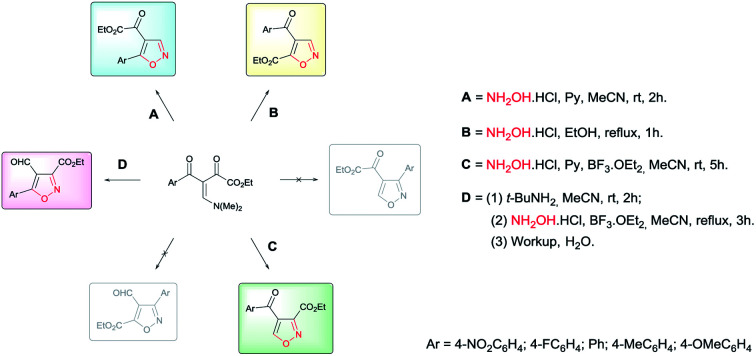

## Introduction

**Fig. 1:**
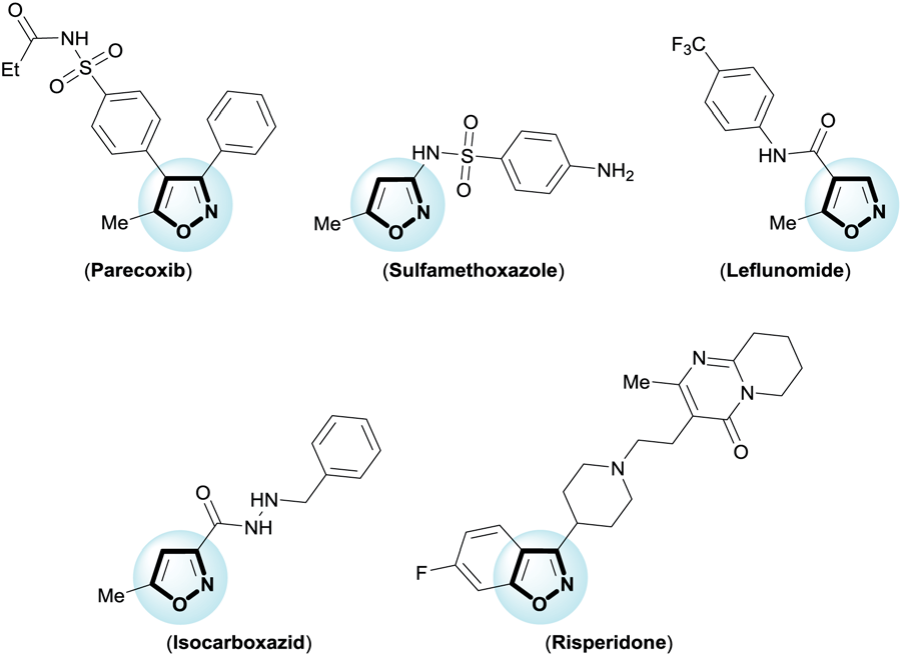
Examples of medicinal products with the isoxazole moiety.

The isoxazole is an important framework because it is the core structure of remarkable medicinal products.^1^ For example, parecoxib (anti-inflammatory); sulfamethoxazole (antibiotic); leflunomide (antirheumatic); isocarboxazid (antidepressant); and risperidone (antipsychotic) (Fig. 1). Moreover, isoxazoles are masked 1,3-dicarbonyl equivalents,^2^ which serve as precursors for natural product synthesis, as for example tetracycline antibiotics.^2*c*–*h*^ For this reason, many synthetic methods have been reported for the synthesis of functionalized isoxazoles,^3^ including ring construction with functionalized precursors by 1,3-dipolar cycloadditions^4^ or cyclocondensation^5^ reactions. One of the most popular, oldest, and most important methods for the synthesis of isoxazoles is cyclocondensation of 1,3-dicarbonyl with hydroxylamine (Claisen isoxazole synthesis).^5*a*–*i*^ However, this approach suffers from frequent formation of a regioisomeric mixture of isoxazoles with poor selectivity, harsh reaction conditions, and a limited reaction scope. Furthermore, obtaining 4-substituted isoxazoles using this approach has been challenging. However, development of new cyclocondensation reactions has received little attention. Despite these challenges, our research group was motivated to develop a new methodology for synthesis of functionalized isoxazoles from β-enamino diketone and hydroxylamine. The β-enamino diketones have been employed as precursors in functionalized heterocycles synthesis due to their excellent 1,3-dielectrophilic system, and in general they allow better control of regioselectivity.^6^

Over time, we have developed regioselective synthetic methodologies for the synthesis of multifunctionalized heterocycles from β-enamino diketones and different dinucleophiles.^6^ Variations in reaction conditions,^6*e*^ Lewis acid,^6*f*^ and β-enamino diketone structure^6*e*,*f*^ have resulted in regiochemical control of pyrazoles,^6*a*–*c*,*e*,*f*^ pyrazolo-pyridazinones,^6*b*,*c*^ pyrimidines,^6*d*^ and their derivatives. Thus, we believe that the enamino diketones are potential precursors for the regioselective synthesis of functionalized isoxazoles by cyclocondensation with hydroxylamine. To our knowledge, there is only a single report in the literature regarding isoxazole synthesis from enamino diketone and hydroxylamine, where the authors have used a symmetrical β-enamino diketone.^7^ Furthermore, a study of reactivity of the β-enamino diketone system with hydroxylamine has not yet been reported.

**Scheme 1:**
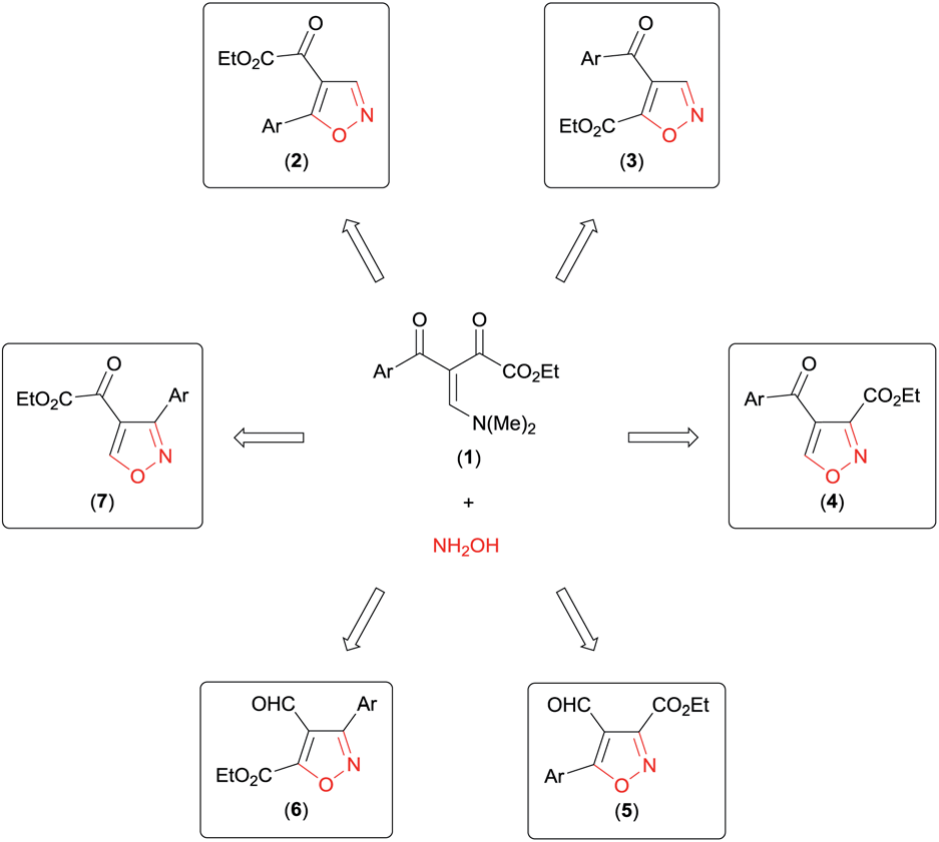
Possible regioisomer isoxazoles obtained by cyclocondensation of β-enamino diketone with hydroxylamine.

Analysing the reactive potential of the β-enamino diketone precursor in the cyclocondensation reaction with hydroxylamine, we observed that it would lead to formation of six regioisomer isoxazoles (Scheme 1). Thus, continuing our interest in this area, we report herein four methodologies to obtain functionalized 4,5-disubstituted isoxazoles, 3,4-disubstituted isoxazoles, and 3,4,5-trisubstituted isoxazoles by regiochemical control of the cyclocondensation reaction of β-enamino diketones with hydroxylamine.

## Results and discussion

We began this study using the β-enamino diketone 1a^8^ and hydroxylamine hydrochloride (NH_2_OH·HCl) as model substrates (Table 1). Gratifyingly, in EtOH at room temperature, these substrates performed well, leading to a regioisomeric mixture of the 4,5-disubstituted isoxazoles 2a and 3a with selectivity favouring 3a in good yield (Table 1, entry 1). The structure of 2a and 3a were established by NMR spectral data and unambiguously confirmed by X-ray crystallography^9*a*,*b*^ (see Fig. SI 1 and 2 for full details, ESI†). Encouraged by these results, other reaction conditions were investigated (Table 1). First, based on data recently reported by Souza *et al.*,^6*e*^ we examined MeCN and the mixture H_2_O/EtOH (1 : 1) as solvents in the reaction of 1a with NH_2_OH·HCl (Table 1, entries 2 and 3). The use of the aprotic polar solvent MeCN resulted in 2a as the main product in good yield (Table 1, entry 2).

**Table 1:** Optimization of reaction conditions of 1a with NH_2_OH·HCl to access 4,5-disubstituted isoxazoles regioisomers 2a and 3a regioselectively^a^

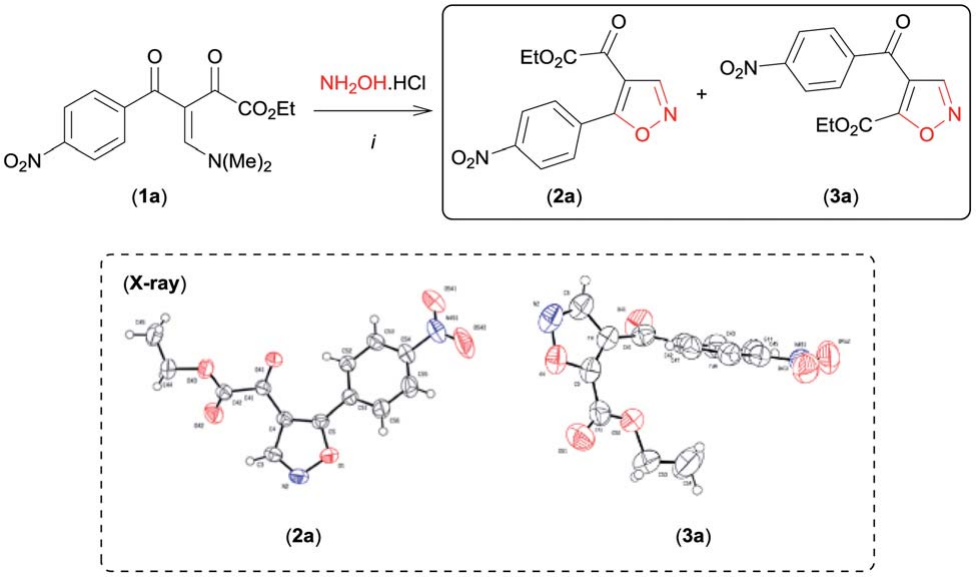						
Entry	*i*			Ratio^b^ (%)		Yield^c^ (%)
	Solvent	Base	Time (h)/Temp. (°C)	2a	3a	
1	EtOH	—	10/25	35	65	73
2	MeCN	—	16/25	65	35	81
3	EtOH/H_2_O	—	10/25	40	60	68
4	EtOH	Py	2/25	64	36	71
**5**	**MeCN**	**Py**	**2/25**	**76**	**24**	**87**
6	MeCN	DBU	2/25	—d		—
7	MeCN	K_2_CO_3_	2/25	—d		—
**8**	**EtOH**	—	**1/reflux**	**23**	**77**	**76**
9	MeCN	—	3/reflux	54	46	78
10	MeCN	Py	1/reflux	45	55	80
11	EtOH	Py	1/reflux	62	38	74

a Reaction conditions: 1a (0.5 mmol), NH_2_OH·HCl (0.6 mmol, 1.2 equiv.), base (0.6 mmol, 1.2 equiv.), solvent (4 mL).

b Calculated from the ^1^H-NMR spectrum of crude product.

c Isolated yield (regioisomeric mixture).

d 2a and 3a as intractable mixtures of several products.

On the other hand, the protic polar solvents mixture H_2_O/EtOH yielded 3a as the main product (Table 1, entry 3), but this solvent was found to be less regioselective than EtOH (Table 1, entry 1). Next, we examined the reaction mediated by bases at room temperature (Table 1, entries 4–7, 10 and 11). Interestingly, only pyridine was compatible with the reaction, favouring the regioselective formation of 2a in EtOH and mainly in MeCN (Table 1, entries 4 and 5). The reactions of other bases led to the formation of 2a and 3a as intractable mixtures of several products (Table 1, entries 6 and 7). Finally, by reacting 1a with NH_2_OH·HCl in EtOH at reflux, 3a was formed with higher regioselectivity (Table 1, entry 8) than at room temperature (Table 1, entry 1). In contrast, varying the reaction temperature in MeCN we discovered that increasing the temperature can jeopardize the regioselectivity of the reaction (Table 1, entry 9). In general, regioisomer 2a was favoured in MeCN with pyridine at room temperature (Table 1, entry 5), whereas 3a was preferentially formed in EtOH at reflux (Table 1, entry 8).

Having established the reaction conditions for synthesis of both regioisomeric 4,5-disubstituted isoxazoles 2a and 3a with moderate regioselectivity, we became interested in reversing the reactivity of the β-enamino diketone 1a toward hydroxylamine hydrochloride by varying the reaction conditions, so as to access 3,4-disubstituted isoxazoles regioselectively. To our surprise, when 1a was reacted with NH_2_OH·HCl in the presence of the Lewis acid carbonyl activator BF_3_ (BF_3_·OEt_2_) (0.5 equiv.) in MeCN at room temperature, the desired 3,4-disubstituted isoxazole 4a was formed as the main product (Table 2, entry 1). The structure of 4a was unambiguously established from its spectral and X-ray crystallographic data^9*c*^ (see Fig. SI 3 for full details, ESI†).

**Table 2:** Optimization of reaction conditions of 1a with NH_2_OH·HCl mediated by BF_3_·OEt_2_ to access 3,4-disubstituted isoxazole 4a regioselectively^a^

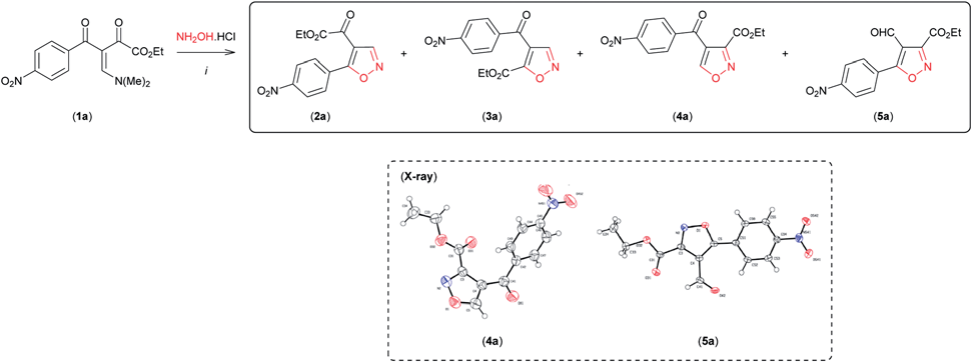								
Entry	*i*			Ratio^b^ (%)				Yield^c^ (%)
	Solvent	BF_3_·OEt_2_ (equiv.)	Time (h)	2a	3a	4a	5a	
1	MeCN	0.5	18	37	13	50	—	—
2	MeCN	1.0	20	22	8	70	—	—
3	MeCN	1.5	24	9	—	81	10	—
4	MeCN	2.0	24	—	—	90	10	79
**5** d	**MeCN**	**2.0**	**5**	—	—	**90**	**10**	**79**
6^d^	EtOH	2.0	2	64	36	—	—	—

a Reaction conditions: 1a (0.5 mmol), NH_2_OH·HCl (0.6 mmol, 1.2 equiv.), room temperature, solvent (4 mL).

b Calculated from the ^1^H-NMR spectrum of crude product.

c Isolated yield (regioisomeric mixture).

d Pyridine (1.4 equiv.).

By optimization of reaction conditions using this protocol, we observed that regioselectivity for the formation of isoxazole 4a was dependent on the amount of BF_3_ (Table 2, entries 1–4) and the solvent used (Table 2, entries 5 and 6). We obtained 4a with high regioselectivity (90%) in good yield (79%) employing 2 equivalents of BF_3_ in MeCN with pyridine at room temperature (Table 2, entry 5). The by-product mixed with 4a under these conditions (Table 2, entry 5) was isolated and characterized as 3,5-disubstituted 4-formyl-isoxazole 5a based on NMR spectral analysis and single crystal X-ray analysis^9*d*^ (see Fig. SI 4 for full details, ESI†).

**Scheme 2:**
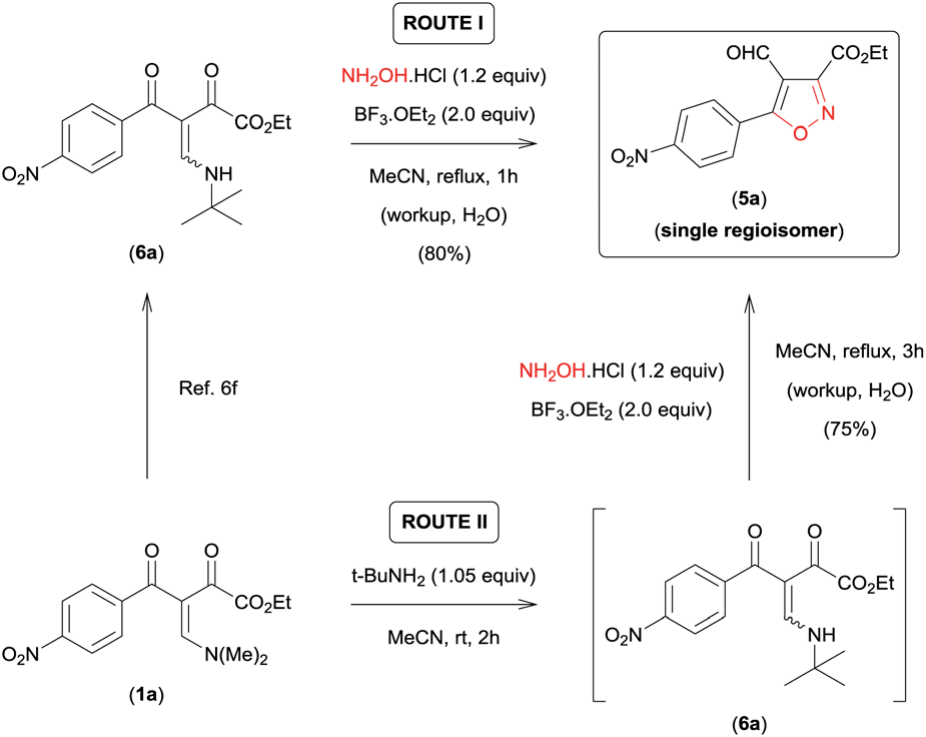
ROUTE I – Synthesis of 3,5-disubstituted 4-formyl-isoxazol 5a from β-enamino diketone 6a; ROUTE II – sequential one-pot procedure to obtain 5a from β-enamino diketone 1a.

On the basis of these observations, we have devoted our efforts to developing a methodology which allows to access 5a regioselectively. According to the data recently reported by da Silva *et al.*,^6*f*^ the presence of an aminoalkyl secondary group with high steric demand (i-PrNH– or *t*-BuNH–) bound to the β-carbon of the β-enamino diketone system in combination with the Lewis acid carbonyl activator BF_3_ provides conditions for the regiocontrolled reaction of β-enamino diketones with aryl hydrazines to give 3,5-disubstituted 4-formyl-*N*-arylpyrazoles with high regioselectivity. Thus, we tested the viability of this approach for the regioselective preparation of 3,5-disubstituted 4-formyl-isoxazole 5a. When we tested the reaction of β-enamino diketone 6a (ref. 6*f*) (1.0 equiv.), prepared from 1a (ref. 8) (Scheme 2), with NH_2_OH·HCl (1.2 equiv.) in MeCN and BF_3_·OEt_2_ (2.0 equiv.) at reflux for 1 h, the desired isoxazole 5a was obtained with 100% regioselectivity and in good yield (80%) (Scheme 2, ROUTE I).

Subsequently, the efficiency of this protocol was further improved by developing a sequential one-pot procedure to obtain isoxazole 5a directly from the β-enamino diketone 1a (Scheme 2, ROUTE II). The best results for this procedure were obtained by *in situ* generation of the β-enamino diketone precursor 6a from treatment of 1a with *tert*-butylamine (1.05 equiv.) in MeCN at room temperature for 2 h, followed by the addition of NH_2_OH·HCl (1.2 equiv.) and BF_3_·OEt_2_ (2.0 equiv.) under reflux of MeCN for 3 h (Scheme 2, ROUTE II). Through this procedure 5a was also obtained with 100% regioselectivity and a similar yield when prepared directly from the β-enamino diketone precursor 6a (Scheme 2, ROUTE I).

Having in hand the optimal reaction conditions to access 4,5-disubstituted (regioisomers 2a and 3a, Table 1, entries 5 and 8, respectively), 3,4-disubstituted (regioisomer 4a, Table 2, entry 5), and 3,5-disubstituted 4-formyl (regioisomer 5a, Scheme 2, ROUTE II) isoxazoles regioselectively from β-enamino diketone 1a and NH_2_OH·HCl, we examined the scope of this reaction under the conditions reported above, varying the electronic properties of the β-enamino diketone substrate 1. The results are summarized in Table 3.

**Table 3:** Substrate scope^a^

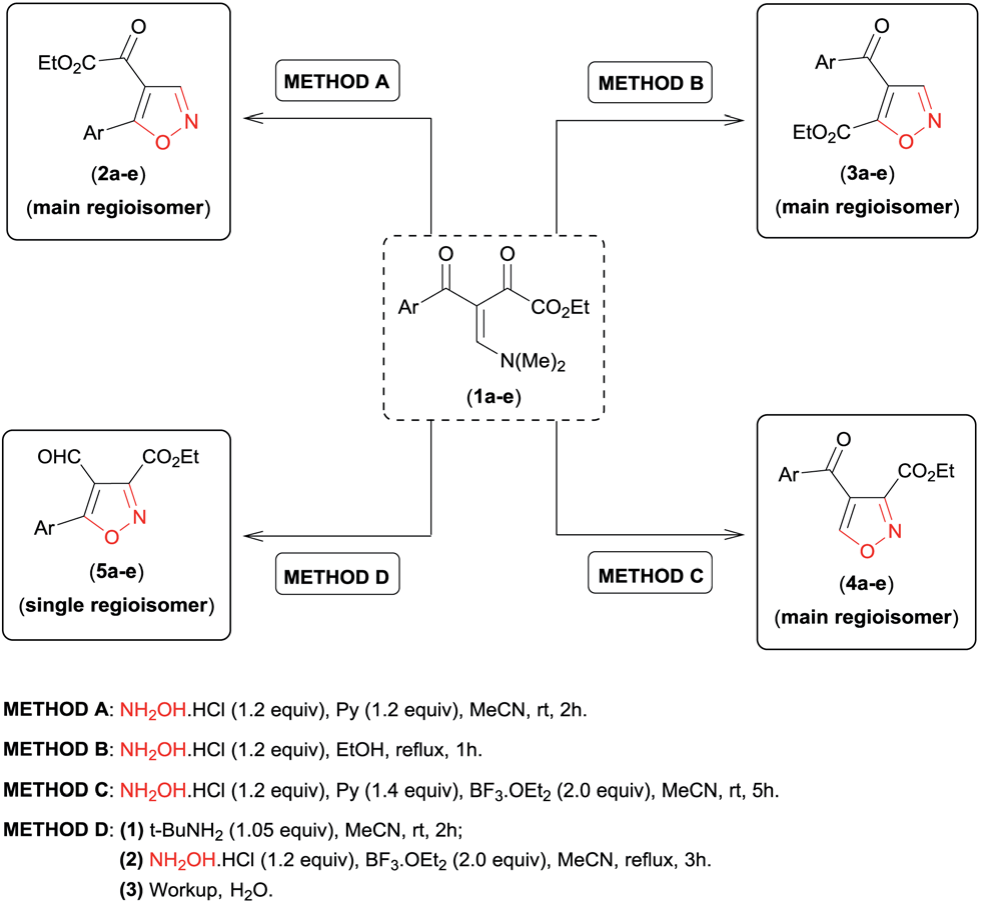				
Entry	Substrate (Ar)	Method	Ratio^b^ (%)	Yield^c^ (%)
1	1a (4-NO_2_C_6_H_4_)	A	2a(76), 3a(24)	87 (65)
2	1b (4-FC_6_H_4_)	A	2b(62), 3b(38)	88 (53)
3	1c (Ph)	A	2c(65), 3c(35)	89 (57)
4	1d (4-MeC_6_H_4_)	A	2d(60), 3d(40)	90 (52)
5	1e (4-OMeC_6_H_4_)	A	2e(58), 3e(42)	90 (50)
6	1a (4-NO_2_C_6_H_4_)	B	2a(23), 3a(77)	76 (58)
7	1b (4-FC_6_H_4_)	B	2b(20), 3b(80)	82 (65)
8	1c (Ph)	B	2c(20), 3c(80)	81 (64)
9	1d (4-MeC_6_H_4_)	B	2d(20), 3d(80)	81 (63)
10	1e (4-OMeC_6_H_4_)	B	2e(35), 3e(65)	83 (52)
11	1a (4-NO_2_C_6_H_4_)	C	4a(90), 5a(10)	79 (70)
12	1b (4-FC_6_H_4_)	C	4b(90), 5b(10)	81 (71)
13	1c (Ph)	C	4c(90), 5c(10)	72 (64)
14	1d (4-MeC_6_H_4_)	C	4d(90), 5d(10)	73 (65)
15	1e (4-OMeC_6_H_4_)	C	4e(90), 5e(10)	83 (74)
16	1a (4-NO_2_C_6_H_4_)	D	5a(100)	(75)
17	1b (4-FC_6_H_4_)	D	5b(100)	(65)
18	1c (Ph)	D	5c(100)	(62)
19	1d (4-MeC_6_H_4_)	D	5d(100)	(70)
20	1e (4-OMeC_6_H_4_)	D	5e(100)	(68)

a Reaction conditions: 1b–e (0.5 mmol), NH_2_OH·HCl (0.6 mmol, 1.2 equiv.), solvent (4 mL).

b Calculated from the ^1^H-NMR spectrum of crude product.

c Isolated yields (regioisomeric mixture); yields in parentheses are yields of the main regioisomer isolation by column chromatography.

Similar to the β-enamino diketone substrate 1a (Table 3, entries 1, 6, 11 and 16), all substrates examined (1b–e) were found to undergo the desired transformation to give the corresponding products in good to excellent yields (62–90%) (Table 3, entries 2–5, 7–10, 12–15, and 17–20). In general, the electronic nature of the Ar substituent on the β-enamino diketone 1a–e imposed a small effect on the regioselectivity of the reaction for methods A and B. For method A, the substrate bearing the stronger *p*-OMe (1e) electron-donating substituent provided low regioselectivity for the formation of the isoxazole regioisomer 2 (Table 3, entry 5), while the *p*-NO_2_ electron-withdrawing substituent provided high regioselectivity (Table 3, entry 1). For the other substituents (Table 3, entries 2–4), regioisomer 2 was obtained with moderate regioselectivity. On the other hand, for method B we did not see a clear correlation of the electronic nature of the substituents (Ar) with the regioselectivity of the formation of isoxazole 3 (Table 3, entries 6–10). In contrast, regardless of the different electronic properties of the Ar substituent on β-enamino diketone 1a–e, isoxazoles regioisomer 4 and 5 were always obtained with high regioselectivity (Table 3, entries 11–20).

**Fig. 2:**
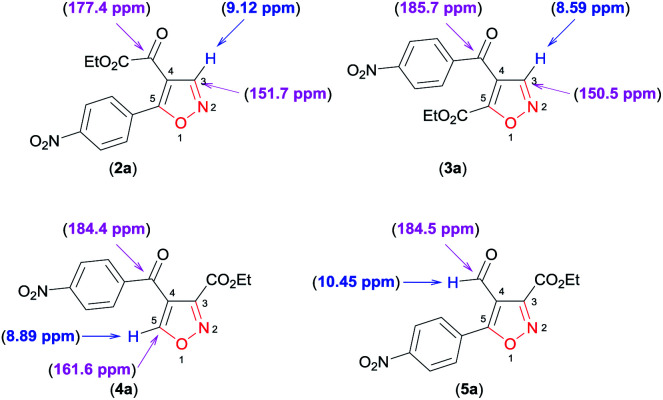
^1^H and ^13^C NMR chemical shifts of the regioisomers 2a, 3a, 4a, and 5a.

Finally, through detailed analysis of the NMR spectral data of the new isoxazoles reported here, we observed that difference in the chemical shifts of ^1^H and ^13^C allow a simple assignment of the different regioisomeric forms obtained. For example, we use isoxazoles 2a, 3a, 4a, and 5a as a model to show these differences (Fig. 2).

For the disubstituted isoxazoles 2a, 3a, and 4a, the hydrogen atom attached to the isoxazole nucleus (H3 for 2a and 3a, H5 for 4a – Fig. 2) have notable differences in the chemical shifts of ^1^H NMR spectrum. The H3 atom in 3a (8.59 ppm) is more shielded than the H5 atom in 4a (8.89 ppm) by a difference of approximately 0.30 ppm, whereas H5 (4a) is more shielded than the H3 atom in 2a (9.12 ppm) by about 0.23 ppm (Fig. 2). With regard to ^13^C NMR spectra of disubstituted isoxazoles, the major differences between the chemical shifts of the 4,5 (2a and 3a) and the 3,4 (4a)-disubstituted regioisomers are related to the carbon atoms C3 in 2a and 3a, and C5 in 4a (Fig. 2). This is because the C5 atom signal (compound 4a) is approximately 10 ppm more deshielded than the corresponding atom (C3) in 2a and 3a (Fig. 2). For the 4,5-disubstituted regioisomers 2a and 3a, the signal of the ketone carbonyl attached at the 4-position of the isoxazole ring shows considerable differences in the chemical shifts of the ^13^C NMR spectrum, because the ketone carbonyl signal in 2a is more shielded than the ketone carbonyl in 3a by about 8.3 ppm (Fig. 2). Unambiguously, 3,5-disubstituted 4-formyl isoxazole 5a could be identified by the characteristic chemical shifts of aldehyde hydrogen and carbon of the ^1^H and ^13^C NMR spectra (Fig. 2).

## Conclusions

In summary, we have developed four methodologies for the regioselective synthesis of polyfunctionalized isoxazoles by cyclocondensation of β-enamino diketones with hydroxylamine. The regiochemistry of the reaction has been controlled by: the solvent; use of pyridine; the Lewis acid carbonyl activator BF_3_; and the structure of the β-enamino diketone. These variations allowed access to four of the six possible regioisomer isoxazoles with good yields, which have different substitution patterns: 3,4-disubstituted, 4,5-disubstituted-, and 3,4,5-trisubstituted isoxazoles.

## Conflicts of interest

There are no conflicts to declare.

## Supplementary Material

RA-008-C7RA13343J-s001

RA-008-C7RA13343J-s002
