# Supplementary material for: Development of methodologies for the regioselective synthesis of four series of regioisomer isoxazoles from β-enamino diketones
Source: RSC Adv. 2018 Jan 25;8(9):4773–8. doi: 10.1039/c7ra13343j (PMC9077875; doi:10.1039/c7ra13343j)
Supplement: RA-008-C7RA13343J-s001 [file RA-008-C7RA13343J-s001.pdf]

Electronic Supplementary Material (ESI) for RSC Advances.
This journal is © The Royal Society of Chemistry 2018

Development of methodologies for the regioselective synthesis of four series of regioisomer isoxazoles from β-enamino diketones

Raí G. M. Silva,[a] Michael J. V. Silva,[a] Andrey P. Jacomini,[a] Sidnei Moura,[b] Davi F. Back,[c] Ernani A. Basso,[a] Fernanda A. Rosa*[a]

[a]Departamento de Química, Universidade Estadual de Maringá (UEM), 87030-900, Maringá PR, Brazil.

[b]Laboratório de Produtos Naturais e Sintéticos, Instituto de Biotecnologia, Universidade de Caxias do Sul (UCS), 295070-560, Caxias do Sul, RS, Brazil.

[c]Departamento de Química, Universidade Federal de Santa Maria (UFSM), 97110-970 - Santa Maria, RS, Brazil.

*E-mail: farosa@uem.br

SUPPORTING INFORMATION

Table of contents

1. General Information……………………………………………………………………………SI2

2. General synthetic procedure and spectra data……………………………………………SI2-9

2.1. *β*-enamino diketone substrates **1a-e**……………………………………………SI2

2.2. 4,5-disubstituted isoxazoles **2a-e** (**method A**)……………………………………SI2-4

2.3. 4,5-disubstituted isoxazoles **3a-e** (**method B**)……………………………………SI4-6

2.4. 3,4-disubstituted isoxazoles **4a-e** (**method C**)……………………………………SI6-8

2.5. 3,5-disubstituted 4-formyl- isoxazoles **5a-e** (**method D**)…………………………SI8-9

3. References……………………………………………………………………………………SI10

4. ORTEP Diagrams and X-ray crystallographic information of compounds 2,3,4,5a………SI11-14

5. ^{1}H and ^{13}C NMR spectra for new compounds (2,3,4,5a-e)……………………SI15-54

1. General Information:

Reagents were used as obtained from comercial suppliers without further purification. The solvents were dried and purified according to recommended procedures.[1] The reactions were monitored by thin-layer chromatography using Merck TLC silica gel plates and visualized with UV light. The column chromatography used was silica gel 60, with 230-400 mesh (Merck). All melting points were measured with MQAPF-307 Microquímica apparatus using benzoic acid as internal standard. ^{1}H and ^{13}C NMR, HSQC and HMBC experiments were run on VARIAN Mercury Plus apparatus operating at ^{1}H 300 MHz and ^{13}C 75 MHz, and Bruker avance III HD apparatus operating at ^{1}H 500 MHz and ^{13}C 125 MHz. Chemical shifts are reported in ppm using TMS as the internal standard for $CDCl_3$ and DMSO-d_6. ESI(+)-MS and tandem ESI(+)-MS/MS were acquired using a hybrid high-resolution and high accuracy microTof (Q-TOF) mass spectrometer (Bruker). For ESI(+)-MS, the energy for the collision induced dissociations (CDI) was optimised for each component. For data acquisition and processing, the Q-TOF-control data analysis software (Bruker Scientific) was used. Single Crystal X-ray diffraction studies: X-ray intensity data measurements of compounds **2a** (CCDC-1589617), **3a** (CCDC-1589618), **4a** (CCDC-1589619) and **5a** (CCDC-1589620) were collected with a Bruker APEX II CCD area-detector diffractometer and grafite-monochromatized Mo-Ka radiation. The structure was solved by direct methods using SHELXS.[2] Subsequent Fourier-difference map analyses yielded the positions of the non-hydrogen atoms. Refinements were carried out the SHELXS package.[2] All refinements were made by full matrix least squares on *F2* with anisotropic displacement parameters for all non-hydrogen atoms. Hydrogen atoms were included in the refinement in calculated positions but the atoms (of hydrogens) that are commenting performing special bond were located in the Fourier map. The ORTEP diagram were drawn with 50% probability displacement ellipsoids using ORTEP-3 for Windows.[3]

2. General synthetic procedure and spectra data:

2.1. *β*-enamino diketone substrates 1a-e:

The *β*-enamino diketone substrates **1a-e** were prepared according to the literature.[4]

2.2. 4,5-disubstituted isoxazoles 2a-e (method A):

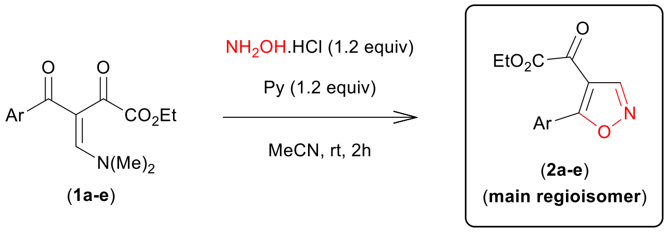

To a solution of *β*-enamino diketone **1** (**1a**: 160.1 mg; **1b**: 146.6 mg; **1c**: 137.6 mg; **1d**: 144.6 mg; **1e**: 152.6 mg, 0.5 mmol, 1.0 equiv) in acetonitrile (4 mL) were added hydroxylamine hydrochloride (41.7 mg, 0.6 mmol, 1.2 equiv) and pyridine (48 µL, 0.6 mmol, 1.2 equiv). The mixture was stirred at room temperature for 2 h. Then, the reaction mixture was concentrated under reduced pressure, poured into distilled H_2O (10 mL), extracted with dichloromethane (3 x 5 mL), washed with a solution of H_2O–HCl (10:1; 2 x 10 mL) and brine (2 × 10 mL). The organic layer was dried with anhydrous sodium sulfate and then the solvent was evaporated under reduced pressure to give the corresponding isoxazoles, which

were purified by chromatography column on a silica gel column (hexane/ethyl acetate, 20:80) to afford pure compounds **2a**-**e**.

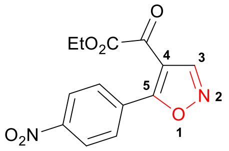

(**2a**)

4-(2-ethoxy-2-oxoacetyl)-5-(4-nitrophenyl) isoxazole (2a): Yellow solid; 65% yield (94.3 mg); mp 112.1-113.2°C; **^{1}H NMR** (300.06 MHz, $CDCl_3$) δ (ppm) 1.43 (t, 3H, J = 7.16 Hz, $OCH_2C\underline{H}_3$), 4.43 (q, 2H, J = 7.16 Hz, $OC\underline{H}_2CH_3$), 8.30 (d, 3H, J = 9.20 Hz, 4-$NO_2C_6H_4$), 8.38 (d, 2H, J = 9.20 Hz, 4-$NO_2C_6H_4$), 9.12 (s, 1H, H3); **^{13}C NMR** (75.46 MHz, $CDCl_3$) δ (ppm) 14.1 ($OCH_2\underline{C}H_3$), 63.5 ($O\underline{C}H_2CH_3$), 114.2 (C4), 123.9, 130.5, 131.4, 149.9 (4-$NO_2C_6H_4$), 152.0 (C3), 161.3 ($CO\underline{C}O_2Et$), 172.0 (C5), 176.9 ($\underline{C}OCO_2Et$); **HRMS** (ESI+): calcd for $C_{13}H_{11}N_2O_6^+$, $[M+H]^+$: 291.0612, found 291.0553.

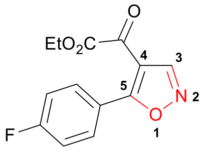

(**2b**)

4-(2-ethoxy-2-oxoacetyl)-5-(4-fluorophenyl) isoxazole (2b): Yellow solid; 53% yield (69.7 mg); mp 78.0-78.8°C; **^{1}H NMR** (500.13 MHz, $CDCl_3$) δ (ppm) 1.40 (t, 3H, J = 7.15 Hz, $OCH_2C\underline{H}_3$), 4.39 (q, 2H, J = 7.15 Hz, $OC\underline{H}_2CH_3$), 7.22 (dd, 2H, J = 9.03 Hz, 4-FC_6H_4), 8.16 (dd, 2H, J = 9.09, $J_{F\text{-}H}$ = 5.25 Hz, 4-FC_6H_4), 9.02 (s, 1H, H3); **^{13}C NMR** (125.77 MHz, $CDCl_3$) δ (ppm) 14.1 ($OCH_2\underline{C}H_3$), 63.2 ($O\underline{C}H_2CH_3$), 112.6 (C4), 116.2 (d, $^2J_{C\text{-}F}$ = 22.32 Hz, 4-FC_6H_4), 122.2 (d, $^4J_{C\text{-}F}$ = 3.25 Hz, 4-FC_6H_4), 131.9 (d, $^3J_{C\text{-}F}$ = 8.95 Hz, 4-FC_6H_4), 151.9 (C3), 161.8 ($CO\underline{C}O_2Et$), 165.3 (d, $^1J_{C\text{-}F}$ = 255.29 Hz, 4-FC_6H_4), 173.6 (C5), 177.1 ($\underline{C}OCO_2Et$); **HRMS** (ESI+): calcd for $C_{13}H_{11}FNO_4^+$, $[M+H]^+$: 264.0667, found 264.0670.

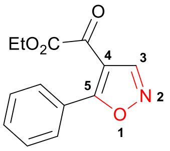

(**2c**)

4-(2-ethoxy-2-oxoacetyl)-5-phenyl isoxazole (2c): Light yellow viscous liquid; 57% yield (69.9 mg); **^{1}H NMR** (300.06 MHz, $CDCl_3$) δ (ppm) 1.35 (t, 3H, J = 7.15 Hz , $OCH_2C\underline{H}_3$), 4.34 (q, 2H, J = 7.15 Hz, $OC\underline{H}_2CH_3$), 7.50-7.63 (m, 3H, Ph), 8.03-8.07 (m, 2H, Ph), 9,00 (s, 1H, H3); **^{13}C NMR** (75.46 MHz, $CDCl_3$) δ (ppm) 14.0 ($OCH_2\underline{C}H_3$), 63.1 ($O\underline{C}H_2CH_3$), 112.8 (C4), 125.9, 128.8, 129.3, 132.6 (Ph), 151.7 (C3), 161.8 ($CO\underline{C}O_2Et$), 174.6 (C5), 177.3 ($\underline{C}OCO_2Et$); **HRMS** (ESI+): calcd for $C_{13}H_{12}NO_4^+$, $[M+H]^+$: 246.0761, found 246.0698.

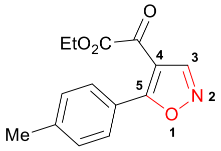

(**2d**)

4-(2-ethoxy-2-oxoacetyl)-5-(4-methylphenyl) isoxazole (2d): Light yellow solid; 52% yield (67.4 mg); mp 70.2-70.6 °C; ^{1}**H NMR** (300.06 MHz, $CDCl_3$) δ (ppm) 1.36 (t, 3H, *J* = 7.15 Hz, $OCH_2C\underline{H}_3$), 2.44 (s, 3H, 4-$C\underline{H}_3C_6H_4$), 4.35 (q, 2H, *J* = 7.14 Hz, $OC\underline{H}_2CH_3$), 7.33 (d, 2H, *J* = 8.04 Hz, 4-$CH_3C_6\underline{H}_4$), 7.98 (d, 2H, *J* = 8.29 Hz, 4-$CH_3C_6\underline{H}_4$), 8.97 (s, 1H, H3); ^{13}**C NMR** (75.46 MHz, $CDCl_3$) δ (ppm) 14.0 ($OCH_2\underline{C}H_3$), 21.8 (4-$\underline{C}H_3C_6H_4$), 63.0 ($O\underline{C}H_2CH_3$), 112.4 (C4), 123.2, 129.2, 129.5, 143.6 (4-$CH_3\underline{C}_6H_4$), 151.8 (C3), 161.9 ($CO\underline{C}O_2Et$), 174.8 (C5), 177.3 ($\underline{C}OCO_2Et$); **HRMS** (ESI+): calcd for $C_{14}H_{14}NO_4^+$, $[M+H]^+$: 260.0917, found 260.0849.

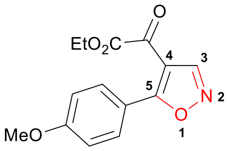

(**2e**)

4-(2-ethoxy-2-oxoacetyl)-5-(4-methoxyphenyl) isoxazole (2e): Yellow solid; 50% yield (68.8 mg); mp 66.7-67.6 °C; ^{1}**H NMR** (500.13 MHz, $CDCl_3$) δ (ppm) 1.39 (t, 3H, *J* = 7.15 Hz, $OCH_2C\underline{H}_3$), 3.90 (s, 3H, 4-$OC\underline{H}_3C_6H_4$), 4.38 (q, 2H, *J* = 7.17 Hz, $OC\underline{H}_2CH_3$), 7.03 (d, 2H, *J* = 8.95 Hz, 4-$OCH_3C_6\underline{H}_4$), 8.16 (d, 2H, *J* = 8.99 Hz, 4-$OCH_3C_6\underline{H}_4$), 8.97 (s, 1H, H3); ^{13}**C NMR** (125.77 MHz, $CDCl_3$) δ (ppm) 14.1 ($OCH_2\underline{C}H_3$), 55.6 (4-$O\underline{C}H_3C_6H_4$), 63.0 ($O\underline{C}H_2CH_3$), 111.7 (C4), 114.2, 118.4, 131.3 (4-$OCH_3\underline{C}_6H_4$), 151.9 (C3), 162.1 ($CO\underline{C}O_2Et$), 163.2 (4-$OCH_3\underline{C}_6H_4$), 174.4 (C5), 177.1 ($\underline{C}OCO_2Et$); **HRMS** (ESI+): calcd for $C_{14}H_{14}NO_5^+$, $[M+H]^+$: 276.0866, found 276.0876.

2.3. 4,5-disubstituted isoxazoles 3a-e (method B):

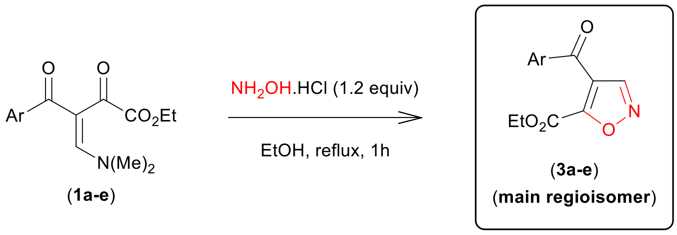

To a solution of *β*-enamino diketone **1** (**1a**: 160.1 mg; **1b**: 146.6 mg; **1c**: 137.6 mg; **1d**: 144.6 mg; **1e**: 152.6 mg, 0.5 mmol, 1.0 equiv) in ethanol (4 mL) was added hydroxylamine hydrochloride (41.7 mg, 0.6 mmol, 1.2 equiv), and stirred under reflux for 1 h. Then, the reaction mixture was concentrated under reduced pressure, poured into distilled H_2O (10 mL), extracted with dichloromethane (3 x 5 mL) and washed with a solution of brine (2 × 10 mL). The organic layer was dried with anhydrous sodium sulfate and then the solvent was evaporated under reduced pressure to give the corresponding isoxazoles, which were purified by chromatography column on a silica gel column (hexane/ethyl acetate, 20:80) to afford pure compounds **3a**-**e**.

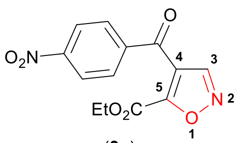

(**3a**)

5-(ethoxycarbonyl)-4-(4-nitrobenzoyl) isoxazole (3a): Yellow solid; 58% yield (84.1 mg); mp 118.3-193.2 °C; **^{1}H NMR** (300.06 MHz, $CDCl_3$) δ (ppm) 1.17 (t, 3H, J = 7.15 Hz, $OCH_2C\underline{H}_3$), 4.25 (q, 2H, J = 7.15 Hz, $OC\underline{H}_2CH_3$), 7.99 (d, 2H, J = 9.02 Hz, 4-$NO_2C_6H_4$), 8.35 (d, 2H, J = 9.01 Hz, 4-$NO_2C_6H_4$), 8.60 (s, 1H, H3); **^{13}C NMR** (75.46 MHz, $CDCl_3$) δ (ppm) 13.8 ($OCH_2\underline{C}H_3$), 63.3 ($O\underline{C}H_2CH_3$), 121.6 (C4), 124.1, 130.3, 141.5 (4-$NO_2C_6H_4$), 150.5 (C3), 150.9 (4-$NO_2C_6H_4$), 155.7 ($\underline{C}O_2Et$), 158.6 (C5), 185.7 ($\underline{C}O$).

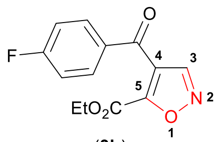

(**3b**)

5-(ethoxycarbonyl)-4-(4-fluorobenzoyl) isoxazole (3b): Yellow solid; 65% yield (85.5 mg); mp 108.5-109.0 °C; **^{1}H NMR** (500.13 MHz, $CDCl_3$) δ (ppm) 1.13 (t, 3H, J = 7.15 Hz, $OCH_2C\underline{H}_3$), 4.25 (q, 2H, J = 7.15 Hz, $OC\underline{H}_2CH_3$), 7.18 (dd, 2H, J = 8.89, $J_{F\text{-}H}$ = 8.34 Hz, 4-FC_6H_4), 7.86 (dd, 2H, J = 8.99, $J_{F\text{-}H}$ = 5.30 Hz, 4-FC_6H_4), 8.52 (s, 1H, H3); **^{13}C NMR** (125.77 MHz, $CDCl_3$) δ (ppm) 13.7 ($OCH_2\underline{C}H_3$), 63.0 ($O\underline{C}H_2CH_3$), 116.2 (d, $^2J_{C\text{-}F}$ = 22.10 Hz, 4-FC_6H_4), 122.0 (C4), 132.2 (d, $^3J_{C\text{-}F}$ = 9.61 Hz, 4-FC_6H_4), 133.4 (d, $^4J_{C\text{-}F}$ = 2.94 Hz, 4-FC_6H_4), 150.4 (C3), 155.9 ($\underline{C}O_2Et$), 158.0 (C5), 166.5 (d, $^1J_{C\text{-}F}$ = 257.42 Hz, 4-FC_6H_4), 185.5 ($\underline{C}O$); **HRMS** (ESI+): calcd for $C_{13}H_{11}FNO_4^+$, $[M+H]^+$: 264.0667, found 264.0676.

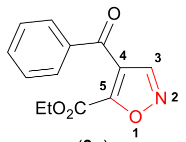

(**3c**)

4-benzoyl-5-(ethoxycarbonyl) isoxazole (3c): White solid; 64% yield (78.4 mg); mp 83.8-85.0 °C; **^{1}H NMR** (300.06 MHz, $CDCl_3$) δ (ppm) 1.06 (t, 3H, J = 7.15 Hz, $OCH_2C\underline{H}_3$), 4.20 (q, 2H, J = 7.16 Hz, $OC\underline{H}_2CH_3$), 7.47-7.54 (m, 2H, Ph), 7.62-7.68 (m, 1H, Ph), 7.81-7.84 (m, 2H, Ph), 8.54 (s, 1H, H3); **^{13}C NMR** (75.46 MHz, $CDCl_3$) δ (ppm) 13.6 ($OCH_2\underline{C}H_3$), 62.9 ($O\underline{C}H_2CH_3$), 122.1 (C4), 128.9, 129.5, 134.3, 137.0 (Ph), 150.6 (C3), 156.0 ($\underline{C}O_2Et$), 158.2 (C5), 187.1 ($\underline{C}O$); **HRMS** (ESI+): calcd for $C_{13}H_{12}NO_4^+$, $[M+H]^+$: 246.0761, found 246.0708.

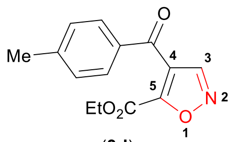

(**3d**)

5-(ethoxycarbonyl)-4-(4-methylbenzoyl) isoxazole (3d): Light yellow solid; 63% yield (81.6 mg); mp 77.6-78.3 °C; **^{1}H NMR** (300.06 MHz, $CDCl_3$) δ (ppm) 1.09 (t, 3H, J = 7.14 Hz, $OCH_2C\underline{H}_3$), 2.44 (s, 3H, 4-$C\underline{H}_3C_6H_4$), 4.22 (q, 2H, J = 7.14 Hz, $OC\underline{H}_2CH_3$), 7.28-7.31 (m, 2H, 4-$CH_3C_6\underline{H}_4$), 7.72 (d, 2H, J = 8.24 Hz, 4-$CH_3C_6\underline{H}_4$), 8.51 (s, 1H, H3); **^{13}C NMR** (75.46 MHz,

$CDCl_3$) δ (ppm) 13.6 ($OCH_2\underline{C}H_3$), 21.9 ($4\text{-}\underline{C}H_3C_6H_4$), 62.8 ($O\underline{C}H_2CH_3$), 122.4 (C4), 129.6, 129.6, 134.5, 145.6 ($4\text{-}CH_3\underline{C_6}H_4$), 150.5 (C3), 156.0 ($\underline{C}O_2Et$), 157.9 (C5), 186.6 ($\underline{C}O$); **HRMS** (ESI+): calcd for $C_{14}H_{14}NO_4^+$, $[M+H]^+$: 260.0917, found 260.0863.

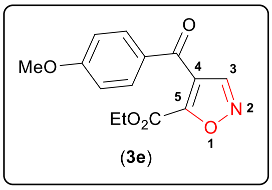

5-(ethoxycarbonyl)-4-(4-methoxybenzoyl) isoxazole (3e): White solid; 52% yield (71.5 mg); mp 84.0-84.7 °C; **^{1}H NMR** (500.13 MHz, $CDCl_3$) δ (ppm) 1.12 (t, 3H, *J* = 7.14 Hz, $OCH_2C\underline{H_3}$), 3.90 (s, 3H, $4\text{-}OC\underline{H_3}C_6H_4$), 4.25 (q, 2H, *J* = 7.16 Hz, $OC\underline{H_2}CH_3$), 6.96 (d, 2H, *J* = 9.00 Hz, $4\text{-}OCH_3C_6\underline{H_4}$), 7.81 (d, 2H, *J* = 8.92 Hz, $4\text{-}OCH_3C_6\underline{H_4}$), 8.49 (s, 1H, H3); **^{13}C NMR** (125.77 MHz, $CDCl_3$) δ (ppm) 13.7 ($OCH_2\underline{C}H_3$), 55.8 ($4\text{-}\underline{C}H_3C_6H_4$), 62.8 ($O\underline{C}H_2CH_3$), 114.2 ($4\text{-}OCH_3\underline{C_6}H_4$), 122.6 (C4), 129.9, 132.0 ($4\text{-}OCH_3\underline{C_6}H_4$), 150.5 (C3), 156.1 ($\underline{C}O_2Et$), 157.6 (C5), 164.7 ($4\text{-}OCH_3\underline{C_6}H_4$), 185.5 ($\underline{C}O$); **HRMS** (ESI+): calcd for $C_{14}H_{14}NO_5^+$, $[M+H]^+$: 276.0866, found 276.0888.

2.4. 3,4-disubstituted isoxazoles 4a-e (method C):

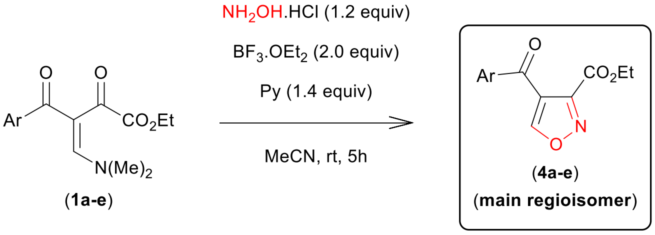

To a solution of *β*-enamino diketone **1** (**1a**: 160.1 mg; **1b**: 146.6 mg; **1c**: 137.6 mg; **1d**: 144.6 mg; **1e**: 152.6 mg, 0.5 mmol, 1.0 equiv) in acetonitrile (4 mL) were added hydroxylamine hydrochloride (41.7 mg, 0.6 mmol, 1.2 equiv), boron trifluoride diethyl etherate solution 46.5% (270 µL, 1.0 mmol, 2.0 equiv) and pyridine (56 µL, 0,7 mmol, 1,4 equiv). The mixture was stirred at room temperature for 5 h. Then, the reaction mixture was concentrated under reduced pressure, poured into distilled H_2O (10 mL), extracted with dichloromethane (3 x 5 mL), washed with a solution of H_2O–HCl (10:1; 2 x 10 mL) and brine (2 × 10 mL). The organic layer was dried with anhydrous sodium sulfate and then the solvent was evaporated under reduced pressure to give the corresponding isoxazoles, which were purified by chromatography column on a silica gel column (hexane/ethyl acetate, 20:80) to afford pure compounds **4a**-**e**.

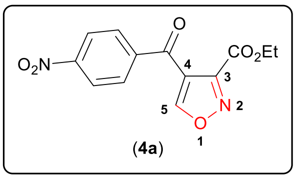

3-(ethoxycarbonyl)-4-(4-nitrobenzoyl) isoxazole (4a): Light yellow solid; 70% yield (101.5 mg); mp 90.4-92.4 °C; **^{1}H NMR** (500.13 MHz, $CDCl_3$) δ (ppm) 1.30 (t, 3H, *J* = 7.15 Hz, $OCH_2C\underline{H_3}$), 4.35 (q, 2H, *J* = 7.14 Hz, $OC\underline{H_2}CH_3$), 8.01 (d, 2H, *J* = 8.96 Hz, $4\text{-}NO_2C_6H_4$), 8.36 (d, 2H, *J* = 8.95 Hz, $4\text{-}NO_2C_6H_4$), 8.89 (s, 1H, H5); **^{13}C NMR** (125.77 MHz, $CDCl_3$) δ (ppm) 13.9

($OCH_2\underline{C}H_3$), 63.2 ($O\underline{C}H_2CH_3$), 120.1 (C4), 124.2, 130.1, 141.8, 150.8 (4-$NO_2C_6H_4$), 154.4 (C3), 158.9 ($\underline{C}O_2Et$), 161.7 (C5), 184.5 ($\underline{C}O$); **HRMS** (ESI+): calcd for $C_{13}H_{11}N_2O_6^+$, $[M+H]^+$: 291.0612, found 291.0544.

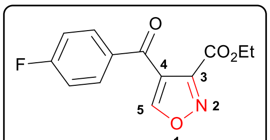

(4b)

3-(ethoxycarbonyl)-4-(4-fluorobenzoyl) isoxazole (4b): Yellow viscous liquid; 71% yield (93.4 mg); **^{1}H NMR** (500.13 MHz, $CDCl_3$) δ (ppm) 1.27 (t, 3H, J = 7.15 Hz, $OCH_2C\underline{H}_3$), 4.35 (q, 2H, J = 7.14 Hz, $OC\underline{H}_2CH_3$), 7.19 (t, 2H, J = 8.57 Hz, 4-FC_6H_4), 7.88 (dd, 2H, J = 8.87, $J_{F\text{-}H}$ = 5.29 Hz, 4-FC_6H_4), 8.79 (s, 1H, H5); **^{13}C NMR** (125.77 MHz, $CDCl_3$) δ (ppm) 13.9 ($OCH_2\underline{C}H_3$), 63.0 ($O\underline{C}H_2CH_3$), 116.2 (d, $^2J_{C\text{-}F}$ = 22.02 Hz, 4-FC_6H_4), 120.3 (C4), 132.0 (d, $^3J_{C\text{-}F}$ = 9.46 Hz, 4-FC_6H_4), 133.7 (d, $^4J_{C\text{-}F}$ = 3.00 Hz, 4-FC_6H_4), 154.6 (C3), 159.1 ($\underline{C}O_2Et$), 160.8 (C5), 166.4 (d, $^1J_{C\text{-}F}$ = 256.71 Hz, 4-FC_6H_4), 184.4 ($\underline{C}O$); **HRMS** (ESI+): calcd for $C_{13}H_{11}FNO_4^+$, $[M+H]^+$: 264.0667, found 264.0679.

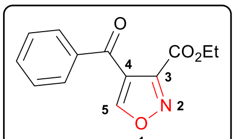

(4c)

4-benzoyl-3-(ethoxycarbonyl) isoxazole (4c): Yellow viscous liquid; 64% yield (78.4 mg); **^{1}H NMR** (300.06 MHz, $CDCl_3$) δ (ppm) 1.24 (t, 3H, J = 7.15 Hz , $OCH_2C\underline{H}_3$), 4.32 (q, 2H, J = 7.14 Hz, $OC\underline{H}_2CH_3$), 7.48-7.55 (m, 2H, Ph), 7.62-7.68 (m, 1H, Ph), 7.83-7.87 (m, 2H, Ph), 8.80 (s, 1H, H5); **^{13}C NMR** (75.46 MHz, $CDCl_3$) δ (ppm) 13.8 ($OCH_2\underline{C}H_3$), 62.9 ($O\underline{C}H_2CH_3$), 120.5 (C4), 129.0, 129.3, 134.1, 137.3 (Ph), 154.8 (C3), 159.2 ($\underline{C}O_2Et$), 161.1 (C5), 185.9 ($\underline{C}O$); **HRMS** (ESI+): calcd for $C_{13}H_{12}NO_4^+$, $[M+H]^+$: 246.0761, found 246.0695.

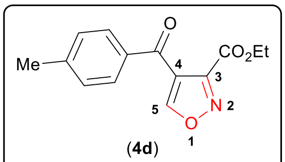

(4d)

3-(ethoxycarbonyl)-4-(4-methylbenzoyl) isoxazole (4d): White viscous liquid; 65% yield (84.2 mg); **^{1}H NMR** (300.06 MHz, $CDCl_3$) δ (ppm) 1.25 (t, 3H, J = 7.15 Hz, $OCH_2C\underline{H}_3$), 2.45 (s, 3H, 4-$C\underline{H}_3C_6H_4$), 4.33 (q, 2H, J = 7.14 Hz, $OC\underline{H}_2CH_3$), 7.29-7.32 (m, 2H, 4-$CH_3C_6\underline{H}_4$), 7.75 (d, 2H, J = 8.20 Hz, 4-$CH_3C_6\underline{H}_4$), 8.77 (s, 1H, H5); **^{13}C NMR** (75.46 MHz, $CDCl_3$) δ (ppm) 13.8 ($OCH_2\underline{C}H_3$), 21.8 (4-$\underline{C}H_3C_6H_4$), 62.8 ($O\underline{C}H_2CH_3$), 120.6 (C4), 129.4, 129.7, 134.8, 145.2 (4-$CH_3\underline{C}_6H_4$), 154.8 (C3), 159.2 ($\underline{C}O_2Et$), 160.8 (C5), 185.5 ($\underline{C}O$); **HRMS** (ESI+): calcd for $C_{14}H_{14}NO_4^+$, $[M+H]^+$: 260.0917, found 260.0843.

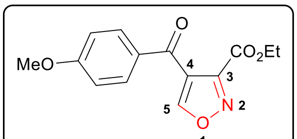

(4e)

3-(ethoxycarbonyl)-4-(4-methoxybenzoyl) isoxazole (4e): White viscous liquid; 74% yield (101.8 mg); **^{1}H NMR** (500.13 MHz, $CDCl_3$) δ (ppm) 1.26 (t, 3H, J = 7.15 Hz, $OCH_2C\underline{H}_3$), 3.90 (s, 3H, 4-$OC\underline{H}_3C_6H_4$), 4.34 (q, 2H, J = 7.18 Hz, $OC\underline{H}_2CH_3$), 6.98

(d, 2H, *J* = 8.88 Hz, 4-OCH3C6H4), 7.84 (d, 2H, *J* = 8.88 Hz, 4-OCH3C6H4), 8.75 (s, 1H, H5); **^{13}C NMR** (125.77 MHz, $CDCl_3$) δ (ppm) 13.9 (OCH_2CH_3), 55.7 (4-$OCH_3C_6H_4$), 62.8 (OCH_2CH_3), 114.2 (4-$OCH_3C_6H_4$), 120.6 (C4), 130.2, 131.7 (4-$OCH_3C_6H_4$), 154.7 (C3), 159.2 (CO_2Et), 160.4 (C5), 164.4 (4-$OCH_3C_6H_4$), 184.4 (CO); **HRMS** (ESI+): calcd for $C_{14}H_{14}NO_5^+$, $[M+H]^+$: 276.0866, found 276.0880.

2.5. 3,5-disubstituted 4-formyl isoxazoles 5a-e (method D):

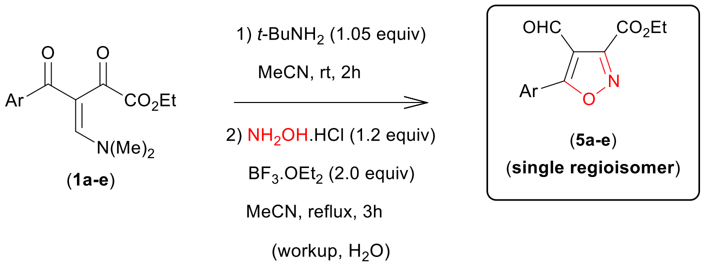

A mixture of compound **1** (**1a**: 160.1 mg; **1b**: 146.6 mg; **1c**: 137.6 mg; **1d**: 144.6 mg; **1e**: 152.6 mg, 0.5 mmol, 1.0 equiv) and *tert*-butylamine (38.5 mg, 0.525 mmol, 1.05 equiv) in acetonitrile (4 mL) was stirred at room temperature for 2 h. Next, hydroxylamine hydrochloride (41.7 mg, 0.6 mmol, 1.2 equiv) and boron trifluoride diethyl etherate solution 46.5% (270 µL, 1.0 mmol, 2.0 equiv) were added to mixture and stirred under reflux for 3 h. Then, the reaction mixture was concentrated under reduced pressure, poured into distilled H_2O (10 mL), extracted with dichloromethane (3 x 5 mL), washed with a solution of H_2O–HCl (10:1; 2 x 10 mL) and brine (2 × 10 mL). The organic layer was dried with anhydrous sodium sulfate and then the solvent was evaporated under reduced pressure to give the corresponding isoxazoles, which were purified by chromatography column on a silica gel column (hexane/ethyl acetate, 20:80) to afford pure compounds **5a**-**e**.

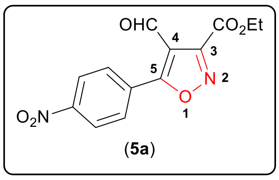

3-(ethoxycarbonyl)-4-formyl-5-(4-nitrophenyl) isoxazole (5a): Light orange solid; 75% yield (108.8 mg); mp 123.0-124.2 °C; **^{1}H NMR** (500.13 MHz, $CDCl_3$) δ (ppm) 1.50 (t, 3H, *J* = 7.16 Hz , OCH_2CH_3), 4.57 (q, 2H, *J* = 7.17 Hz, OCH_2CH_3), 8.40 (d, 2H, *J* = 9.19 Hz, 4-$NO_2C_6H_4$), 8.43 (d, 2H, *J* = 9.24 Hz, 4-$NO_2C_6H_4$), 10.47 (s, 1H, CHO); **^{13}C NMR** (125.77 MHz, $CDCl_3$) δ (ppm) 14.3 (OCH_2CH_3), 63.5 (OCH_2CH_3), 116.8 (C4), 124.1, 130.3, 130.9, 150.1 (4-$NO_2C_6H_4$), 156.0 (C3), 159.4 (CO_2Et), 170.9 (C5), 184.5 (CHO); **HRMS** (ESI+): calcd for $C_{13}H_{11}N_2O_6^+$, $[M+H]^+$: 291.0612, found 291.0540.

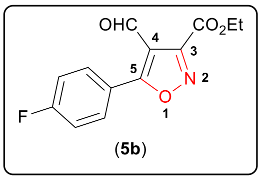

3-(ethoxycarbonyl)-5-(4-fluorophenyl)-4-formyl isoxazole (5b): Yellow solid; 65% yield (85.5 mg); mp 72.9-74.0°C; **^{1}H NMR** (500.13 MHz, $CDCl_3$) δ (ppm) 1.48 (t, 3H, *J* = 7.17 Hz, OCH_2CH_3), 4.55 (q, 2H, *J* = 7.17 Hz, OCH_2CH_3), 7.23-7.26 (m,

2H, 4-FC_6H_4), 8.27 (dd, 2H, *J* = 9.06, $J_{F\text{-}H}$ = 5.25 Hz, 4-FC_6H_4), 10.42 (s, 1H, CHO); **^{13}C NMR** (125.77 MHz, $CDCl_3$) δ (ppm) 14.2 (OCH_2CH_3), 63.2 (OCH_2CH_3), 115.1 (C4), 116.3 (d, $^2J_{C\text{-}F}$ = 21.88 Hz, 4-FC_6H_4), 121.8 (d, $^4J_{C\text{-}F}$ = 3.23 Hz, 4-FC_6H_4), 131.7 (d, $^3J_{C\text{-}F}$ = 9.09 Hz, 4-FC_6H_4), 155.9 (C3), 159.6 (CO_2Et), 166.5 (d, $^1J_{C\text{-}F}$ = 255.69 Hz, 4-FC_6H_4), 172.6 (C5), 184.5 (CHO); **HRMS** (ESI+): calcd for $C_{13}H_{11}FNO_4^+$, $[M+H]^+$: 264.0667, found 264.0684.

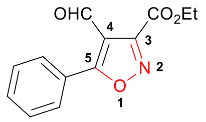

(5c)

3-(ethoxycarbonyl)-4-formyl-5-phenyl isoxazole (5c): Yellow viscous liquid; 62% yield (76.0 mg); **^{1}H NMR** (300.06 MHz, $CDCl_3$) δ (ppm) 1.48 (t, 3H, *J* = 7.14 Hz, OCH_2CH_3), 4.55 (q, 2H, *J* = 7.15 Hz, OCH_2CH_3), 7.53-7.65 (m, 3H, Ph), 8.14-8.18 (m, 2H, Ph), 10.40 (s, 1H, CHO); **^{13}C NMR** (75.46 MHz, $CDCl_3$) δ (ppm) 14.2 (OCH_2CH_3), 63.1 (OCH_2CH_3), 115.3 (C4), 125.5, 129.0, 129.0, 132.9 (Ph), 155.7 (C3), 159.7 (CO_2Et), 173.8 (C5), 184.3 (CHO); **HRMS** (ESI+): calcd for $C_{13}H_{12}NO_4^+$, $[M+H]^+$: 246.0761, found 246.0695.

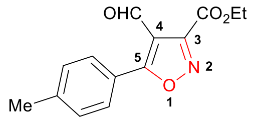

(5d)

3-(ethoxycarbonyl)-4-formyl-5-(4-methylphenyl) isoxazole (5d): White solid; 70% yield (90.7 mg); mp 57.2-57.7 °C; **^{1}H NMR** (300.06 MHz, $CDCl_3$) δ (ppm) 1.48 (t, 3H, *J* = 7.14 Hz, OCH_2CH_3), 2.46 (s, 3H, 4-$CH_3C_6H_4$), 4.54 (q, 2H, *J* = 7.15 Hz, OCH_2CH_3), 7.34-7.37 (m, 2H, 4-$CH_3C_6H_4$), 8.07 (d, 2H, *J* = 8.30 Hz, 4-$CH_3C_6H_4$), 10.39 (s, 1H, CHO); **^{13}C NMR** (75.46 MHz, $CDCl_3$) δ (ppm) 14.2 (OCH_2CH_3), 21.9 (4-$CH_3C_6H_4$), 63.1 (OCH_2CH_3), 114.9 (C4), 122.7, 129.0, 129.7, 143.9 (4-$CH_3C_6H_4$), 155.7 (C3), 159.8 (CO_2Et), 173.9 (C5), 184.3 (CHO); **HRMS** (ESI+): calcd for $C_{14}H_{14}NO_4^+$, $[M+H]^+$: 260.0917, found 260.0856.

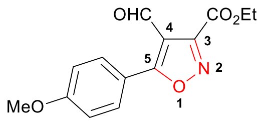

(5e)

3-(ethoxycarbonyl)-4-formyl-5-(4-methoxyphenyl) isoxazole (5e): White solid; 68% yield (93.5 mg); mp 112.3-113.7°C; **^{1}H NMR** (500.13 MHz, $CDCl_3$) δ (ppm) 1.47 (t, 3H, *J* = 7.14 Hz, OCH_2CH_3), 3.89 (s, 3H, 4-$OCH_3C_6H_4$), 4.53 (q, 2H, *J* = 7.15 Hz, OCH_2CH_3), 7.02 (d, 2H, *J* = 9.03 Hz, 4-$OCH_3C_6H_4$), 8.21 (d, 2H, *J* = 9.08 Hz, 4-$OCH_3C_6H_4$), 10.37 (s, 1H, CHO); **^{13}C NMR** (125.77 MHz, $CDCl_3$) δ (ppm) 14.1 (OCH_2CH_3), 55.6 (4-$OCH_3C_6H_4$), 62.9 (OCH_2CH_3), 114.1 (C4), 114.3, 118.0, 131.0 (4-$OCH_3C_6H_4$), 155.8 (C3), 159.8 (CO_2Et), 163.3 (4-$OCH_3C_6H_4$), 173.4 (C5), 184.3 (CHO); **HRMS** (ESI+): calcd for $C_{14}H_{14}NO_5^+$, $[M+H]^+$: 276.0866, found 276.0876.

3. References:

(1) Perrin, D. D.; Armarego L. F. in *Purication of Laboratory Chemicals*, Pergamon Press, New York, 3rd edn, **1996**.

(2) Sheldrick, G. M. Acta Cryst. **2008**, *A64*, 112.

(3) Farrugia, L. J. *J. Appl. Crystallogr.* **1997**, *30*, 565.

(4) Rosa, F. A.; Machado, P.; Rossatto, M.; Vargas, P. S.; Bonacorso, H. G.; Zanatta, N.; Martins, M. A. P. *Synlett* **2007**, 3165.

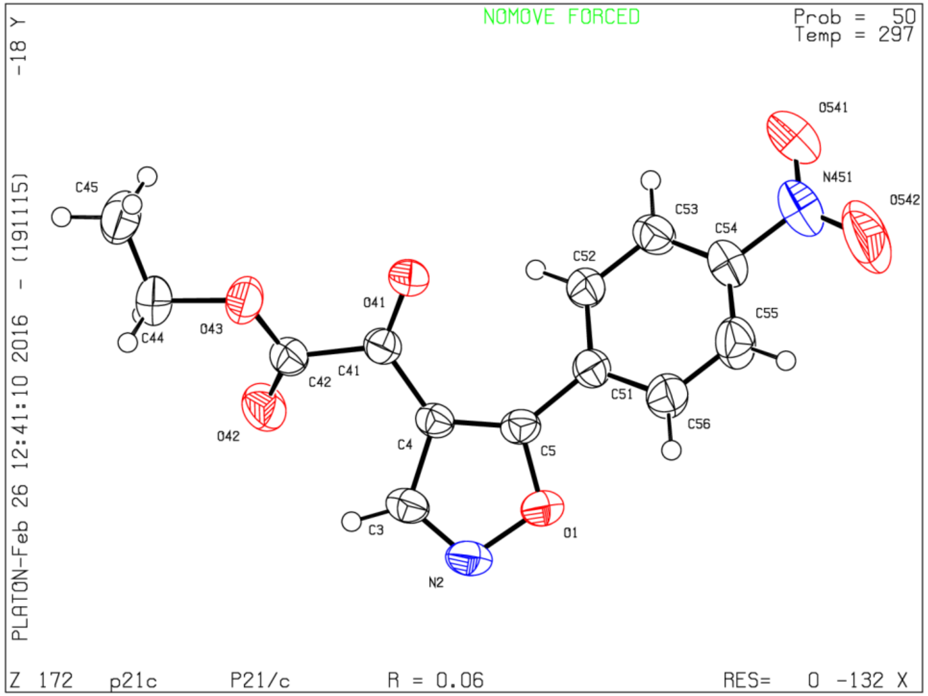

Figure SI-1. ORTEP ploto of compound **2a**

Table SI-1. X-ray crystallographic data of compound **2a**

Bond precision:	C-C = 0.0024 A		Wavelength=0.71073
Cell:	a=12.6326(13)	b=14.5578(13)	c=7.3585(7)
	alpha=90	beta=106.635(3)	gamma=90
Temperature:	297 K		

	Calculated	Reported
Volume	1296.6(2)	1296.6(2)
Space group	P 21/c	P21/c
Hall group	-P 2ybc	-P2ybc
Moiety formula	C13 H10 N2 O6	C13 H10 N2 O6
Sum formula	C13 H10 N2 O6	C13 H10 N2 O6
Mr	290.23	290.23
Dx,g cm-3	1.487	1.487
Z	4	4
Mu (mm-1)	0.120	0.120
F000	600.0	600.0
F000'	600.37	
h,k,lmax	18,20,10	18,20,10
Nref	4003	3988
Tmin,Tmax	0.955,0.972	0.967,0.989
Tmin'	0.955	

Correction method= # Reported T Limits: Tmin=0.967 Tmax=0.989
AbsCorr = MULTI-SCAN

Data completeness= 0.996 Theta(max)= 30.580

R(reflections)= 0.0563(2971) wR2(reflections)= 0.1774(3988)

S = 0.974 Npar= 190

The crystal structure **2a** has been deposited at the Cambridge Crystallographic Data Centre and allocated the deposition number: CCDC-1589617.

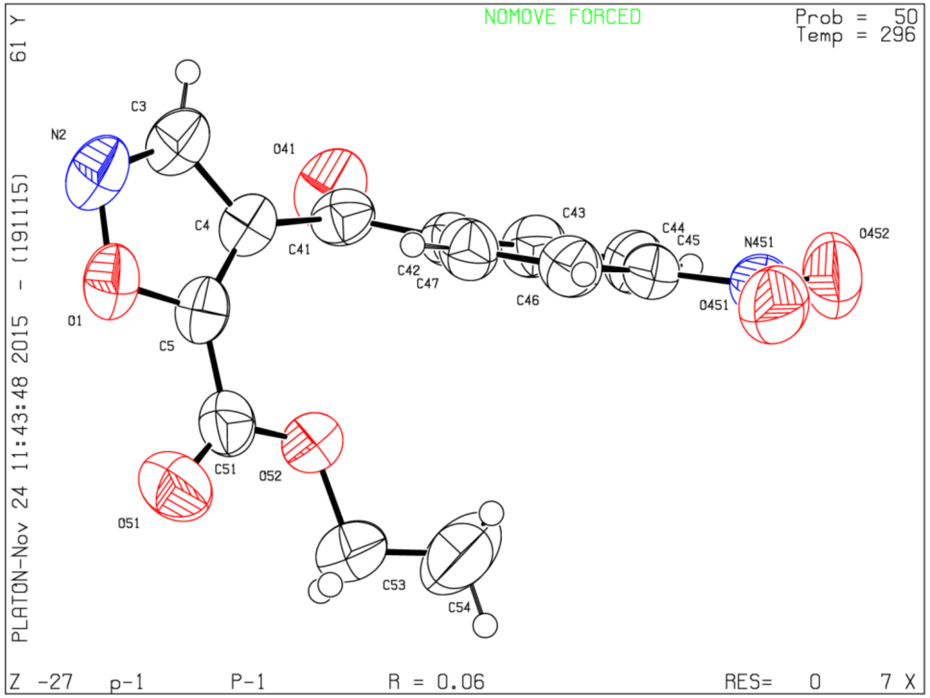

Figure SI-2. ORTEP ploto of compound **3a**

Table SI-2. X-ray crystallographic data of compound **3a**

Bond precision:	C-C = 0.0039 A		Wavelength=1.54178
Cell:	a=6.6907(15)	b=8.7715(16)	c=12.002(4)
	alpha=97.212(18)	beta=93.54(2)	gamma=107.759(18)
Temperature:	296 K		

	Calculated	Reported
Volume	661.8(3)	661.8(3)
Space group	P -1	P-1
Hall group	-P 1	-P1
Moiety formula	C13 H10 N2 O6	C13 H10 N2 O6
Sum formula	C13 H10 N2 O6	C13 H10 N2 O6
Mr	290.23	290.23
Dx,g cm-3	1.457	1.457
Z	2	2
Mu (mm-1)	1.011	1.011
F000	300.0	300.0
F000'	301.14	
h,k,lmax	8,10,14	8,10,14
Nref	2637	2633
Tmin,Tmax	0.739,0.817	0.659,0.823
Tmin'	0.604	

Correction method= # Reported T Limits: Tmin=0.659 Tmax=0.823
AbsCorr = MULTI-SCAN

Data completeness= 0.998 Theta(max)= 72.580

R(reflections)= 0.0600(1815) wR2(reflections)= 0.1853(2633)

S = 1.062 Npar= 191

The crystal structure **3a** has been deposited at the Cambridge Crystallographic Data Centre and allocated the deposition number: CCDC-1589618.

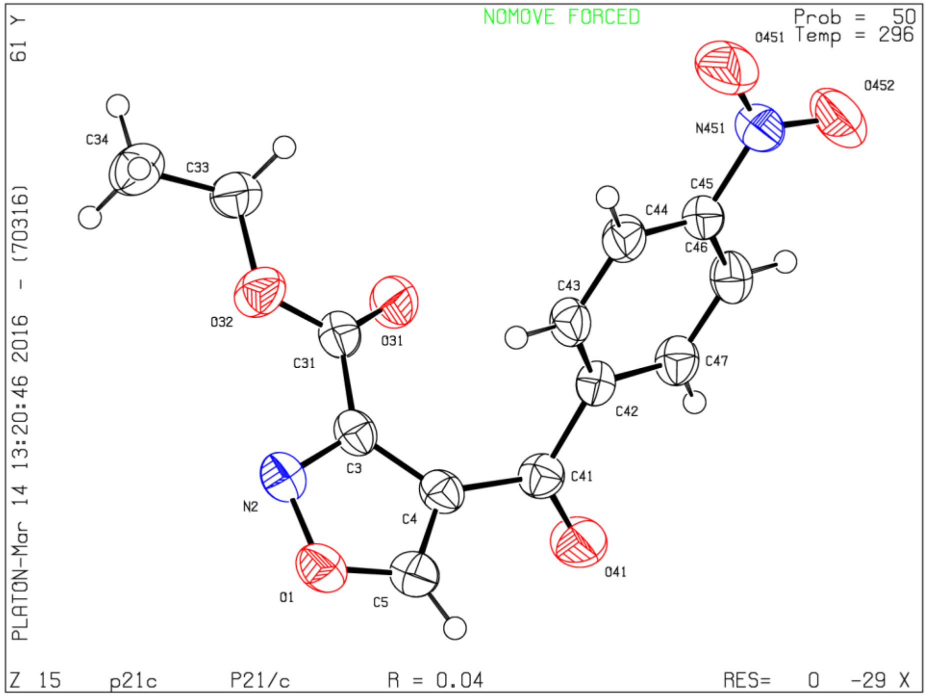

Figure SI-3. ORTEP ploto of compound **4a**

Table SI-3. X-ray crystallographic data of compound **4a**

Bond precision:	C-C = 0.0020 A		Wavelength=1.54178
Cell:	a=7.3487(2)	b=17.1804(4)	c=10.4508(3)
	alpha=90	beta=98.167(1)	gamma=90
Temperature:	296 K		

	Calculated	Reported
Volume	1306.07(6)	1306.07(6)
Space group	P 21/c	P21/c
Hall group	-P 2ybc	-P2ybc
Moiety formula	C13 H10 N2 O6	C13 H10 N2 O6
Sum formula	C13 H10 N2 O6	C13 H10 N2 O6
Mr	290.23	290.23
Dx,g cm-3	1.476	1.476
Z	4	4
Mu (mm-1)	1.025	1.025
F000	600.0	600.0
F000'	602.27	
h,k,lmax	9,21,12	9,21,12
Nref	2573	2565
Tmin,Tmax	0.704,0.751	0.691,0.762
Tmin'	0.639	

Correction method= # Reported T Limits: Tmin=0.691 Tmax=0.762
AbsCorr = MULTI-SCAN

Data completeness= 0.997 Theta(max)= 72.240

R(reflections)= 0.0403(2260) wR2(reflections)= 0.1095(2565)

S = 1.050 Npar= 190

The crystal structure **4a** has been deposited at the Cambridge Crystallographic Data Centre and allocated the deposition number: CCDC-1589619.

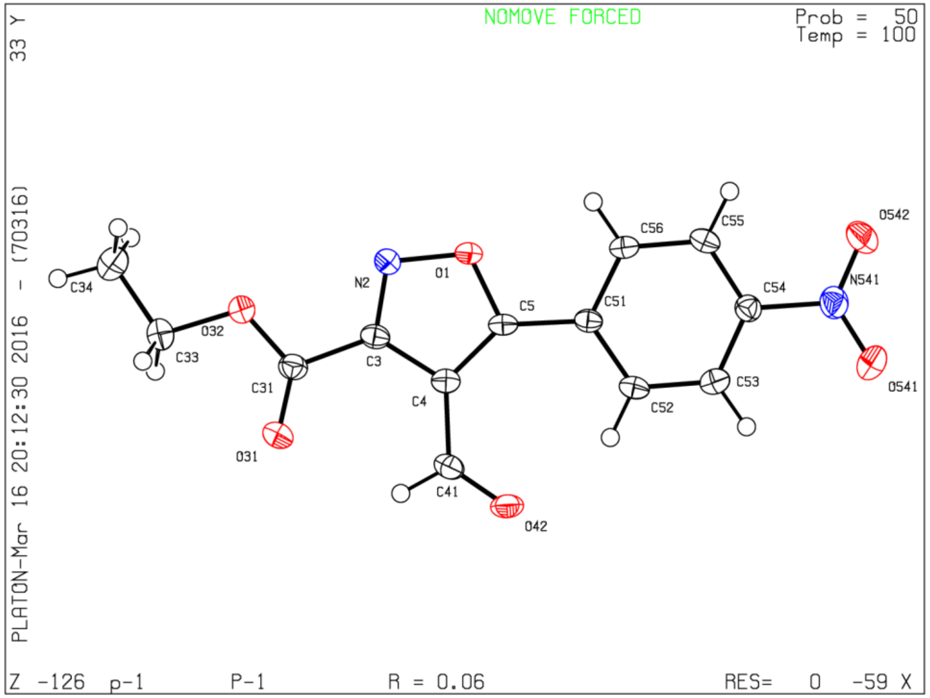

Figure SI-4. ORTEP ploto of compound **5a**

Table SI-4. X-ray crystallographic data of compound **5a**

```
Bond precision:   C-C = 0.0028 A          Wavelength=0.71073

Cell:          a=5.8104(5)      b=8.2881(8)      c=13.1190(12)
               alpha=89.312(3)  beta=82.695(3)   gamma=85.322(3)
Temperature:   100 K

               Calculated                  Reported
Volume         624.56(10)                  624.56(10)
Space group    P -1                        P-1
Hall group     -P 1                        -P1
Moiety formula C13 H10 N2 O6               C13 H10 N2 O6
Sum formula    C13 H10 N2 O6               C13 H10 N2 O6
Mr             290.23                      290.23
Dx,g cm-3      1.543                       1.543
Z              2                           2
Mu (mm-1)      0.125                       0.125
F000           300.0                       300.0
F000'          300.19
h,k,lmax       8,11,18                     8,11,18
Nref           3637                        3625
Tmin,Tmax      0.963,0.979                 0.935,0.969
Tmin'          0.944

Correction method= # Reported T Limits: Tmin=0.935 Tmax=0.969
AbsCorr = MULTI-SCAN

Data completeness= 0.997          Theta(max)= 29.980

R(reflections)= 0.0560( 2149)     wR2(reflections)= 0.1556( 3625)

S = 1.045                Npar= 190
```

The crystal structure **5a** has been deposited at the Cambridge Crystallographic Data Centre and allocated the deposition number: CCDC-1589620.

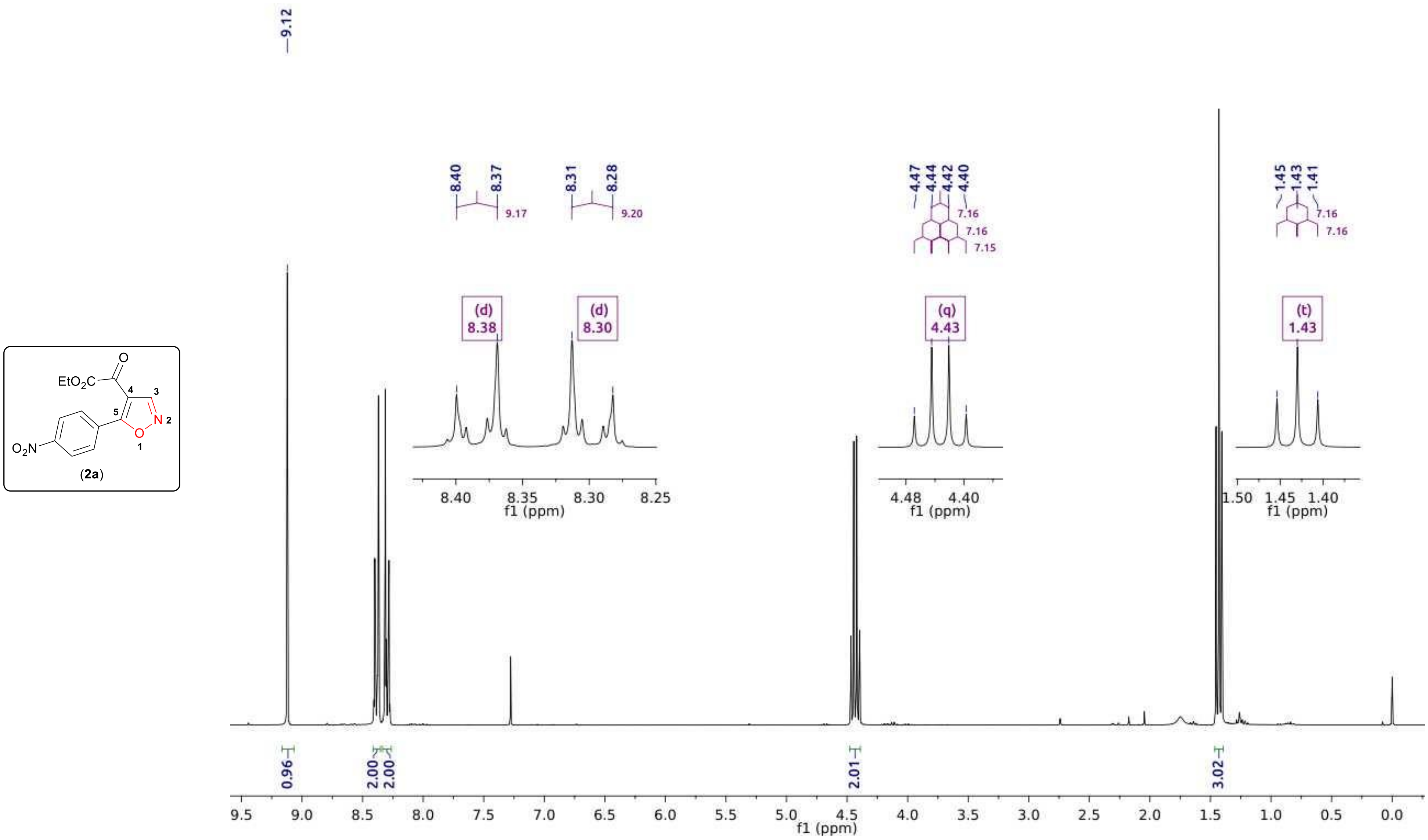

Figure SI-5. ^{1}H NMR spectrum of **2a** ($CDCl_3$, 300.06 MHz)

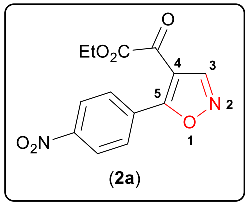

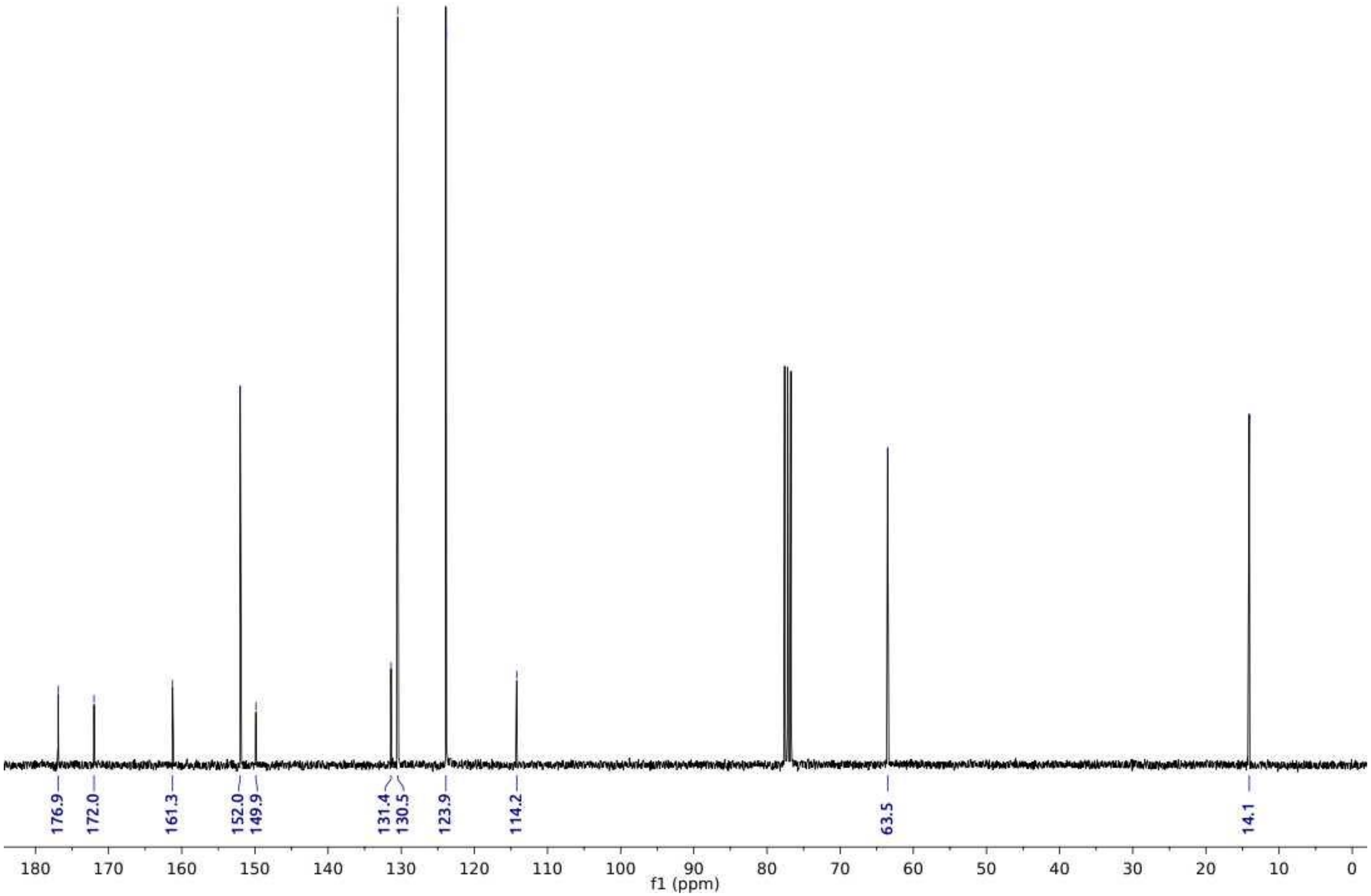

Figure SI-6. ^{13}C NMR spectrum of **2a** ($CDCl_3$, 75.46 MHz)

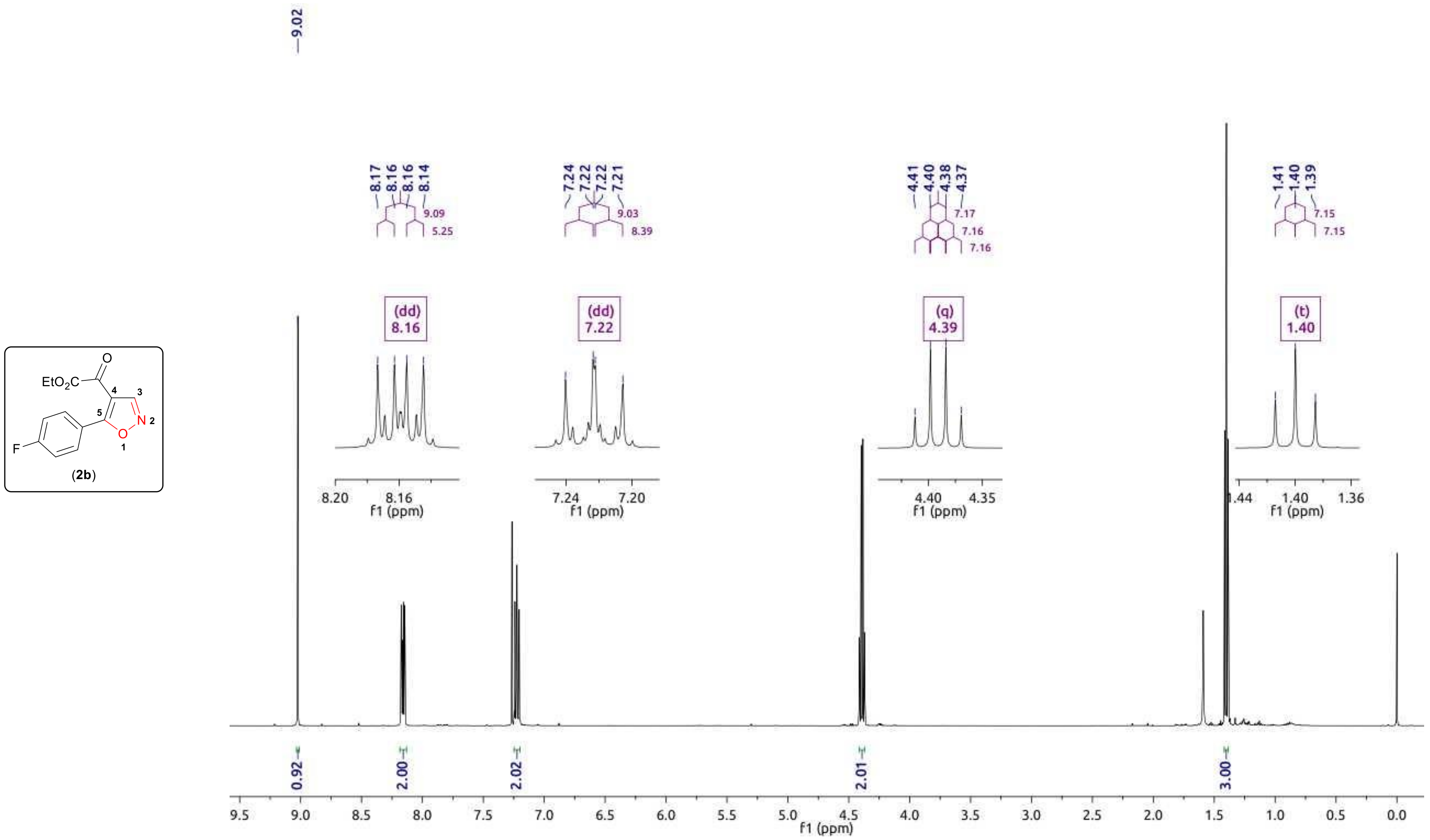

Figure SI-7. ^{1}H NMR spectrum of **2b** ($CDCl_3$, 500.13 MHz)

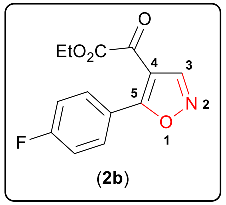

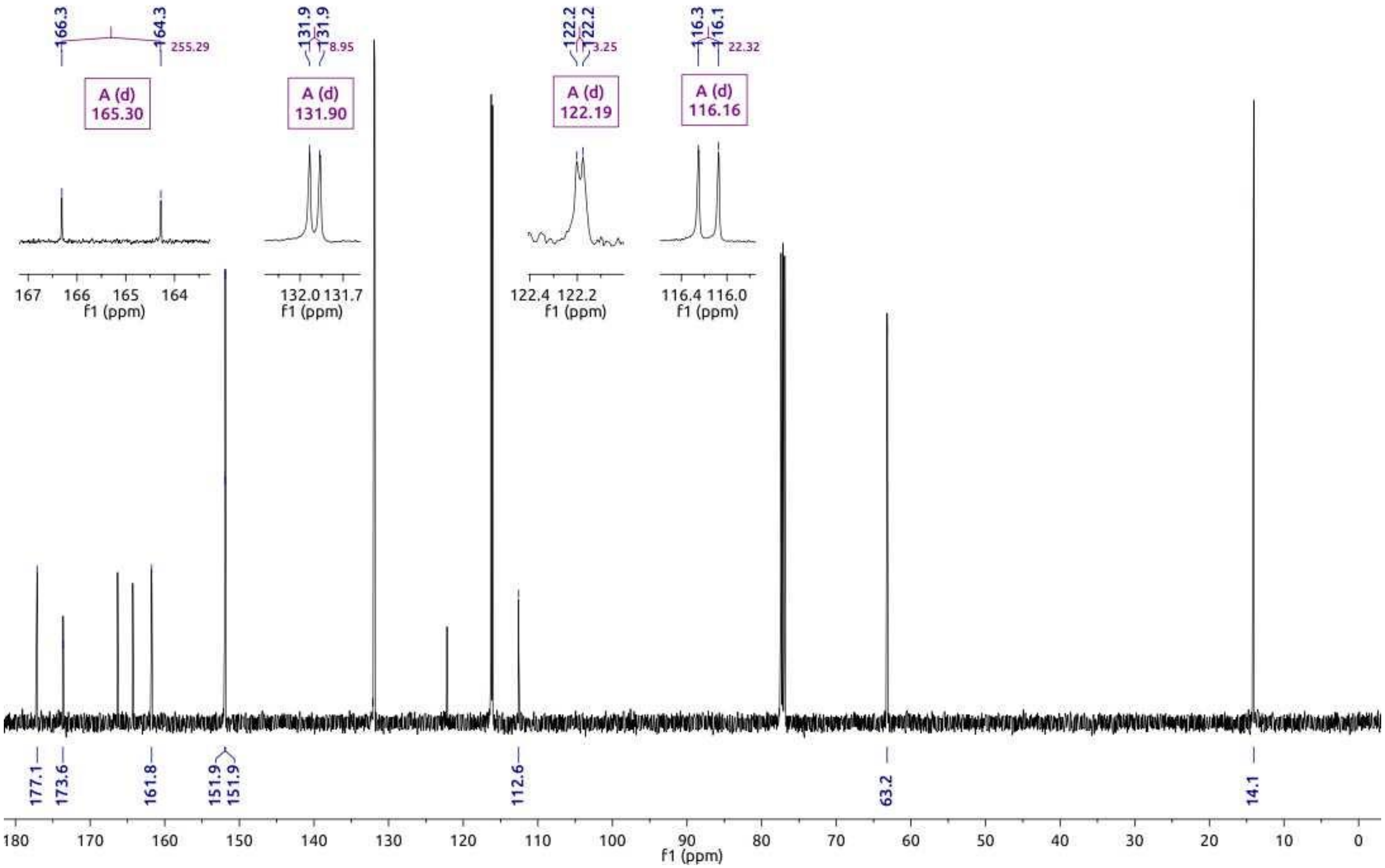

Figure SI-8. ^{13}C NMR spectrum of **2b** ($CDCl_3$, 125.77 MHz)

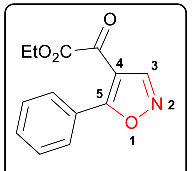

(**2c**)

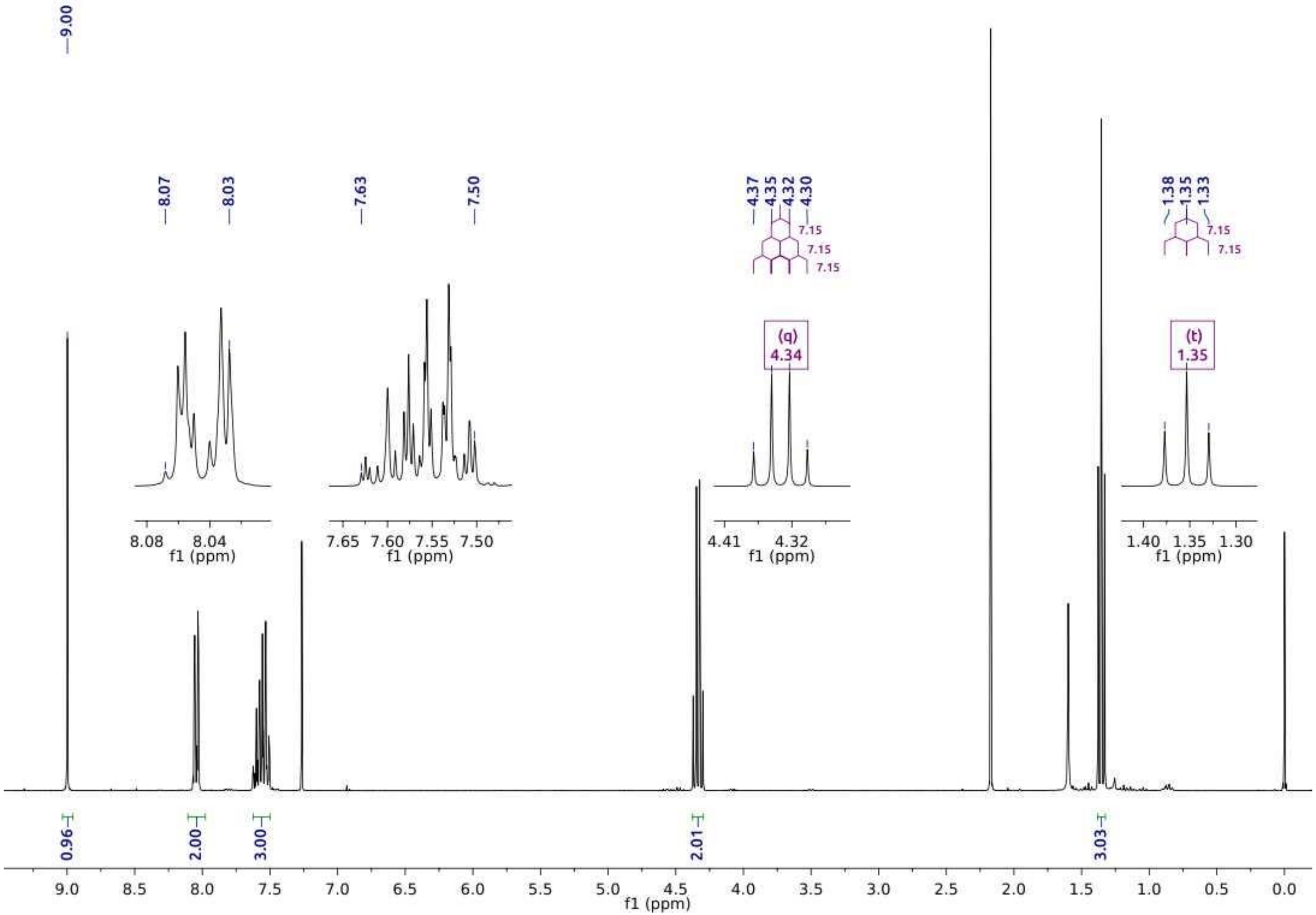

Figure SI-9. ^{1}H NMR spectrum of **2c** ($CDCl_3$, 300.06 MHz)

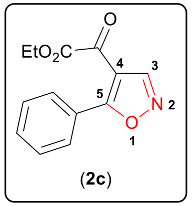

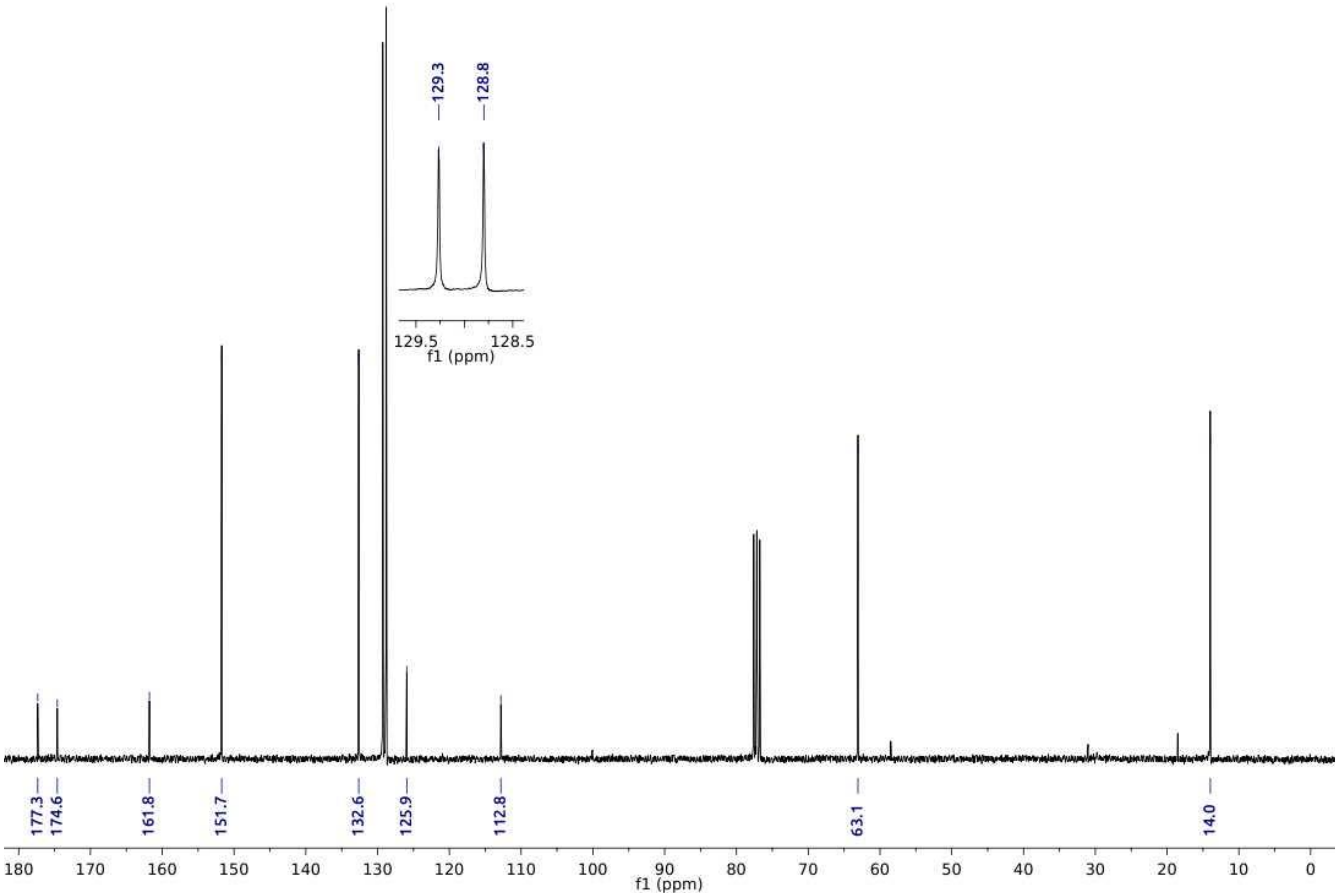

Figure SI-10. ^{13}C NMR spectrum of **2c** ($CDCl_3$, 75.46 MHz)

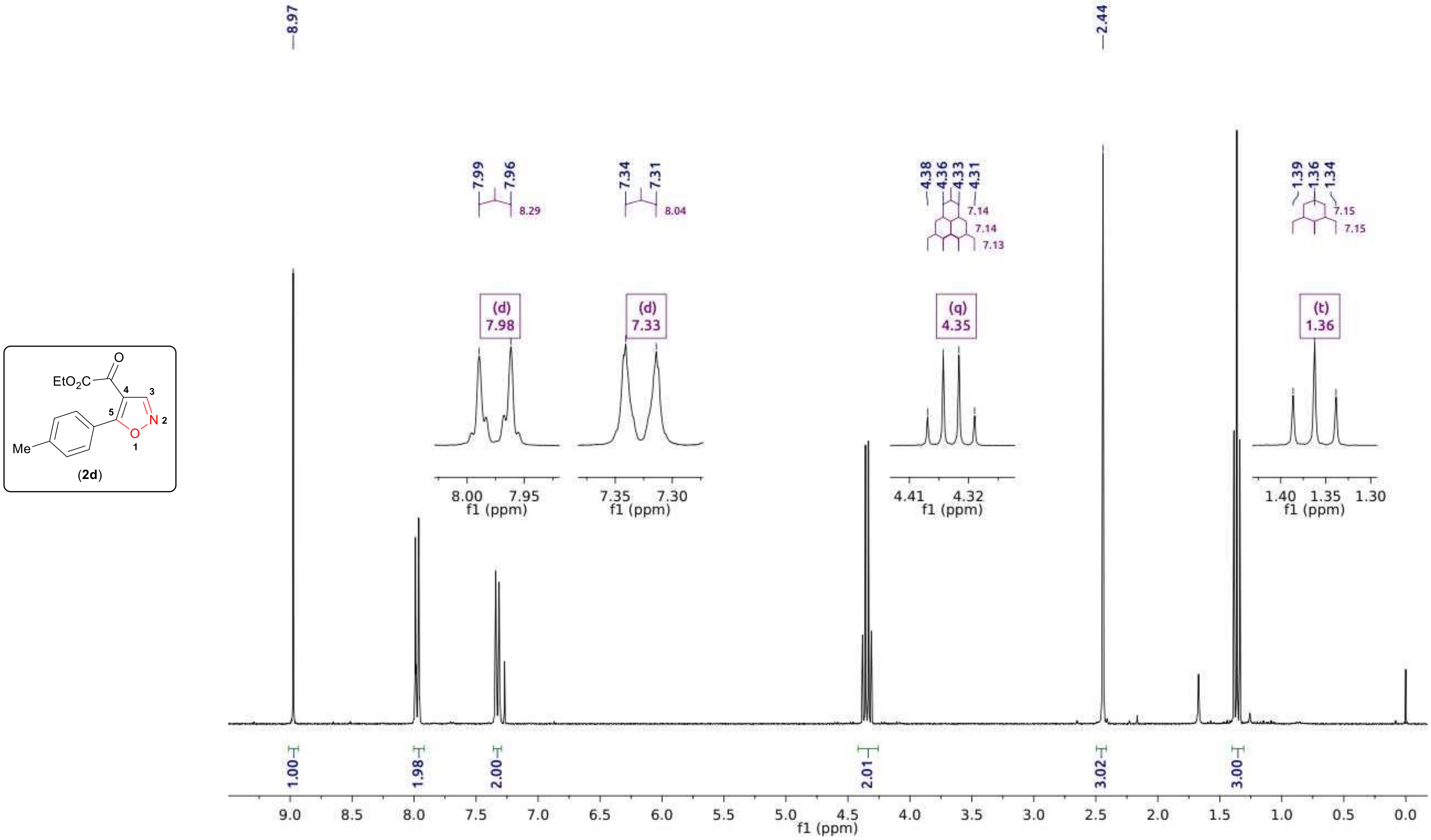

Figure SI-11. ^{1}H NMR spectrum of **2d** ($CDCl_3$, 300.06 MHz)

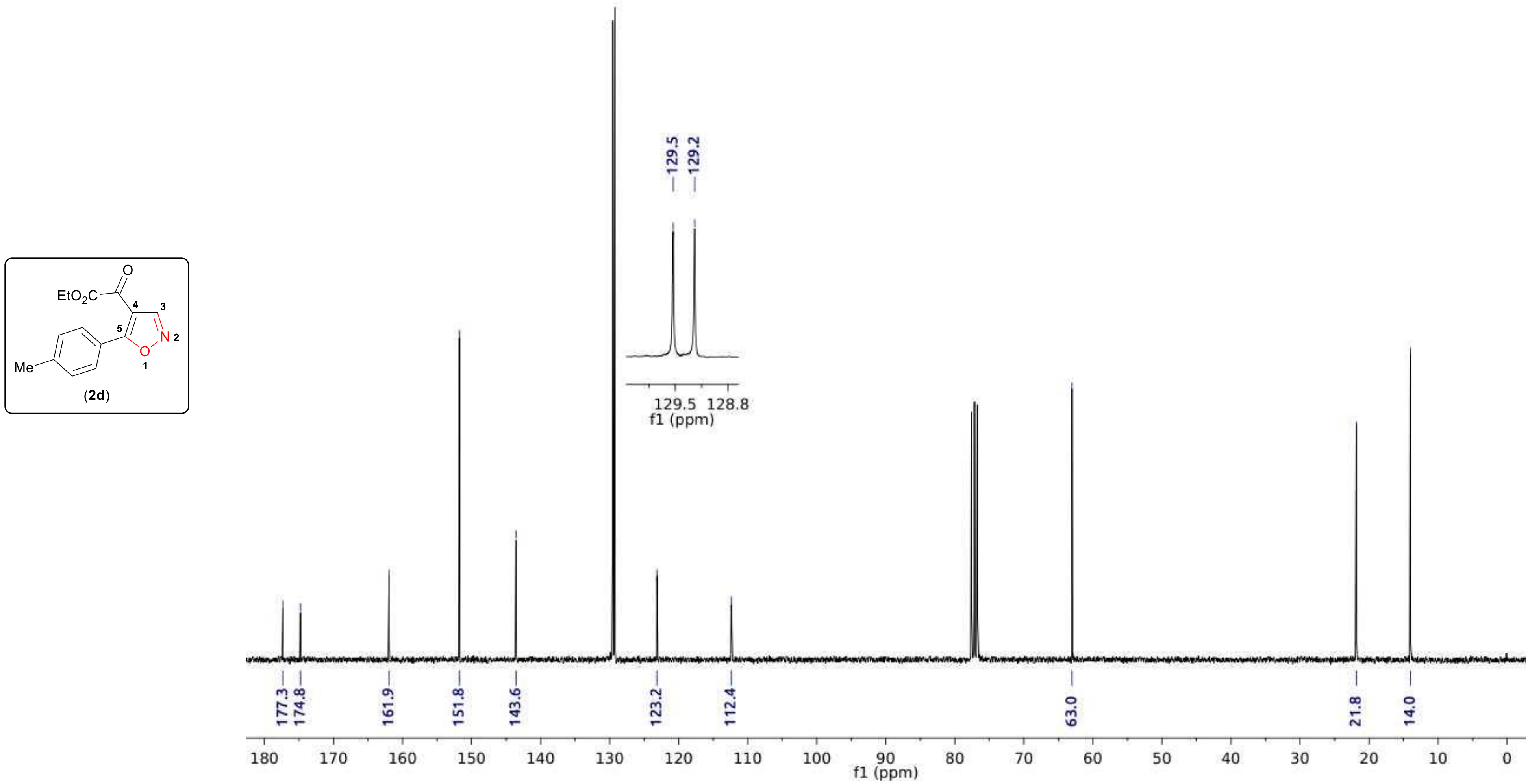

Figure SI-12. ^{13}C NMR spectrum of **2d** ($CDCl_3$, 75.46 MHz)

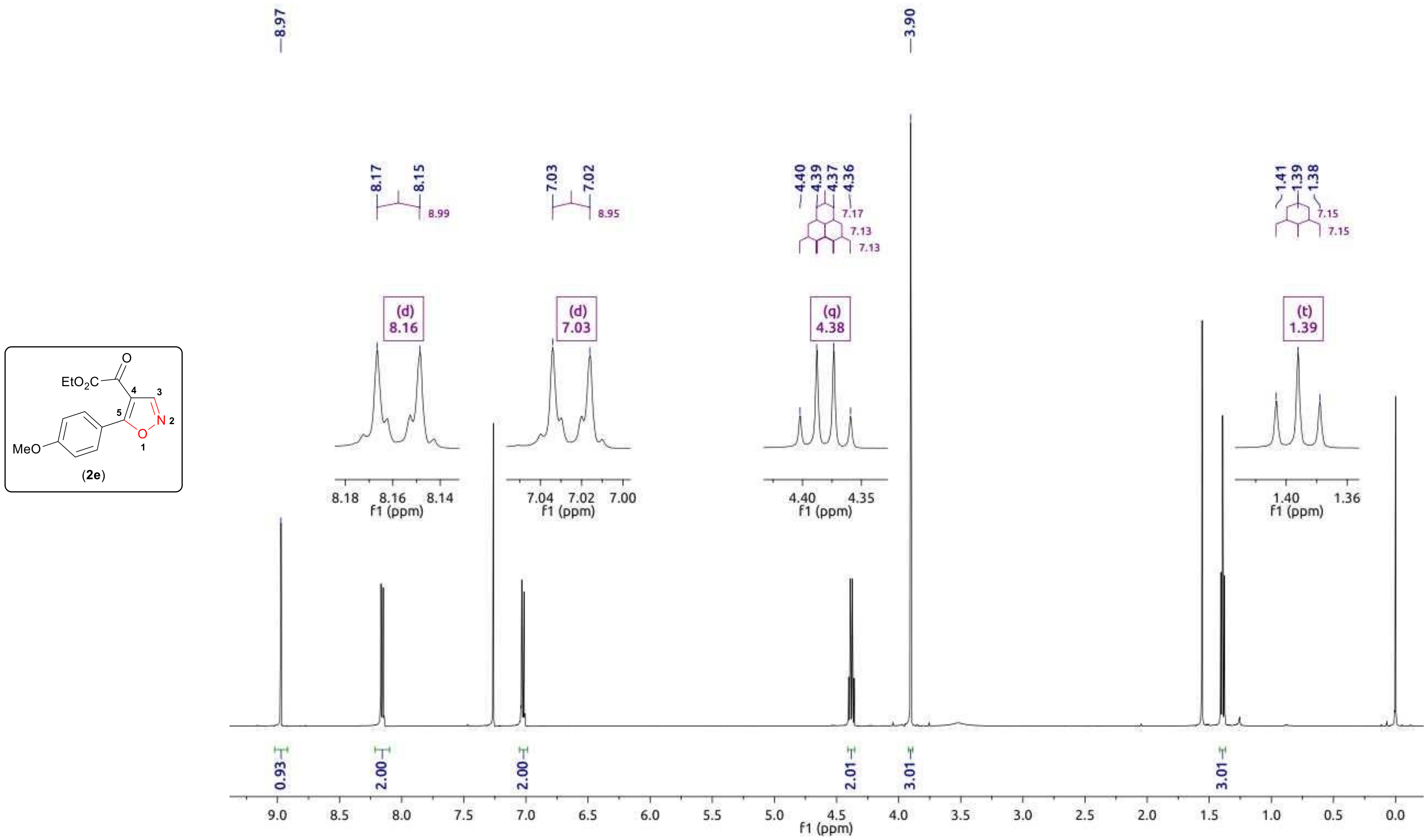

Figure SI-13. ^{1}H NMR spectrum of **2e** ($CDCl_3$, 500.13 MHz)

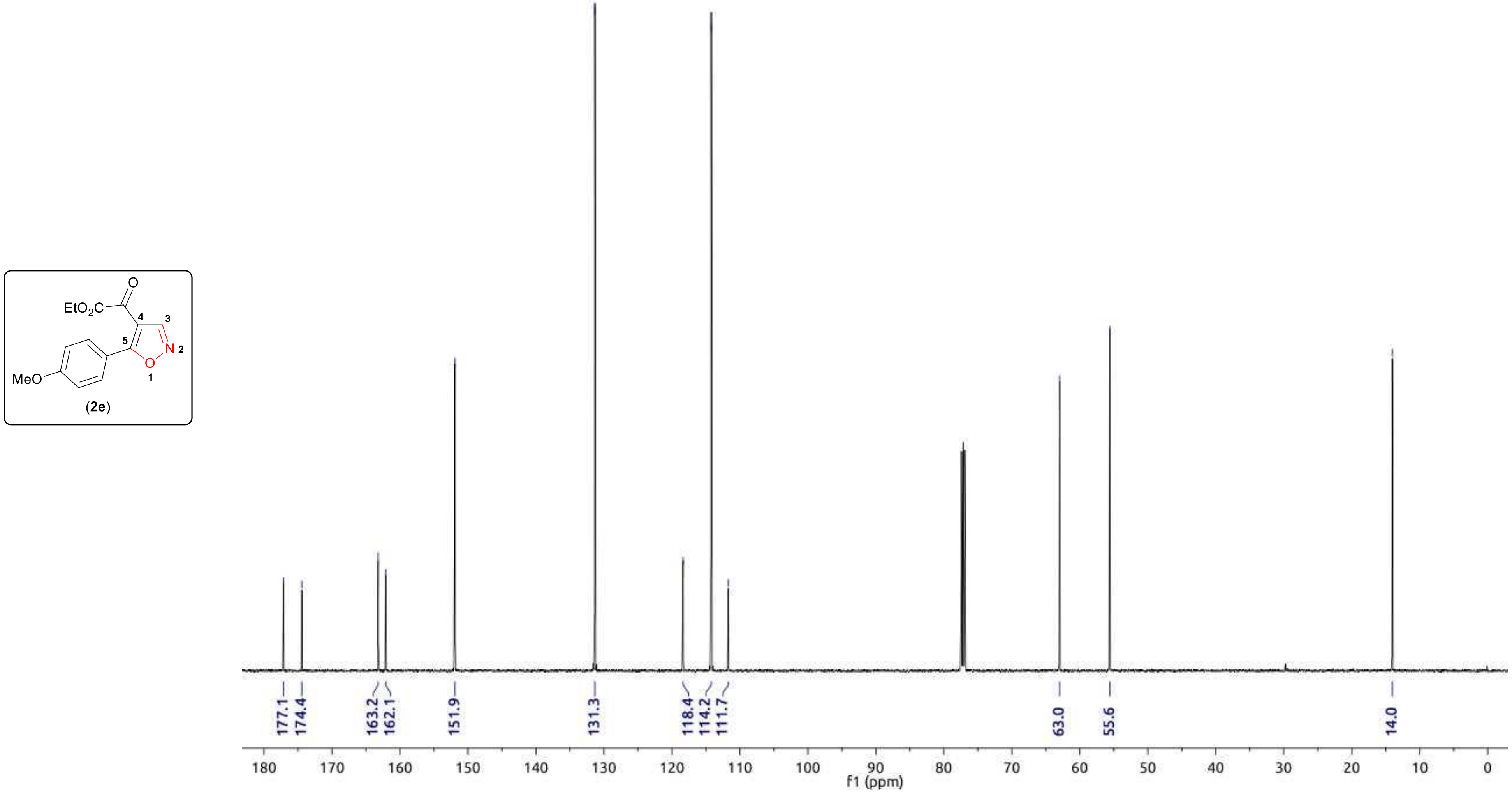

Figure SI-14. ^{13}C NMR spectrum of **2e** ($CDCl_3$, 125.77 MHz)

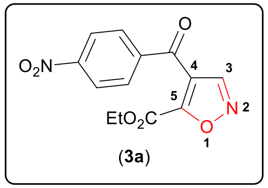

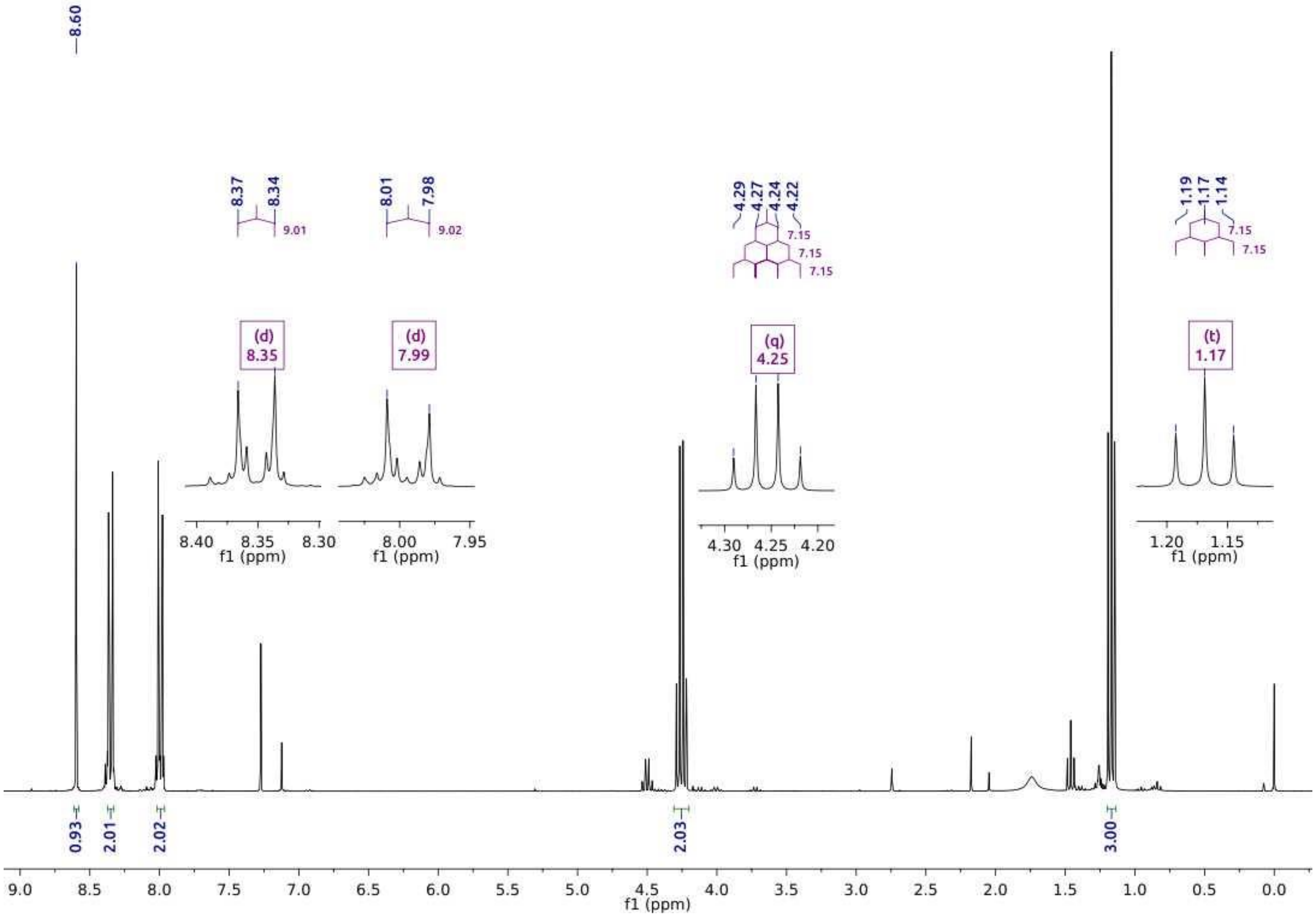

Figure SI-15. ^1H NMR spectrum of **3a** ($CDCl_3$, 300.06 MHz)

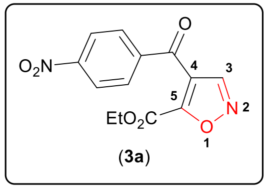

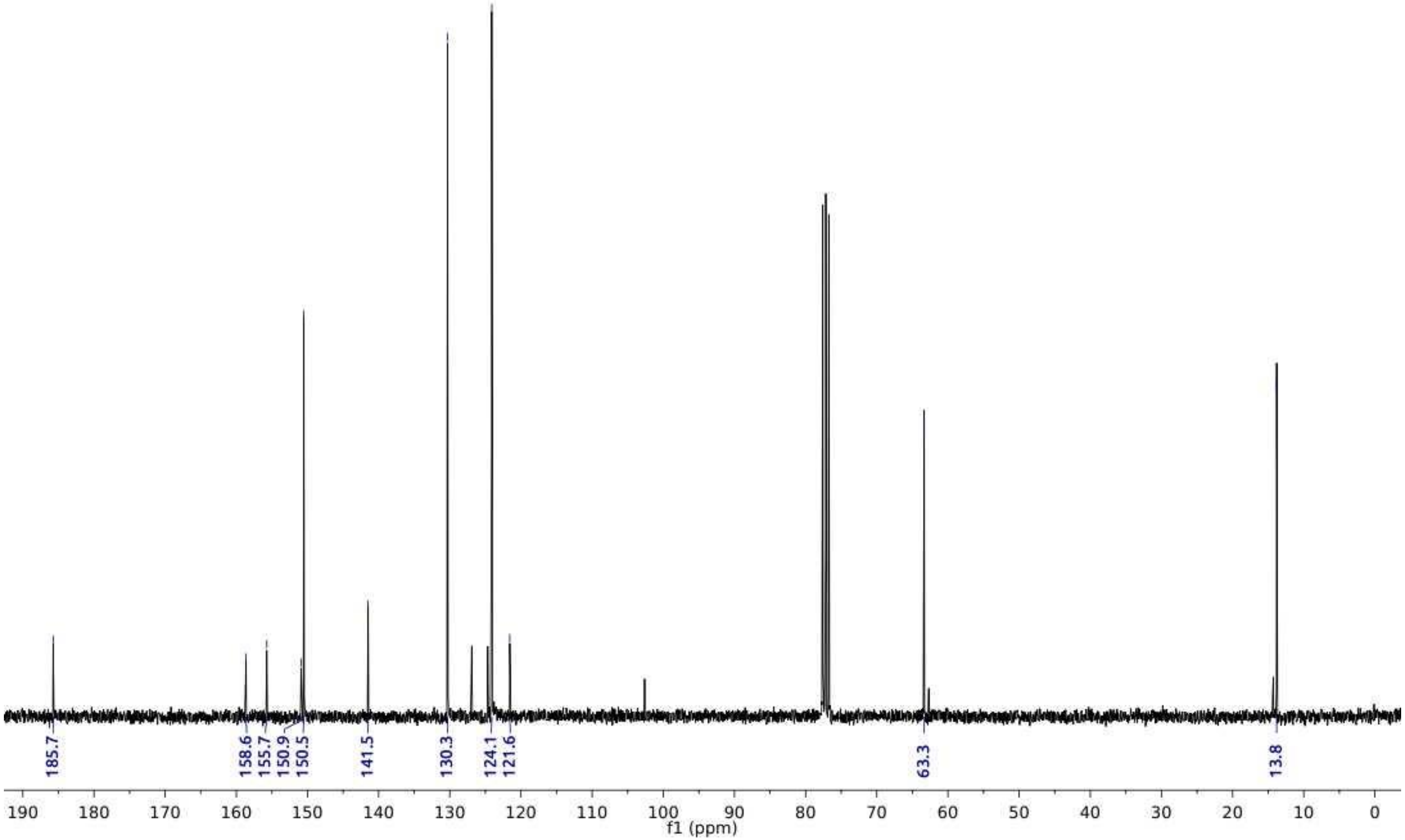

Figure SI-16. ^{13}C NMR spectrum of **3a** ($CDCl_3$, 75.46 MHz)

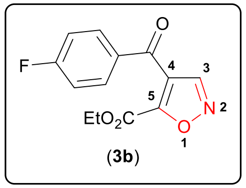

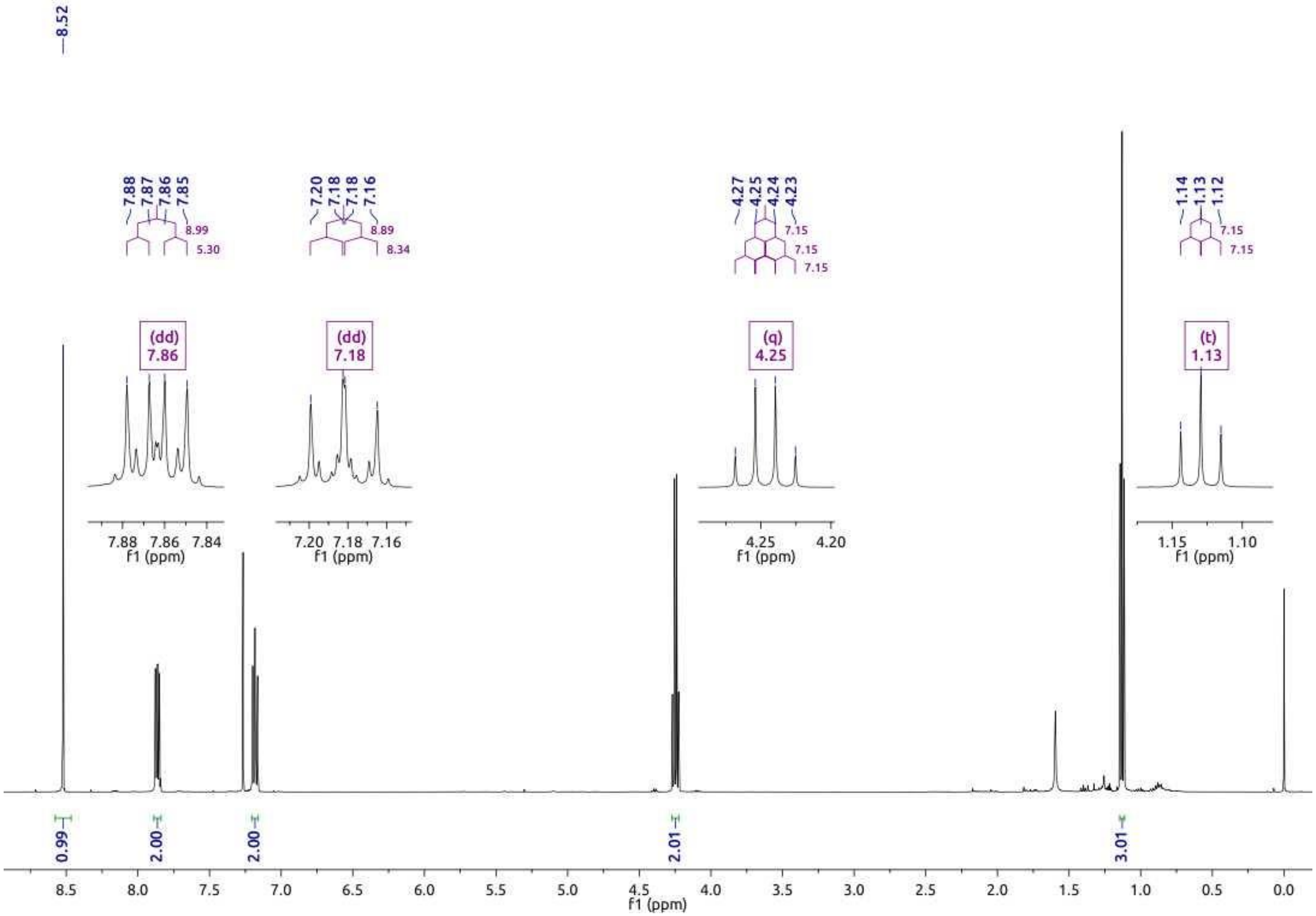

Figure SI-17. ^{1}H NMR spectrum of **3b** ($CDCl_3$, 500.13 MHz)

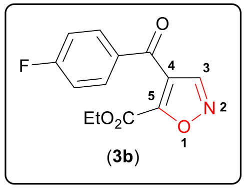

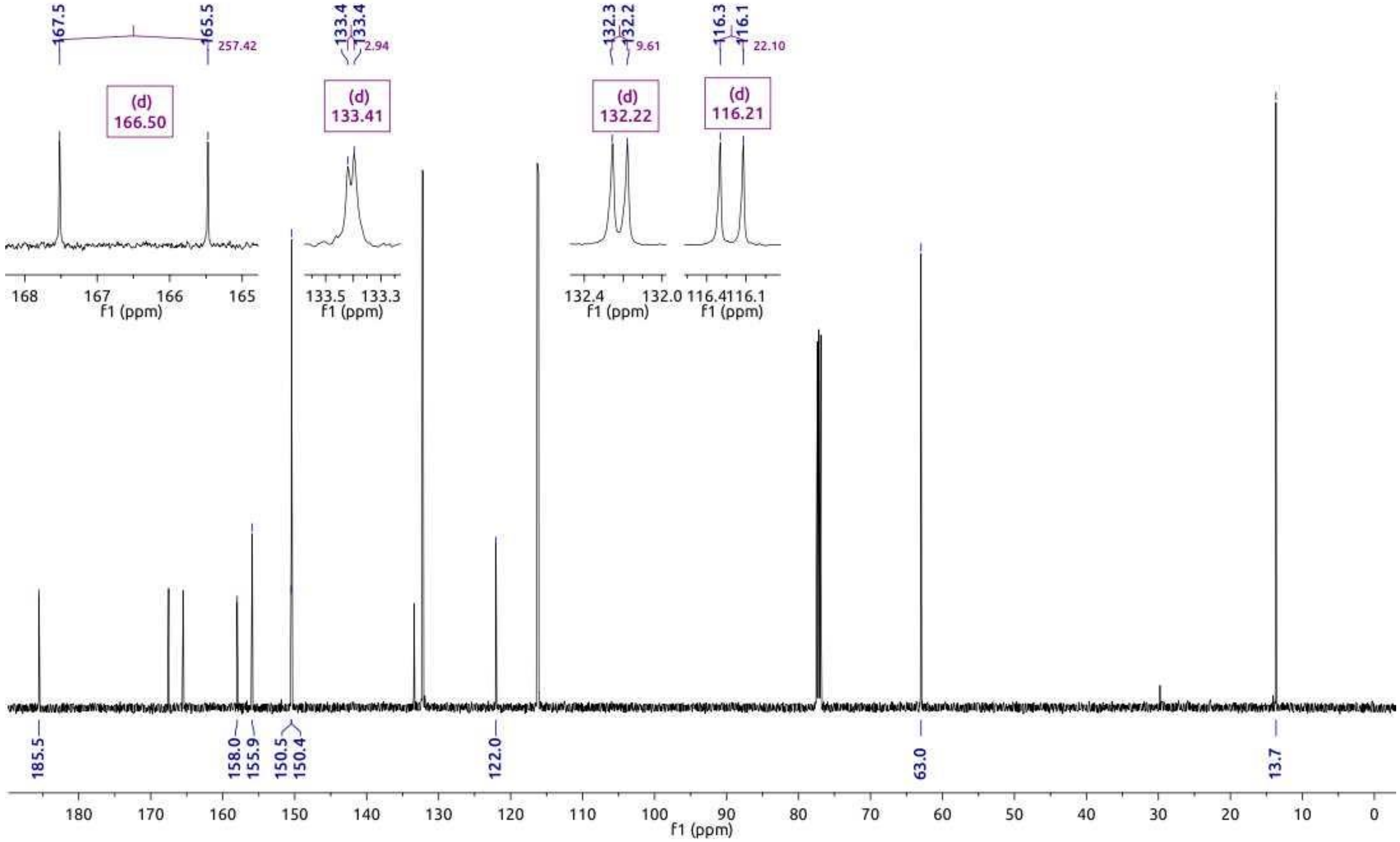

Figure SI-18. ^{13}C NMR spectrum of **3b** ($CDCl_3$, 125.77 MHz)

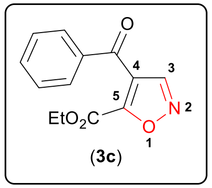

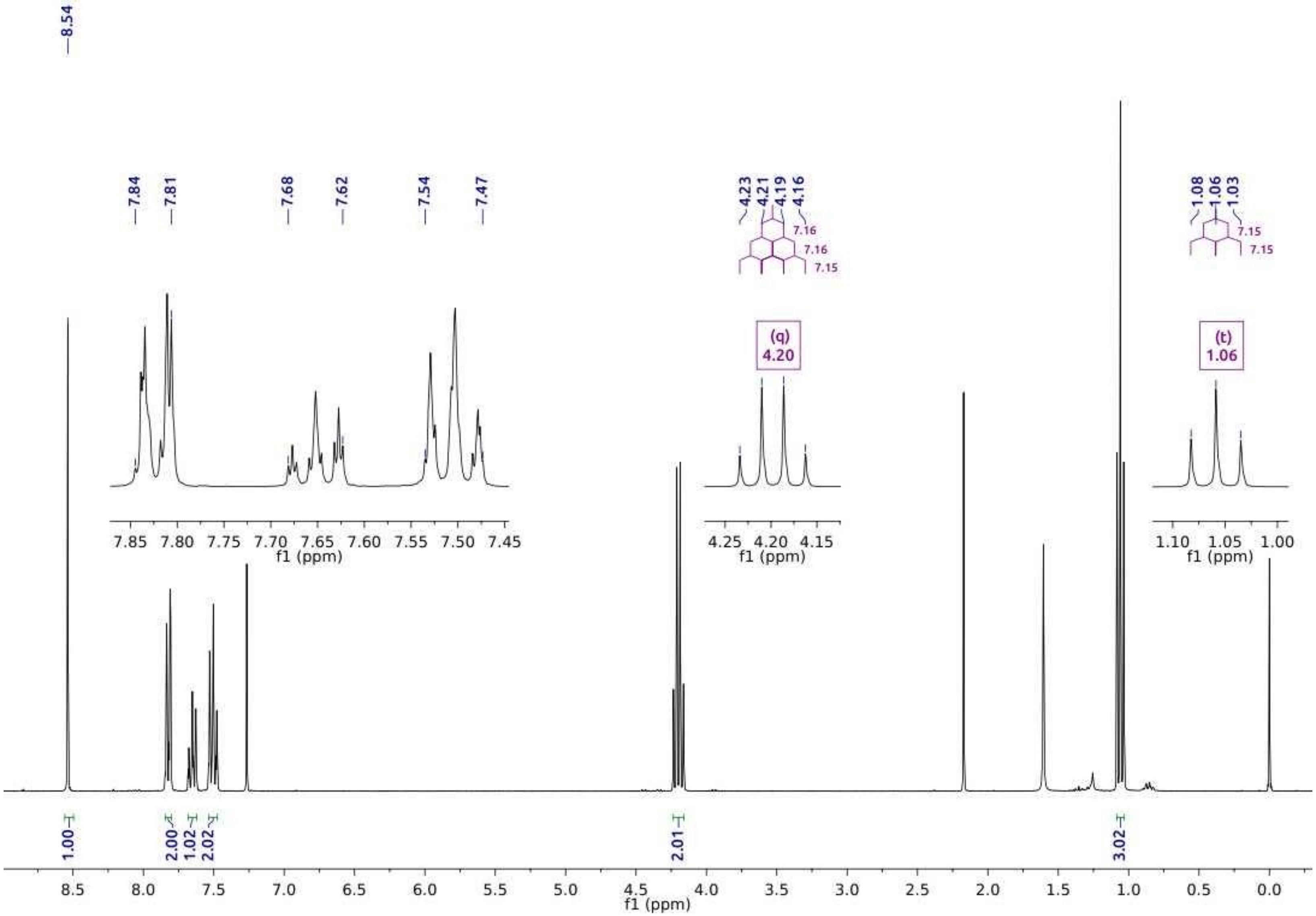

Figure SI-19. ^{1}H NMR spectrum of **3c** ($CDCl_3$, 300.06 MHz)

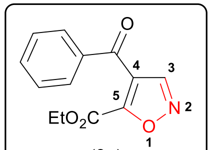

(**3c**)

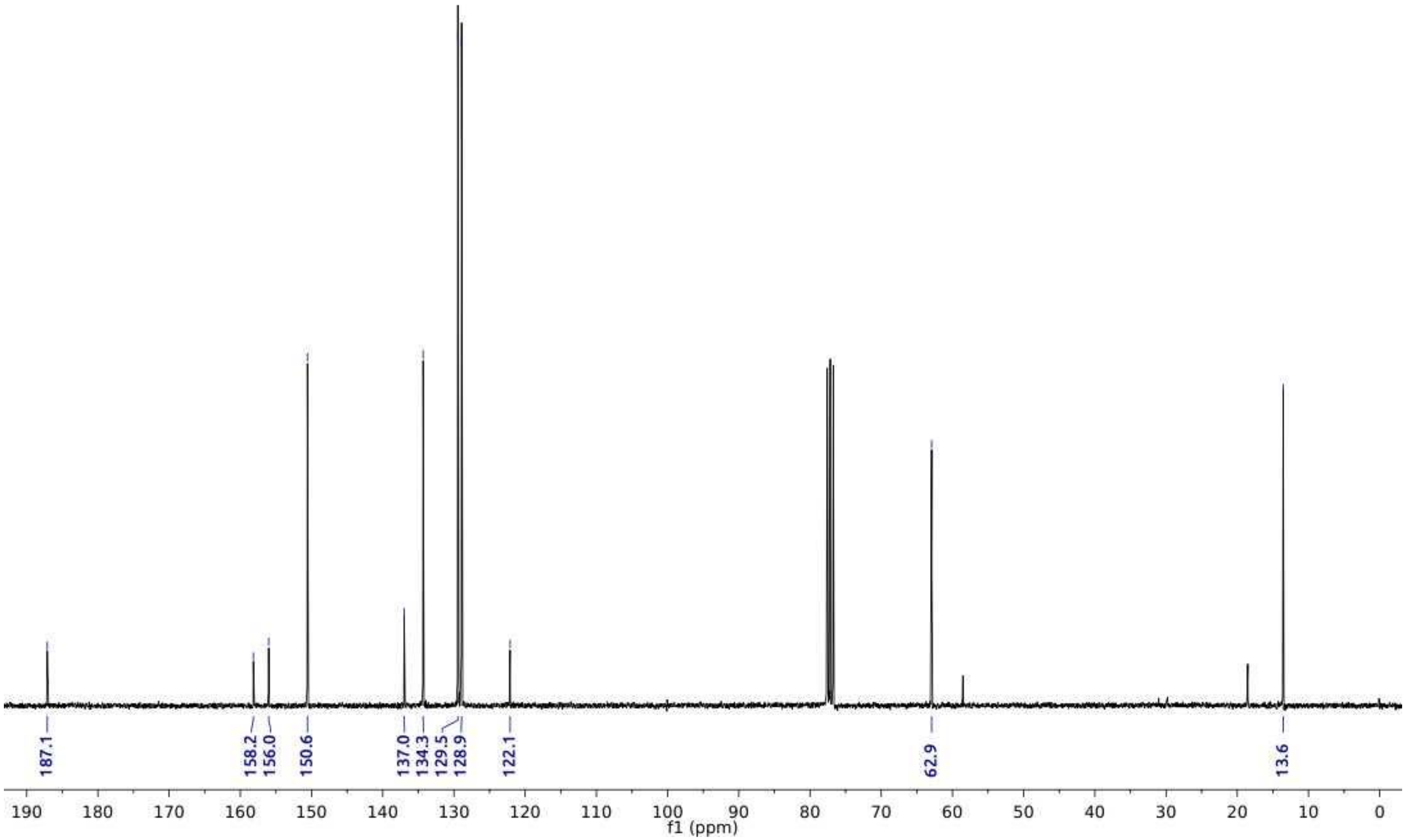

Figure SI-20. ^{13}C NMR spectrum of **3c** ($CDCl_3$, 75.46 MHz)

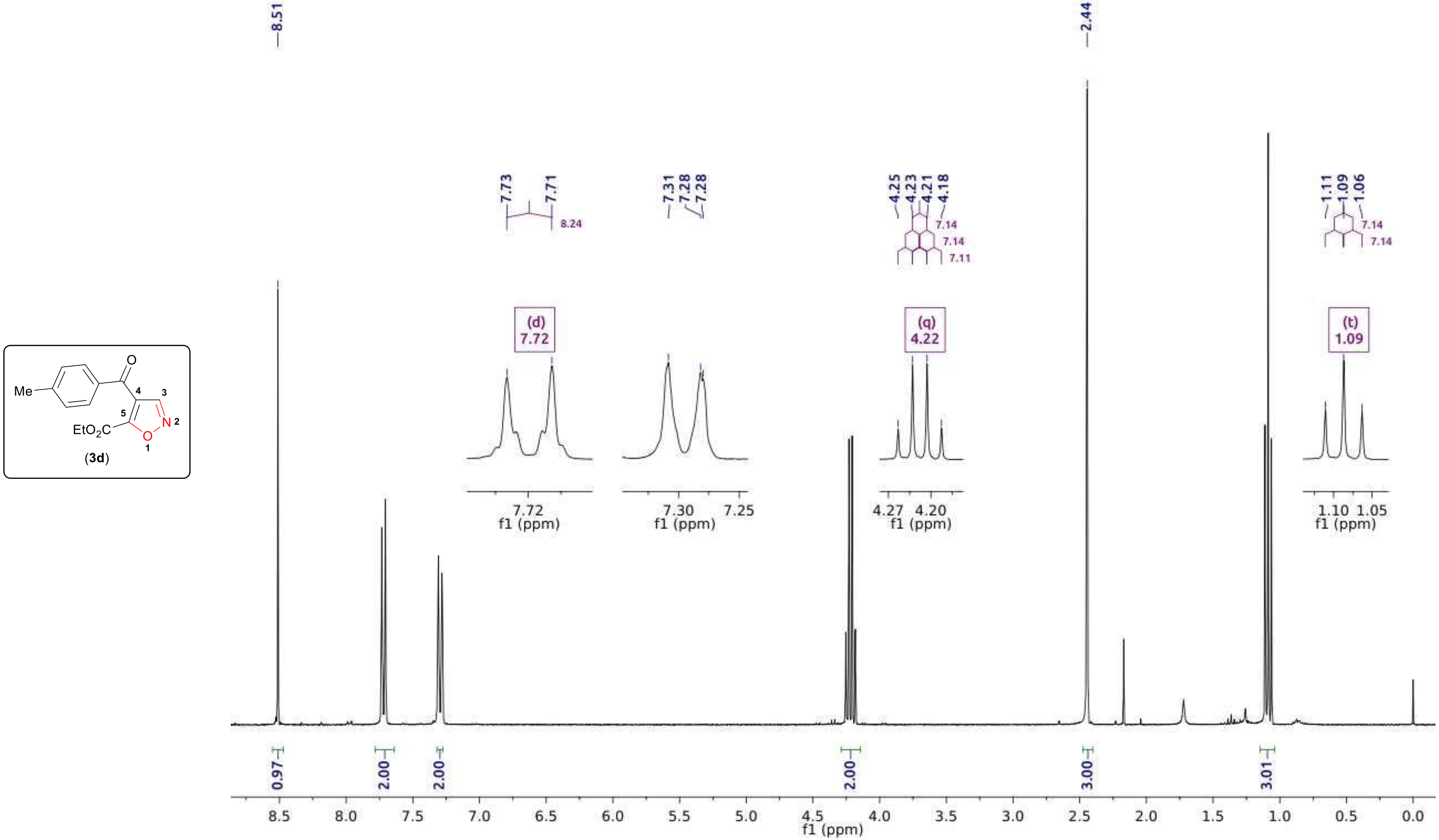

Figure SI-21. ^{1}H NMR spectrum of **3d** ($CDCl_3$, 300.06 MHz)

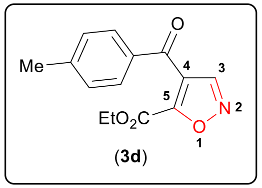

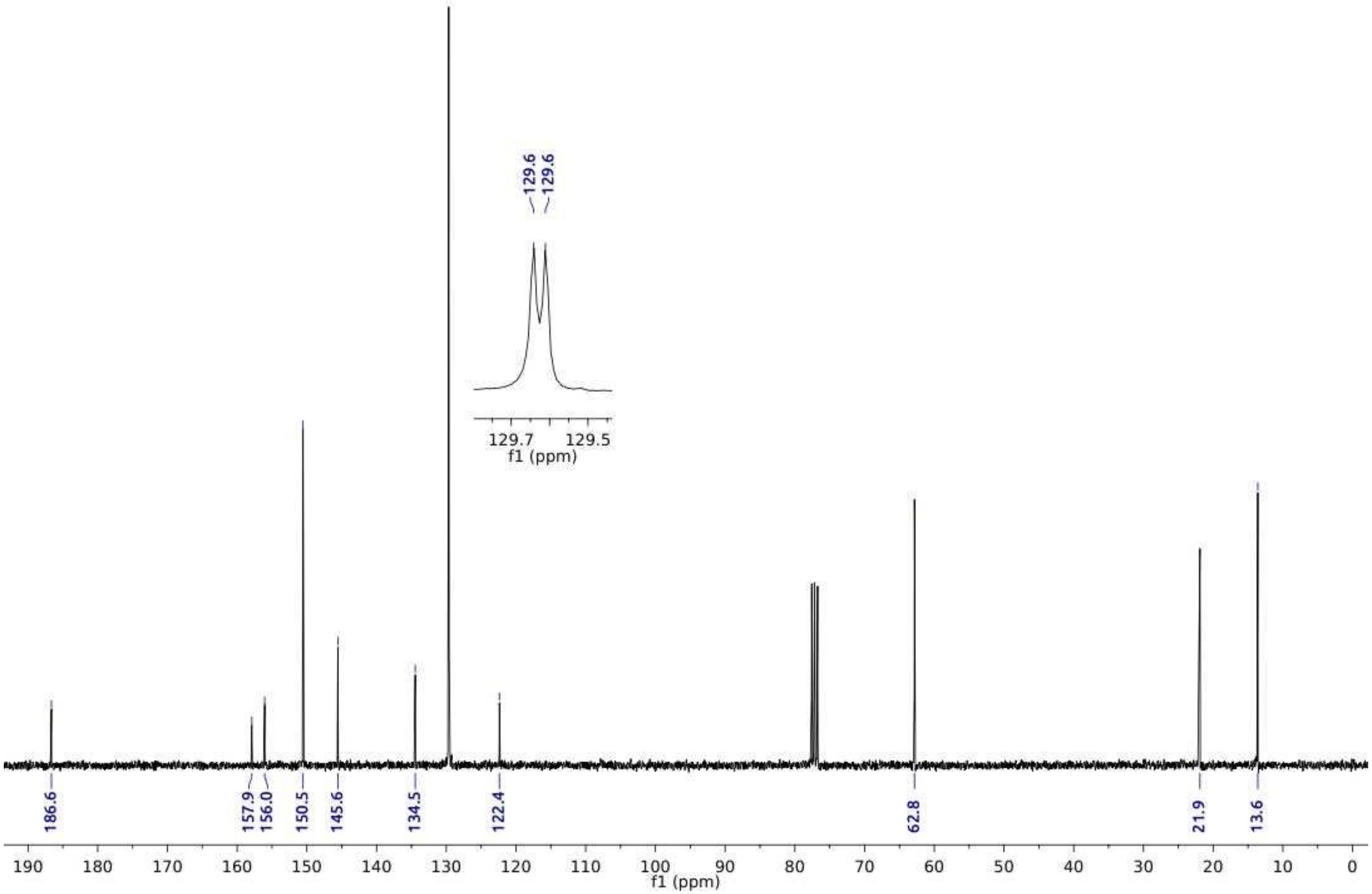

Figure SI-22. ^{13}C NMR spectrum of **3d** ($CDCl_3$, 75.46 MHz)

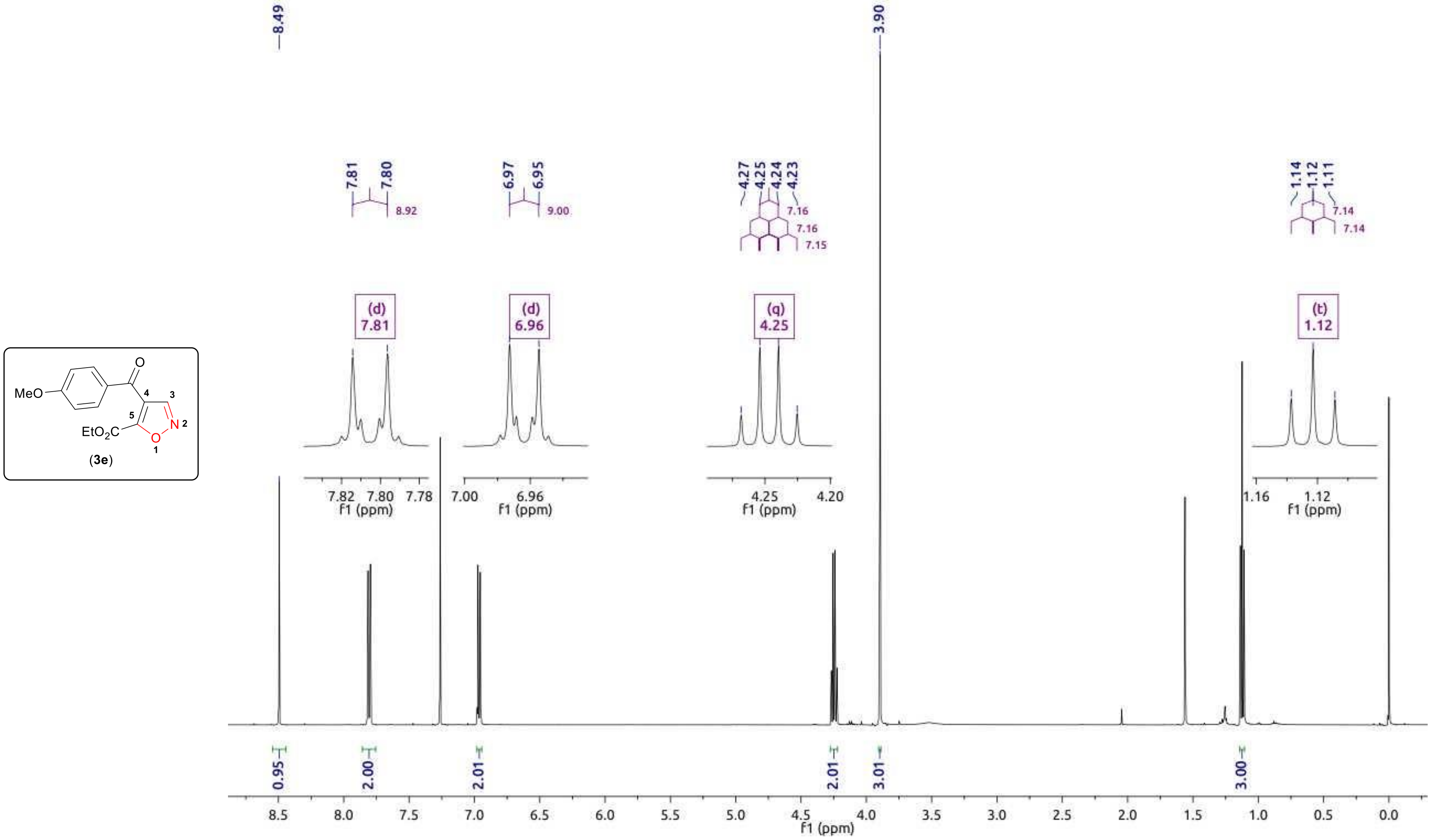

Figure SI-23. ^{1}H NMR spectrum of **3e** ($CDCl_3$, 500.13 MHz)

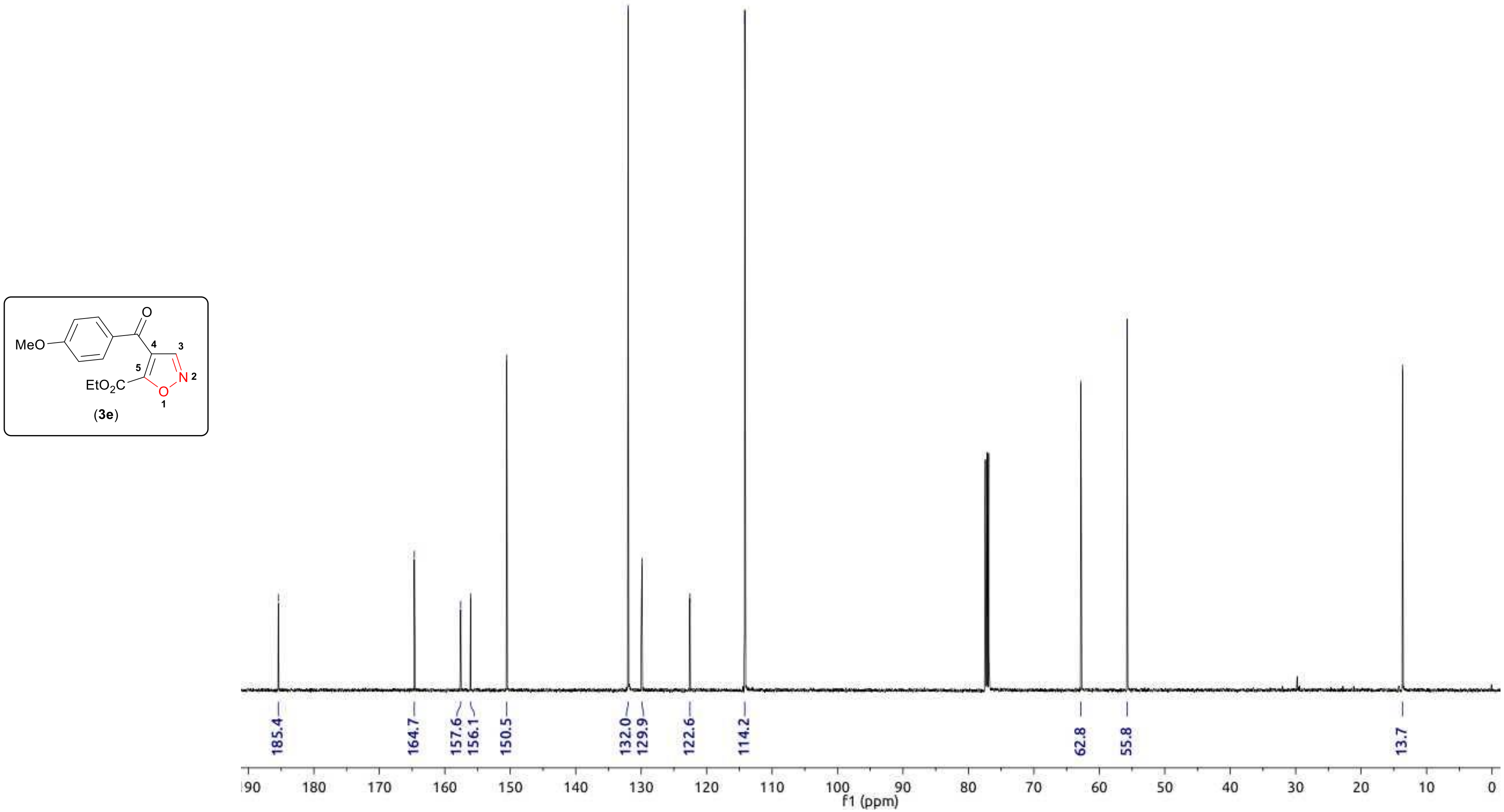

Figure SI-24. ^{13}C NMR spectrum of **3e** ($CDCl_3$, 125.77 MHz)

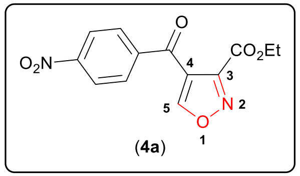

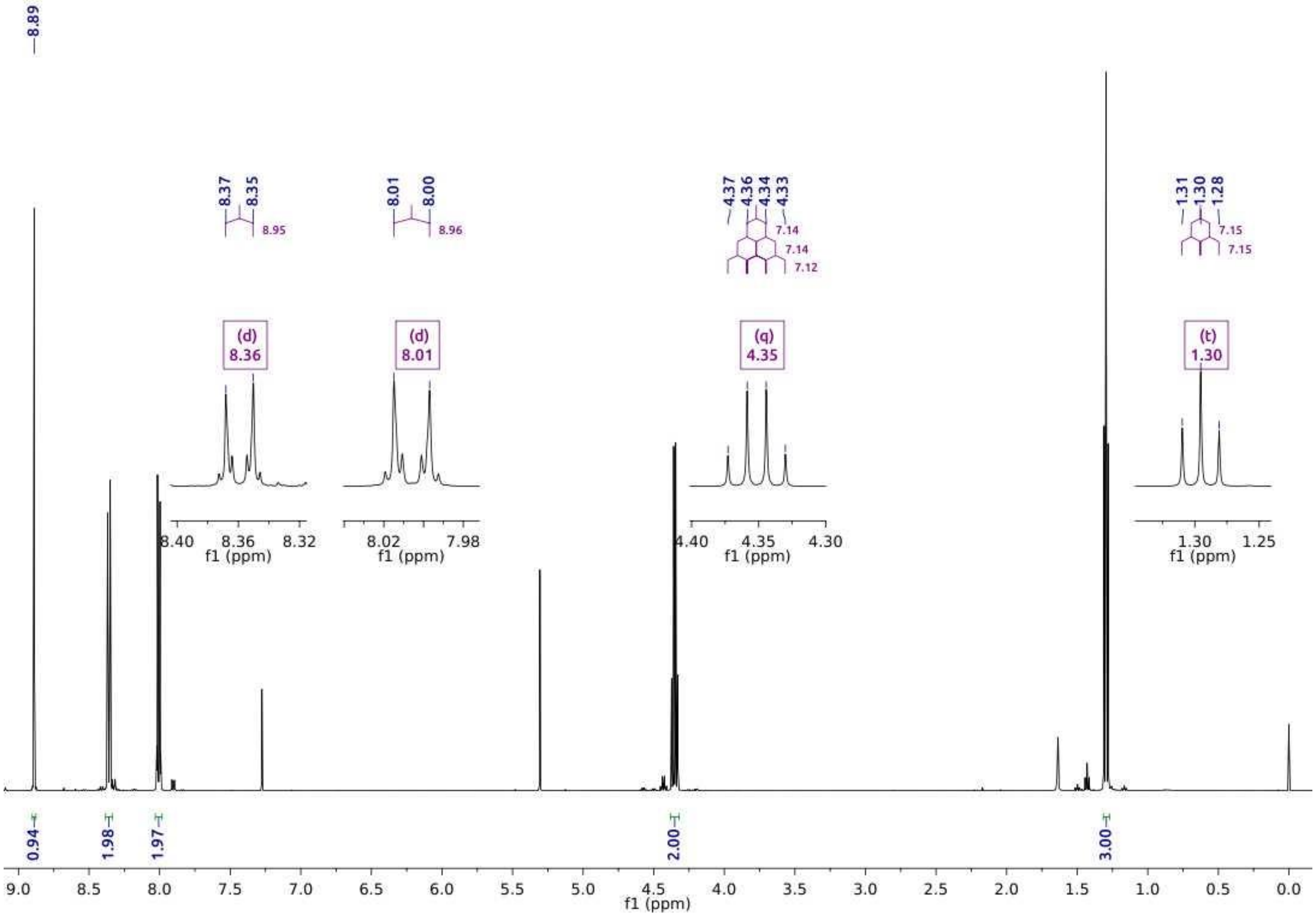

Figure SI-25. ^{1}H NMR spectrum of **4a** ($CDCl_3$, 500.13 MHz)

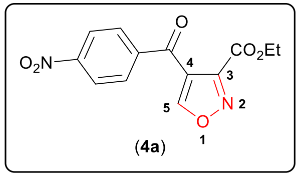

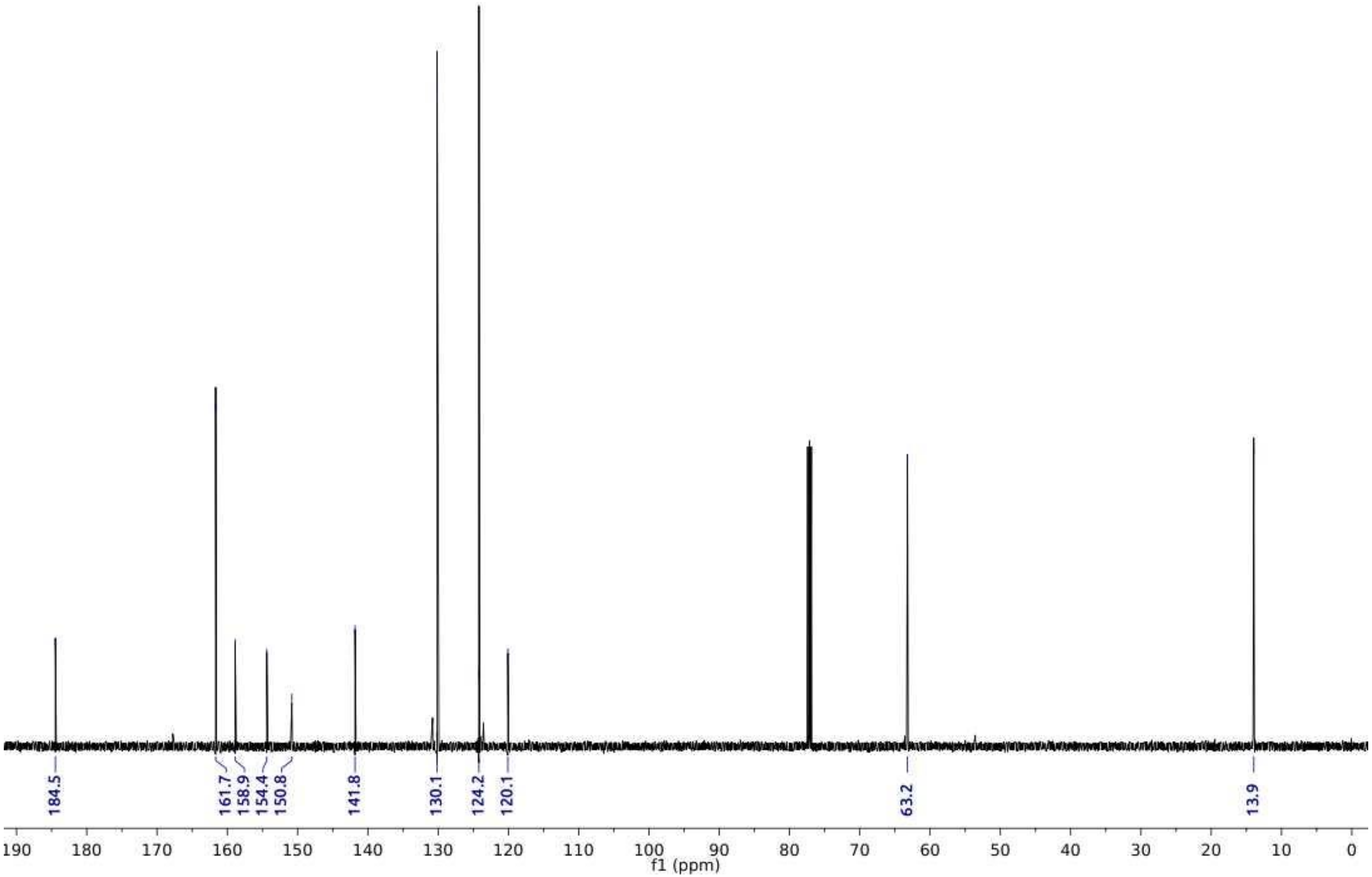

Figure SI-26. ^{13}C NMR spectrum of **4a** ($CDCl_3$, 125.77 MHz)

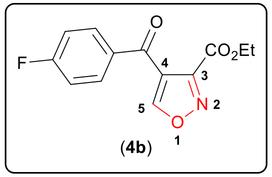

(4b)

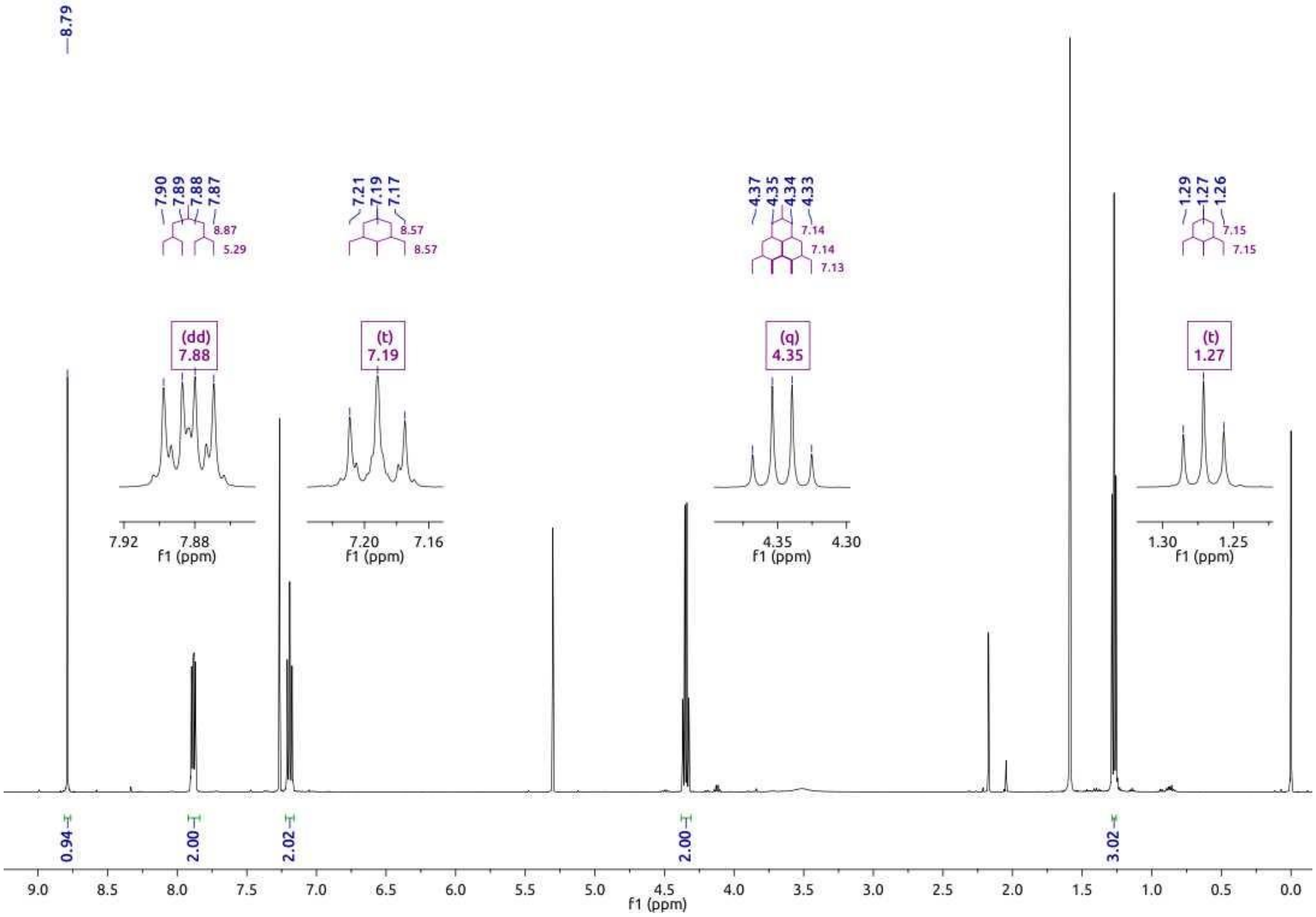

Figure SI-27. ^{1}H NMR spectrum of **4b** ($CDCl_3$, 500.13 MHz)

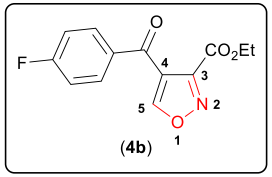

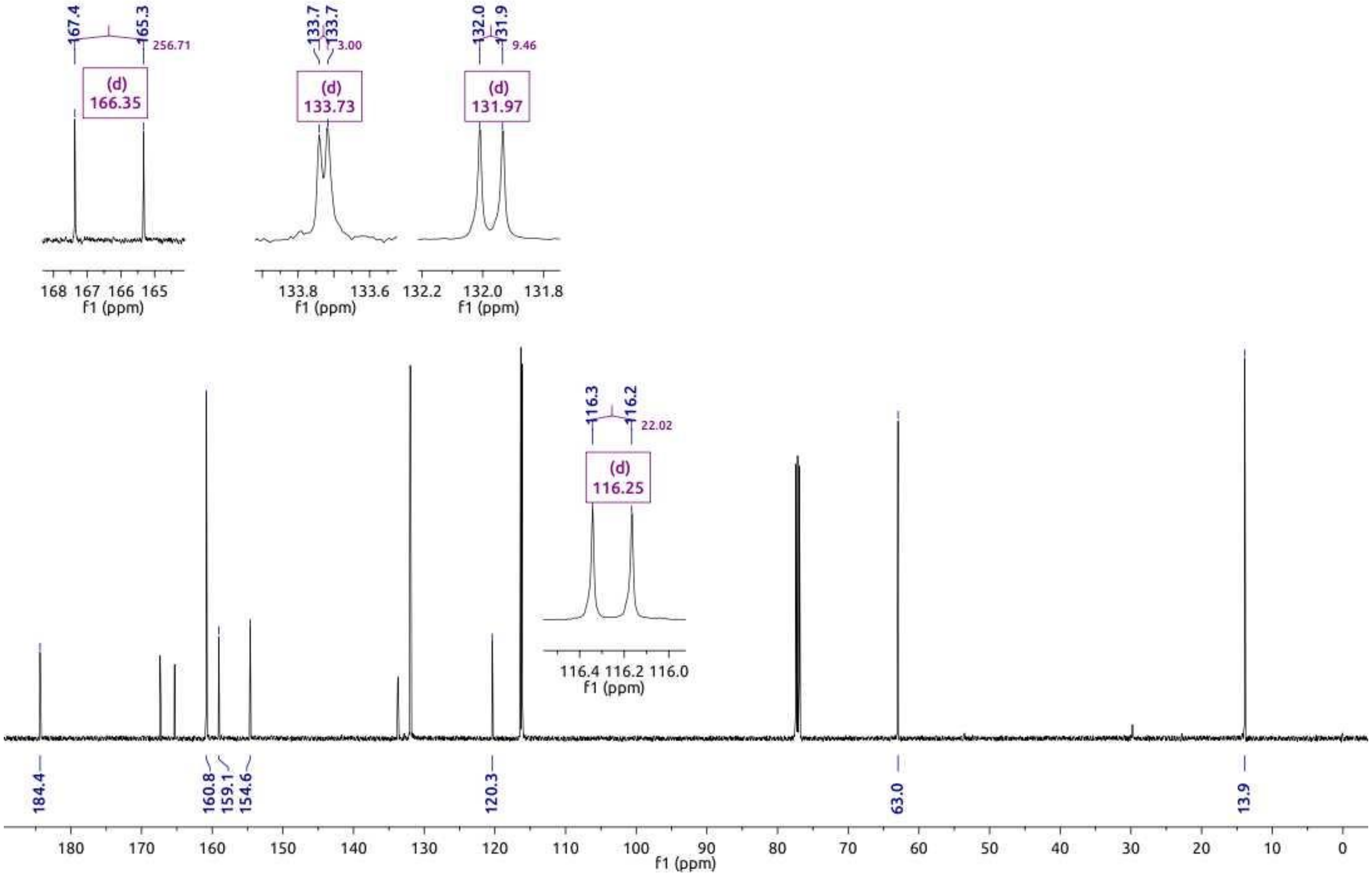

Figure SI-28. ^{13}C NMR spectrum of **4b** ($CDCl_3$, 125.77 MHz)

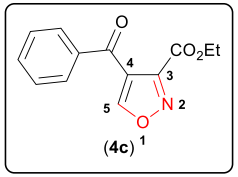

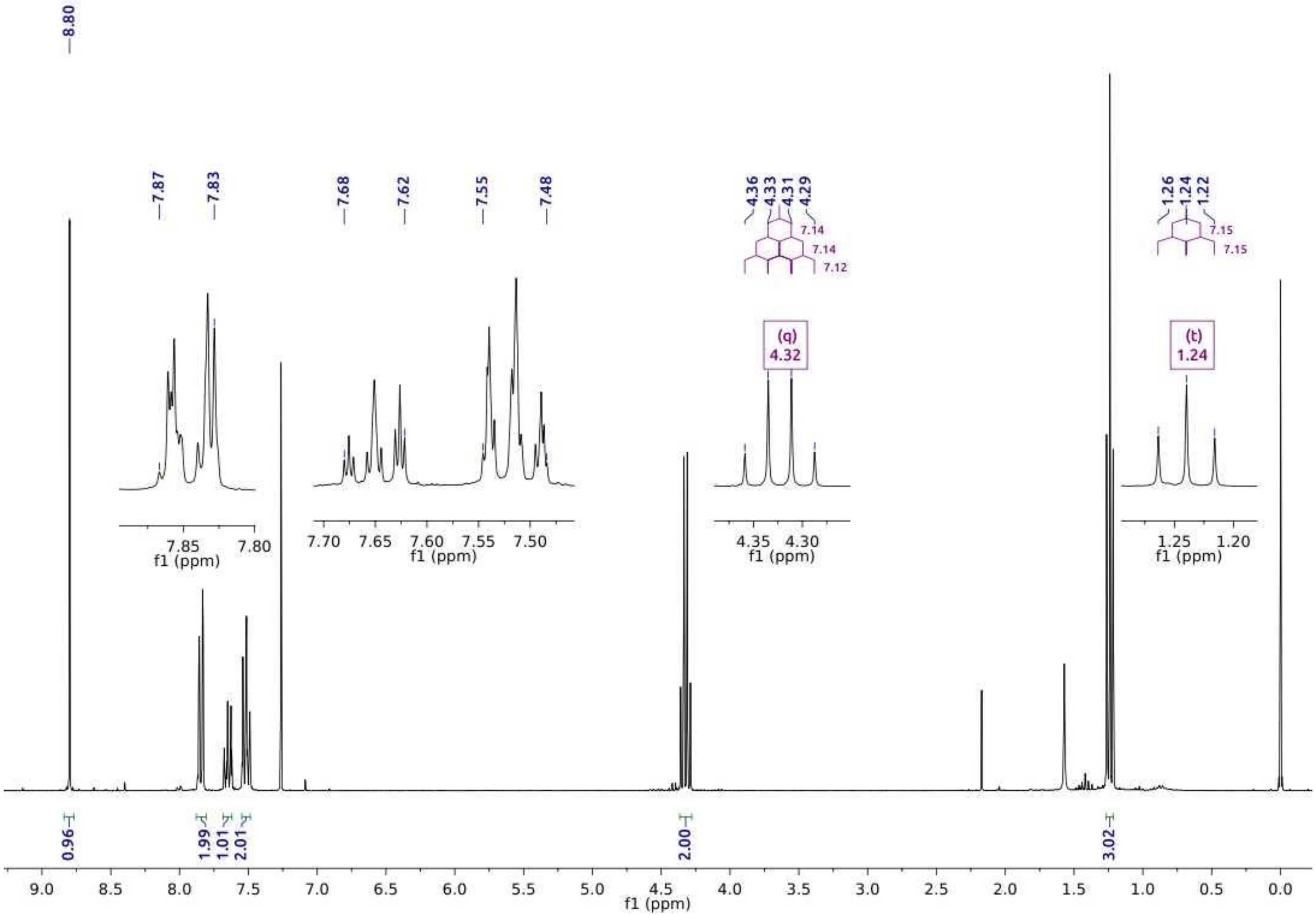

Figure SI-29. ^{1}H NMR spectrum of **4c** ($CDCl_3$, 300.06 MHz)

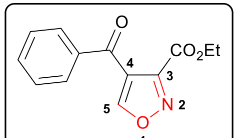

(**4c**)

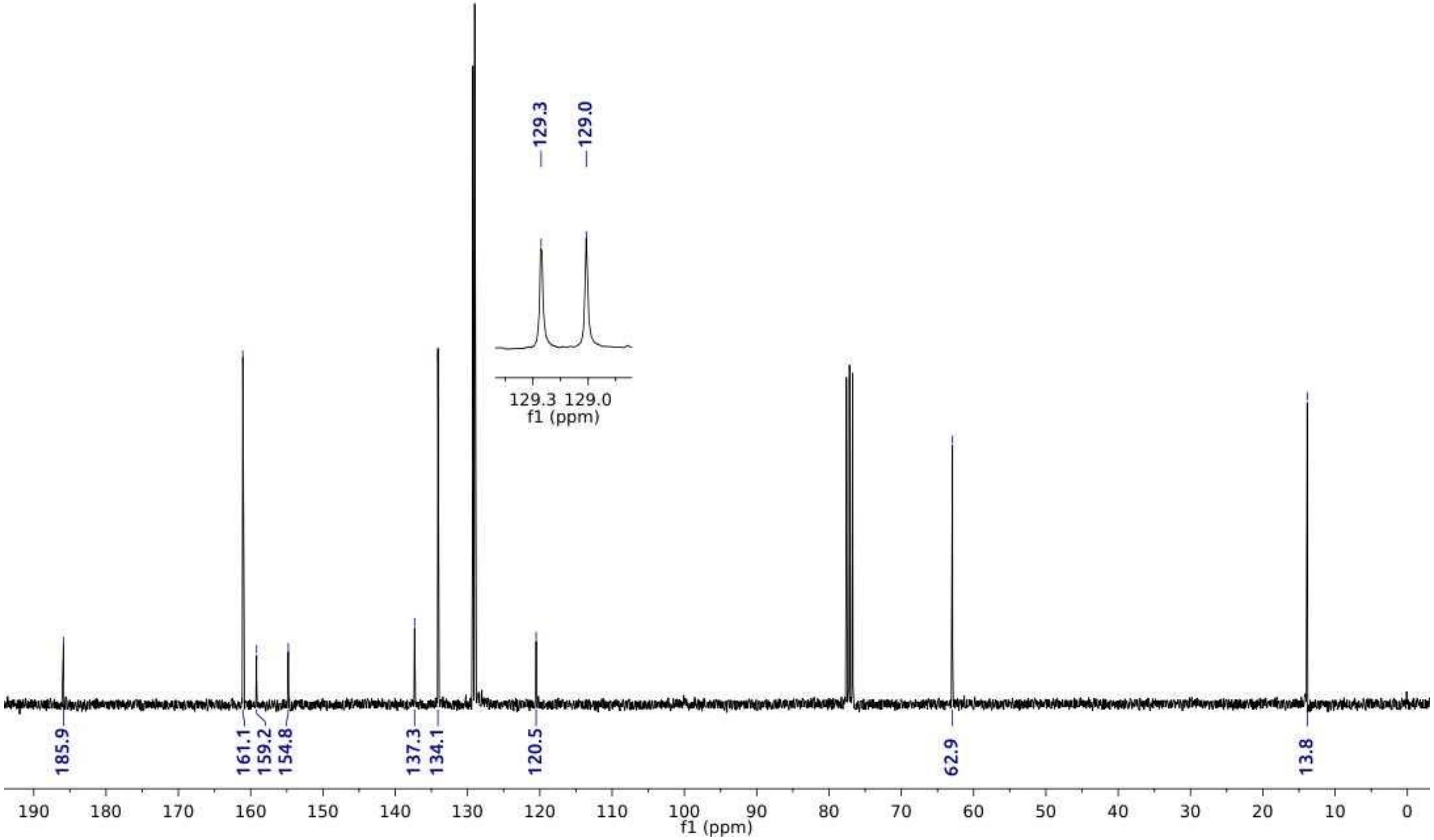

Figure SI-30. ^{13}C NMR spectrum of **4c** ($CDCl_3$, 75.46 MHz)

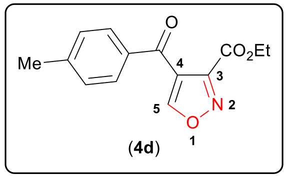

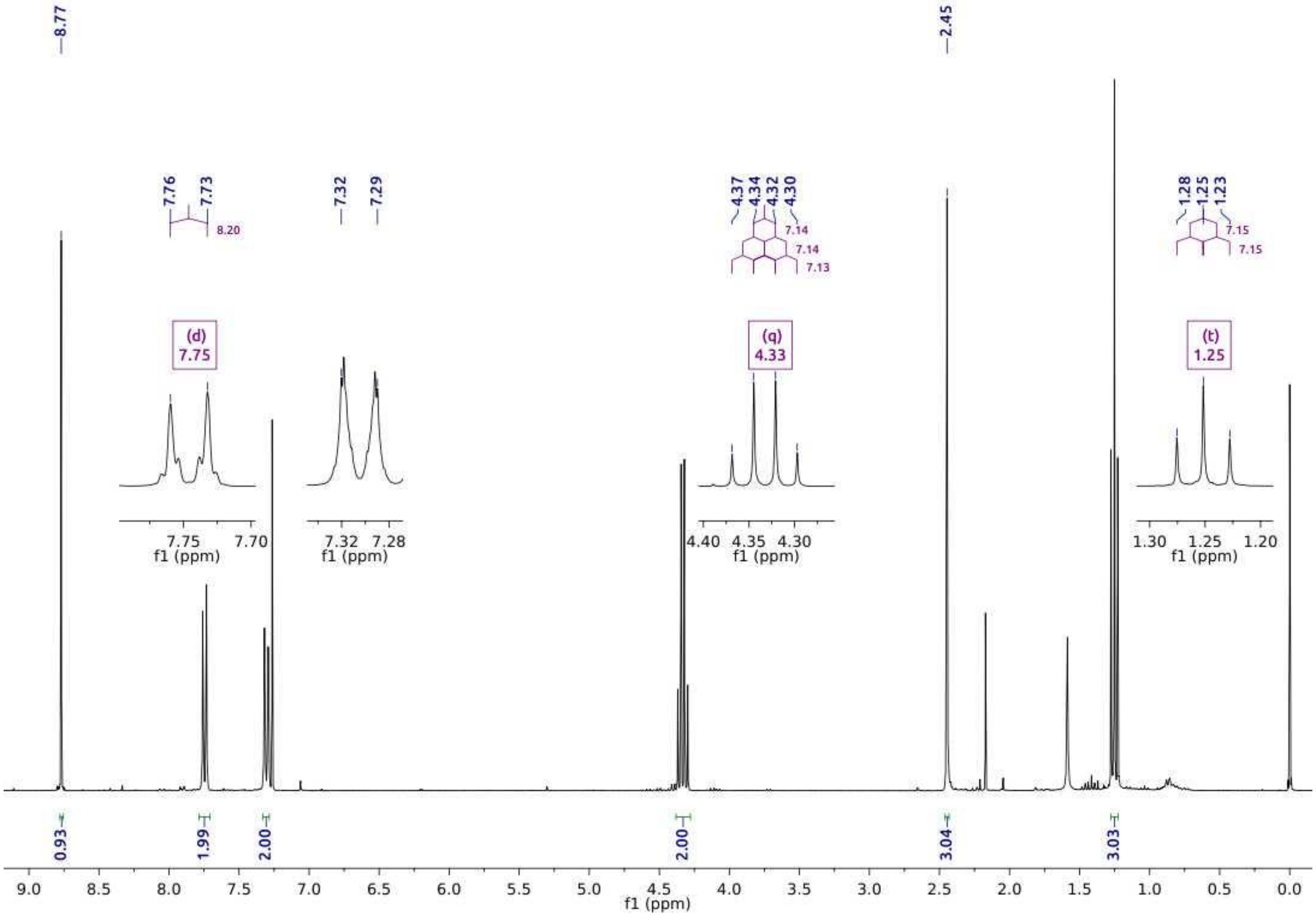

Figure SI-31. ^{1}H NMR spectrum of **4d** ($CDCl_3$, 300.06 MHz)

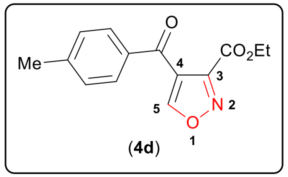

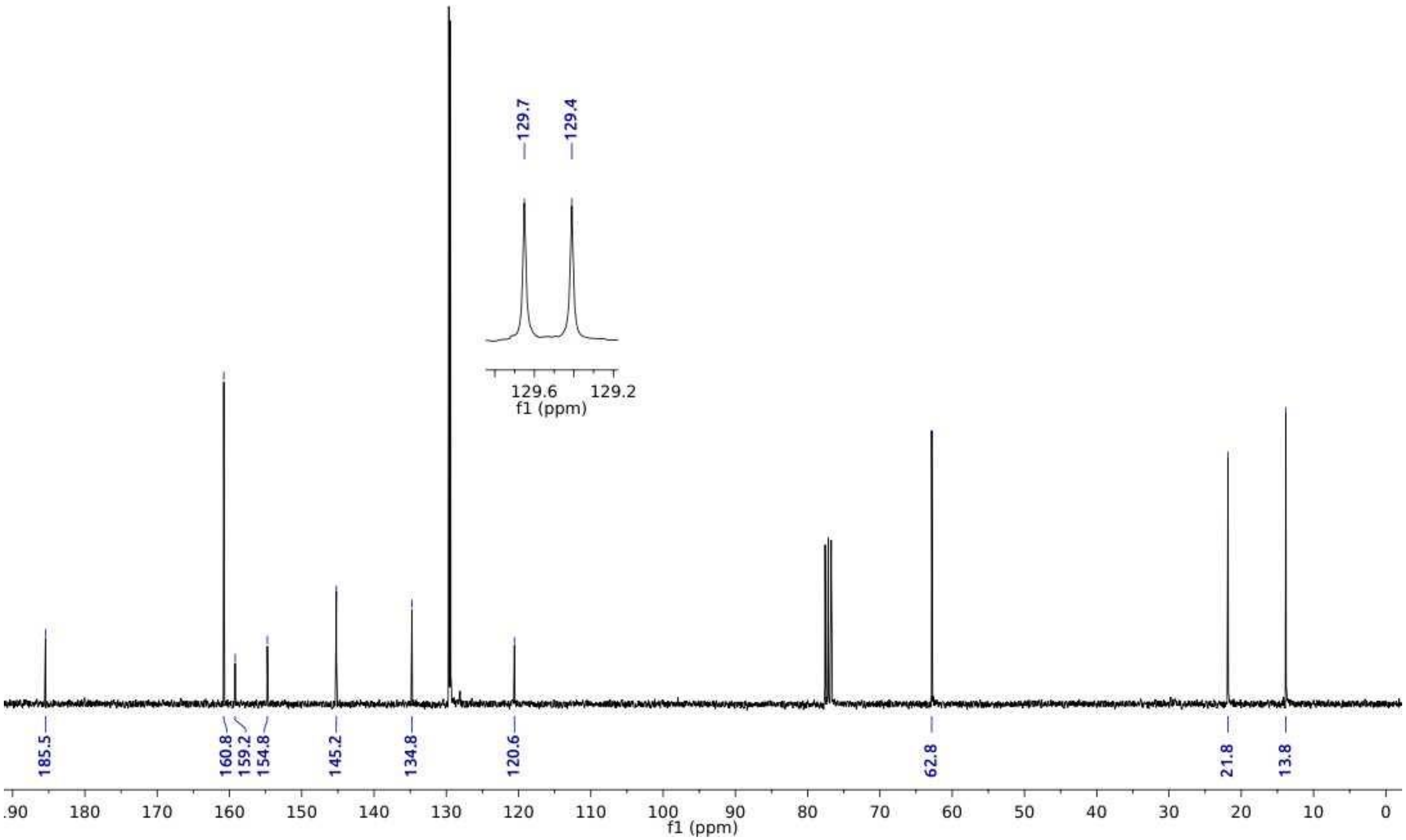

Figure SI-32. ^{13}C NMR spectrum of **4d** ($CDCl_3$, 75.46 MHz)

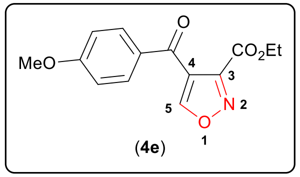

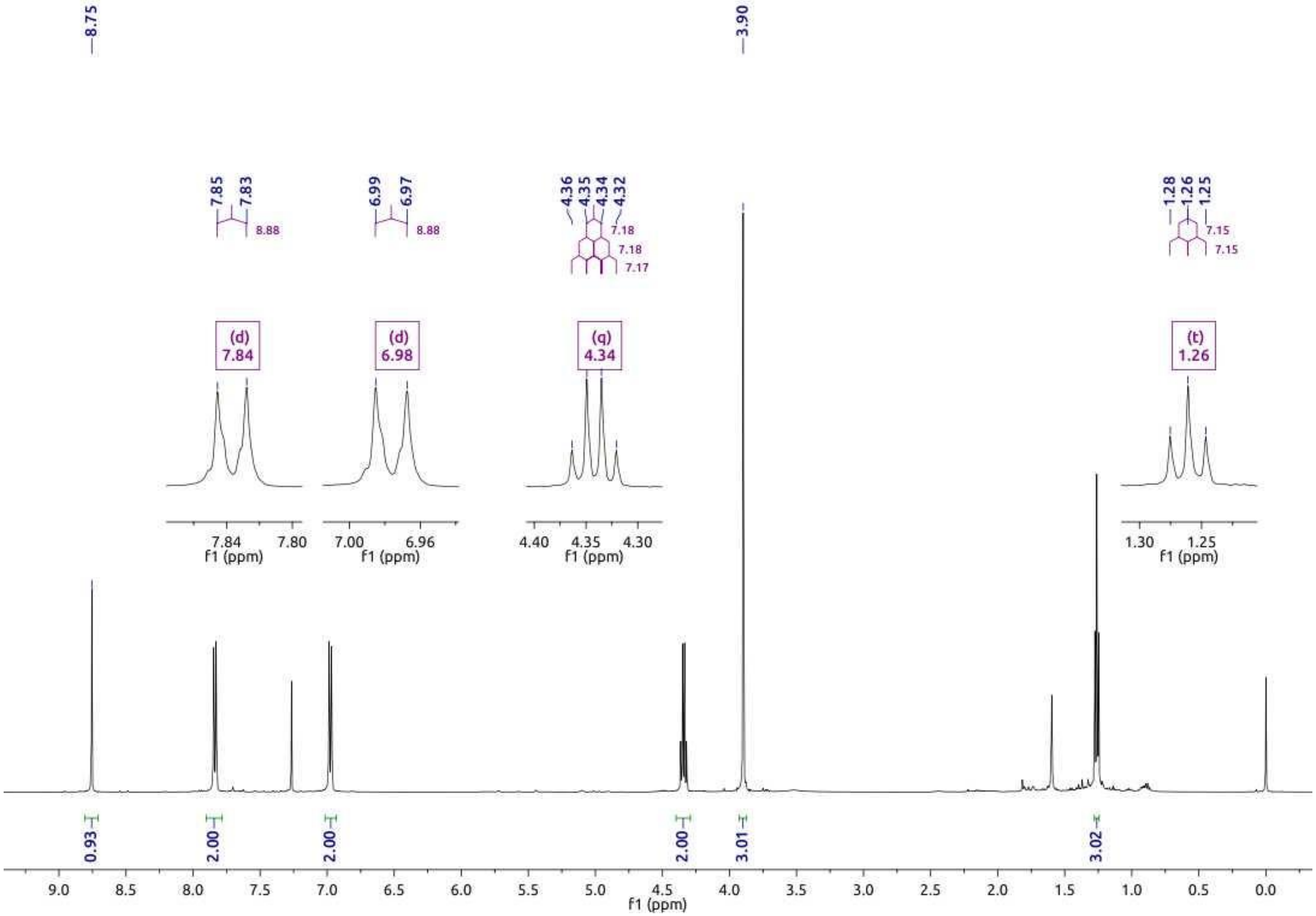

Figure SI-33. ^{1}H NMR spectrum of **4e** ($CDCl_3$, 500.13 MHz)

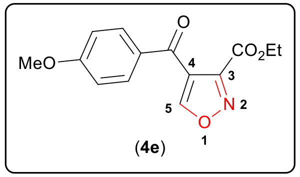

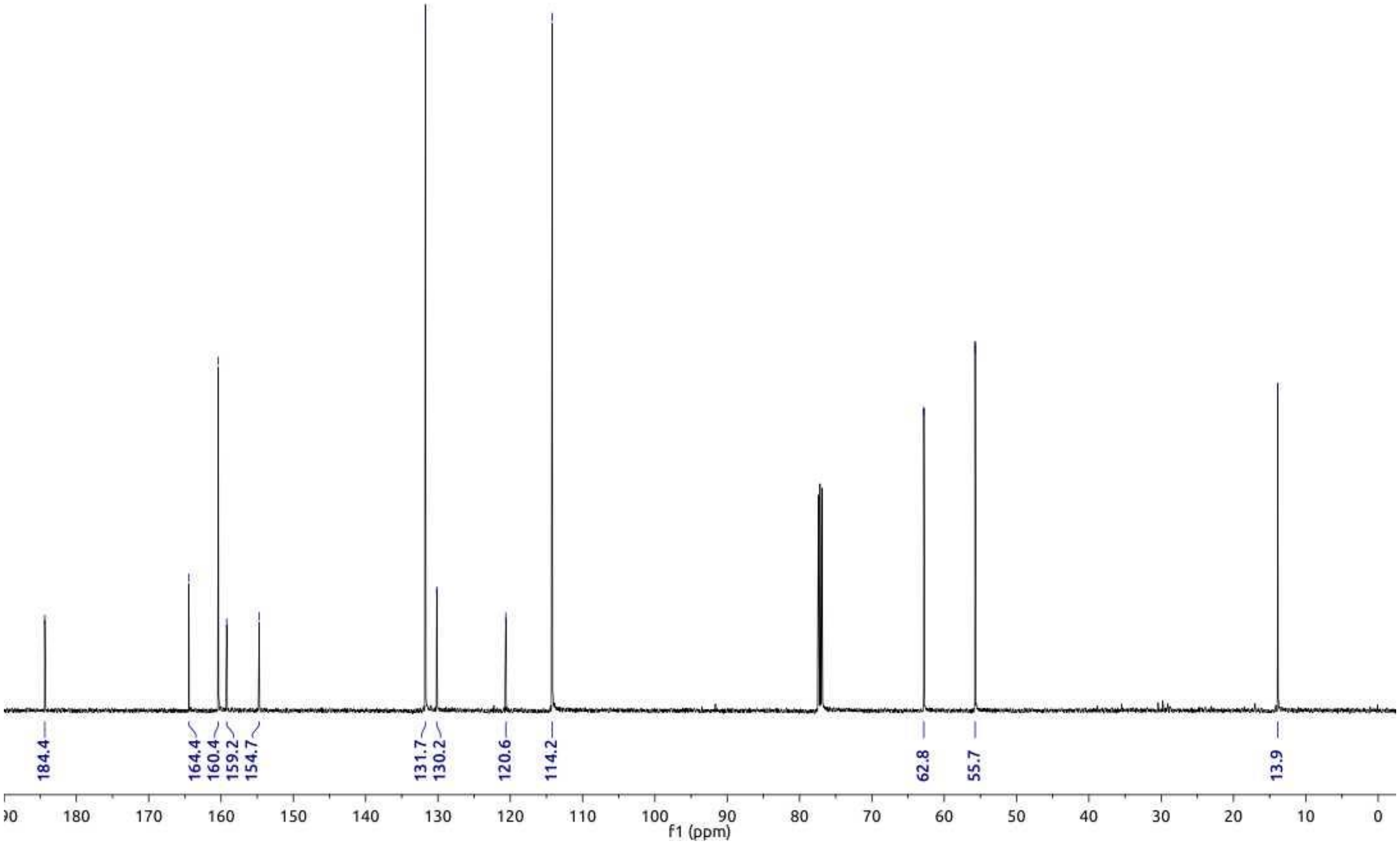

Figure SI-34. ^{13}C NMR spectrum of **4e** ($CDCl_3$, 125.77 MHz)

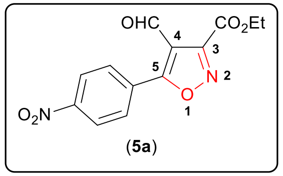

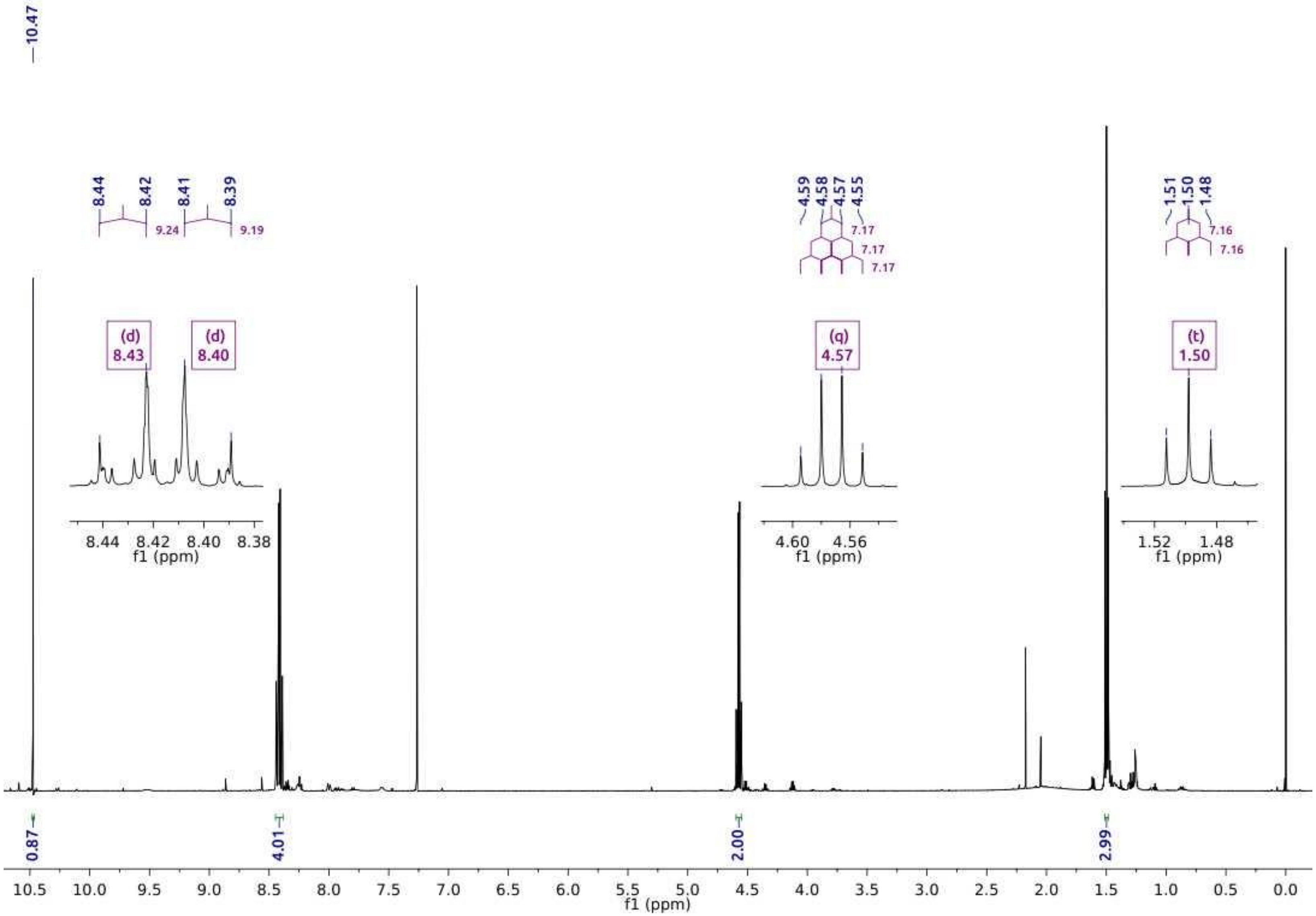

Figure SI-35. ^{1}H NMR spectrum of **5a** ($CDCl_3$, 500.13 MHz)

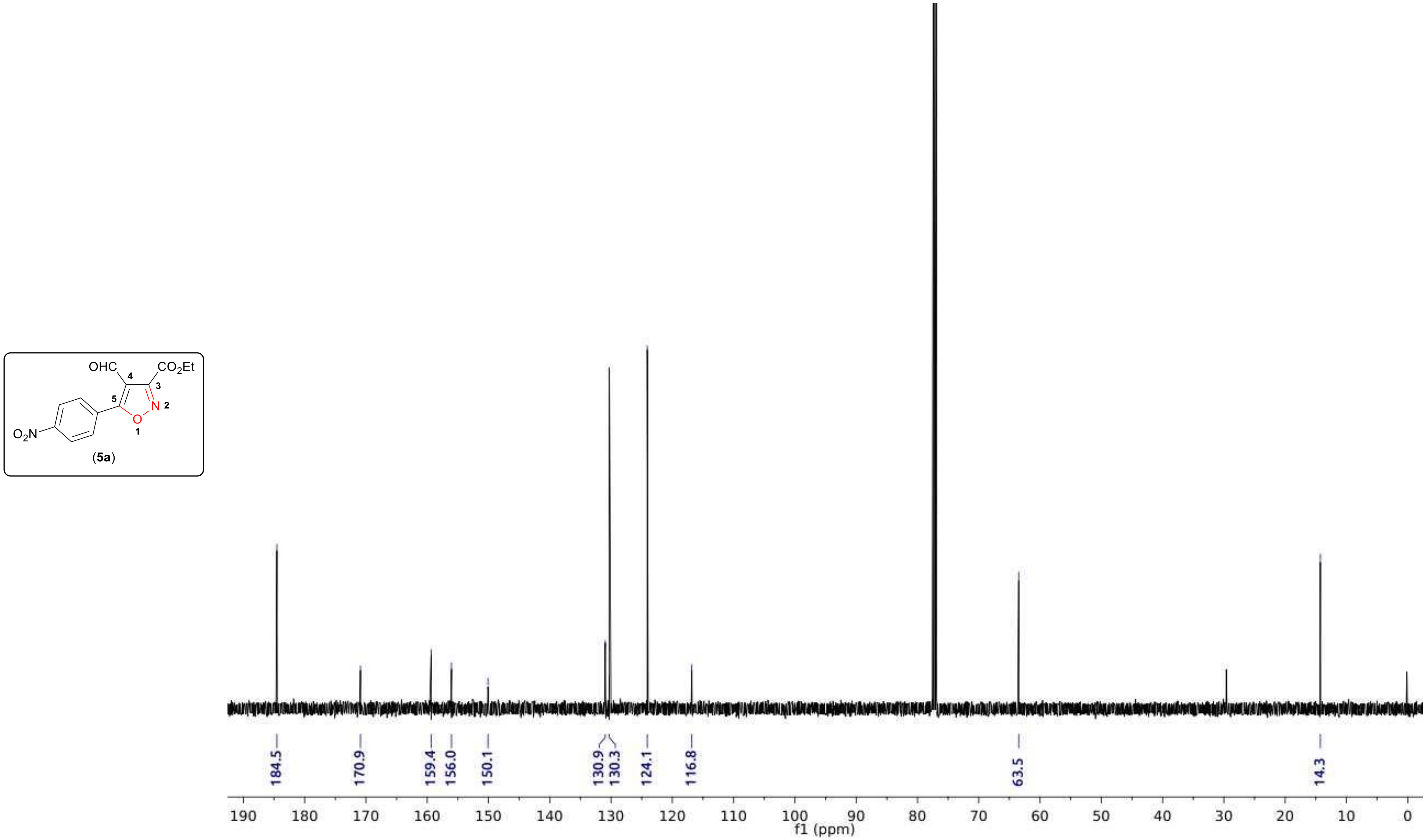

Figure SI-36. ^{13}C NMR spectrum of **5a** ($CDCl_3$, 125.77 MHz)

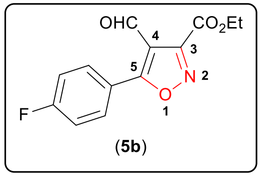

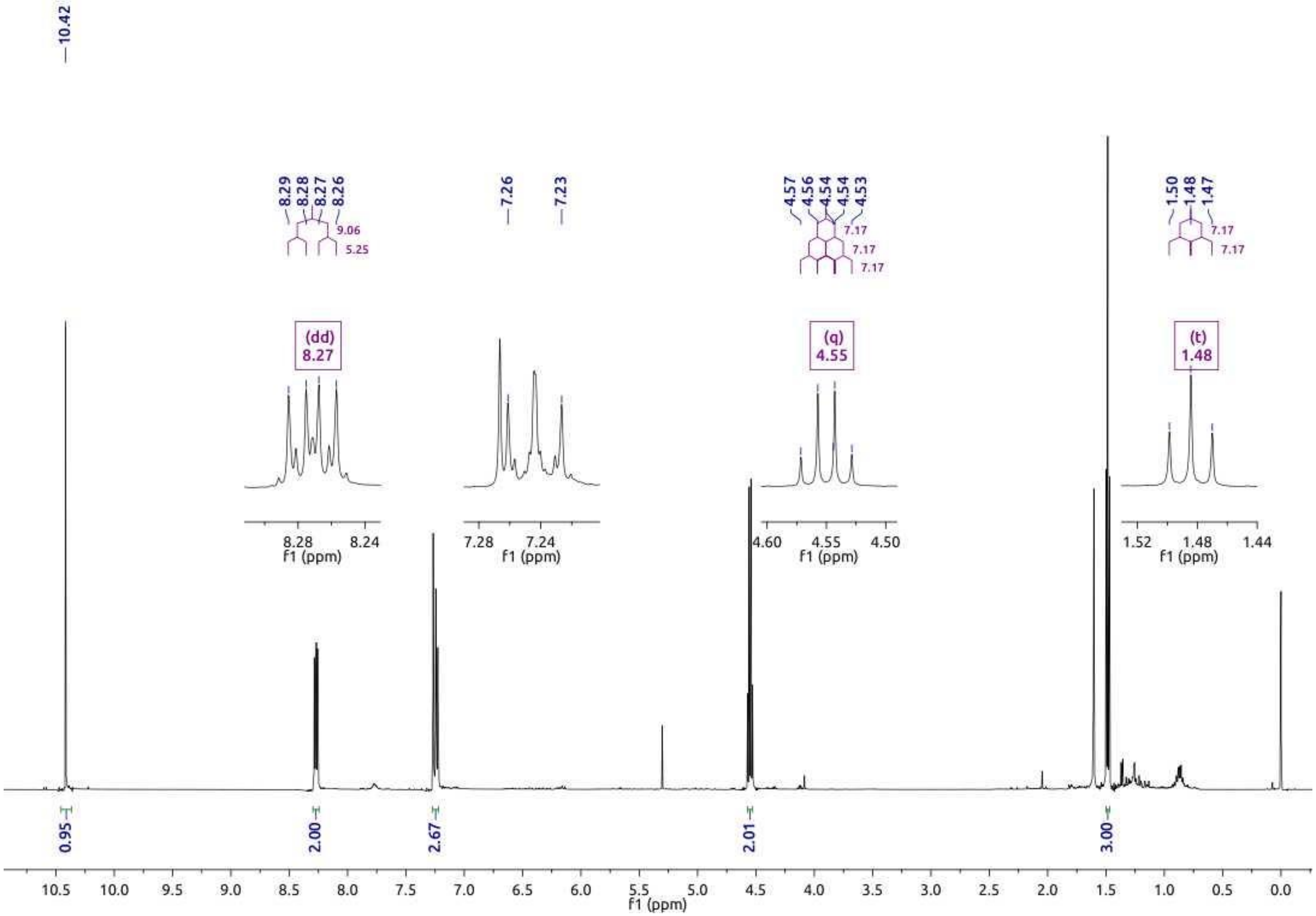

Figure SI-37. ^{1}H NMR spectrum of **5b** ($CDCl_3$, 500.13 MHz)

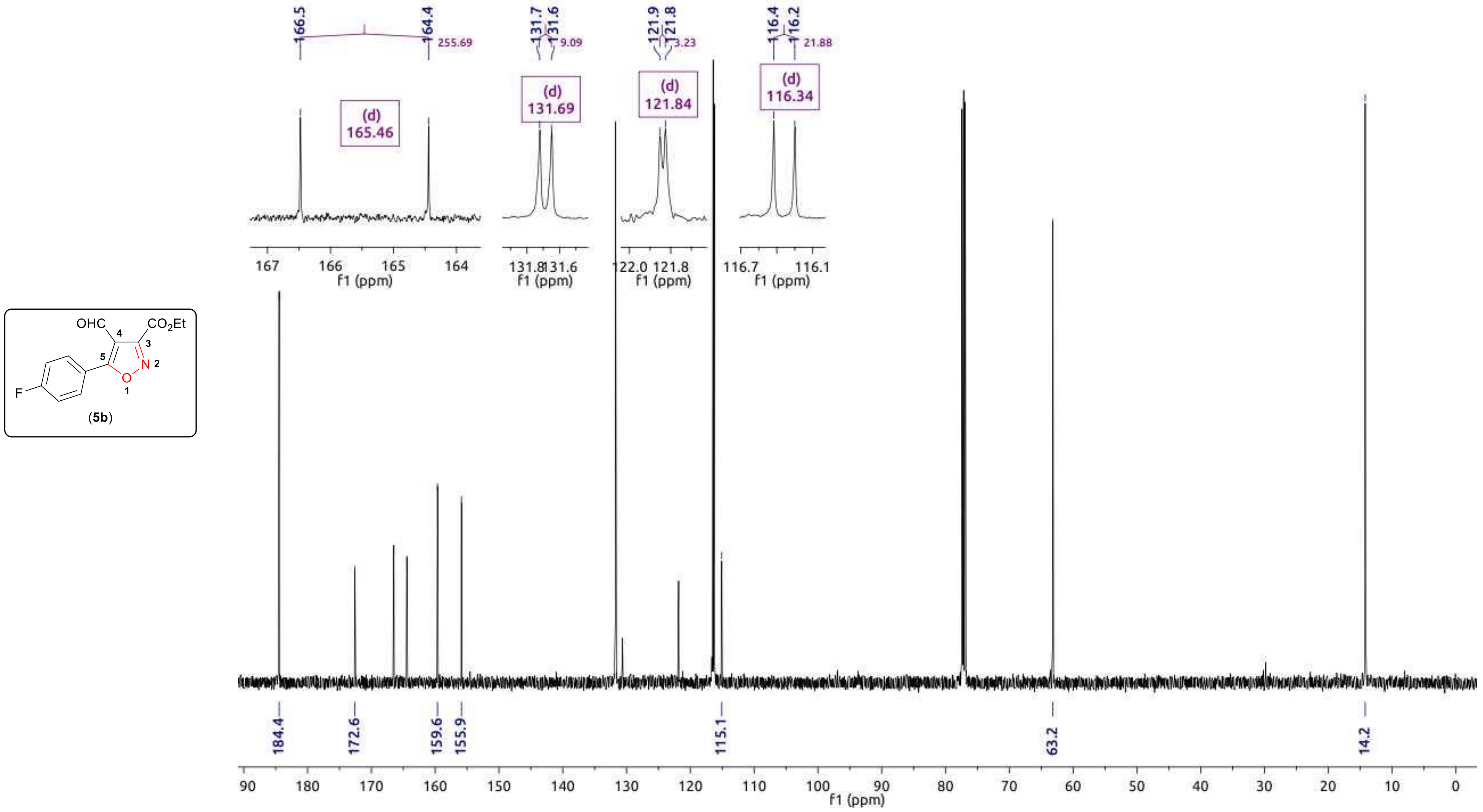

Figure SI-38. ^{13}C NMR spectrum of **5b** ($CDCl_3$, 125.77 MHz)

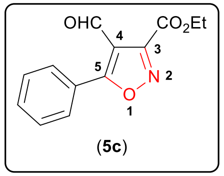

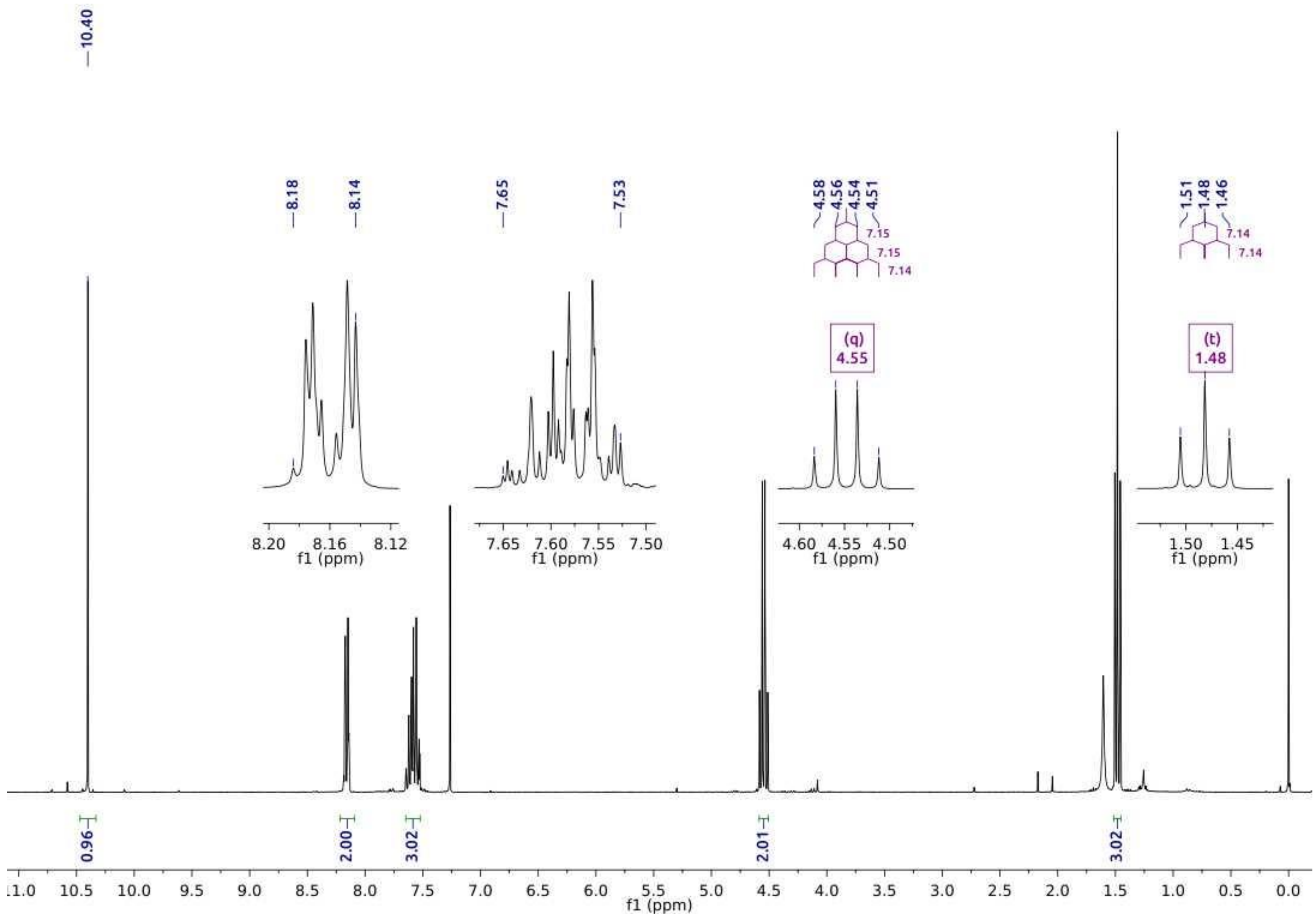

Figure SI-39. ^{1}H NMR spectrum of **5c** ($CDCl_3$, 300.06 MHz)

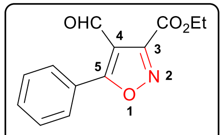

(**5c**)

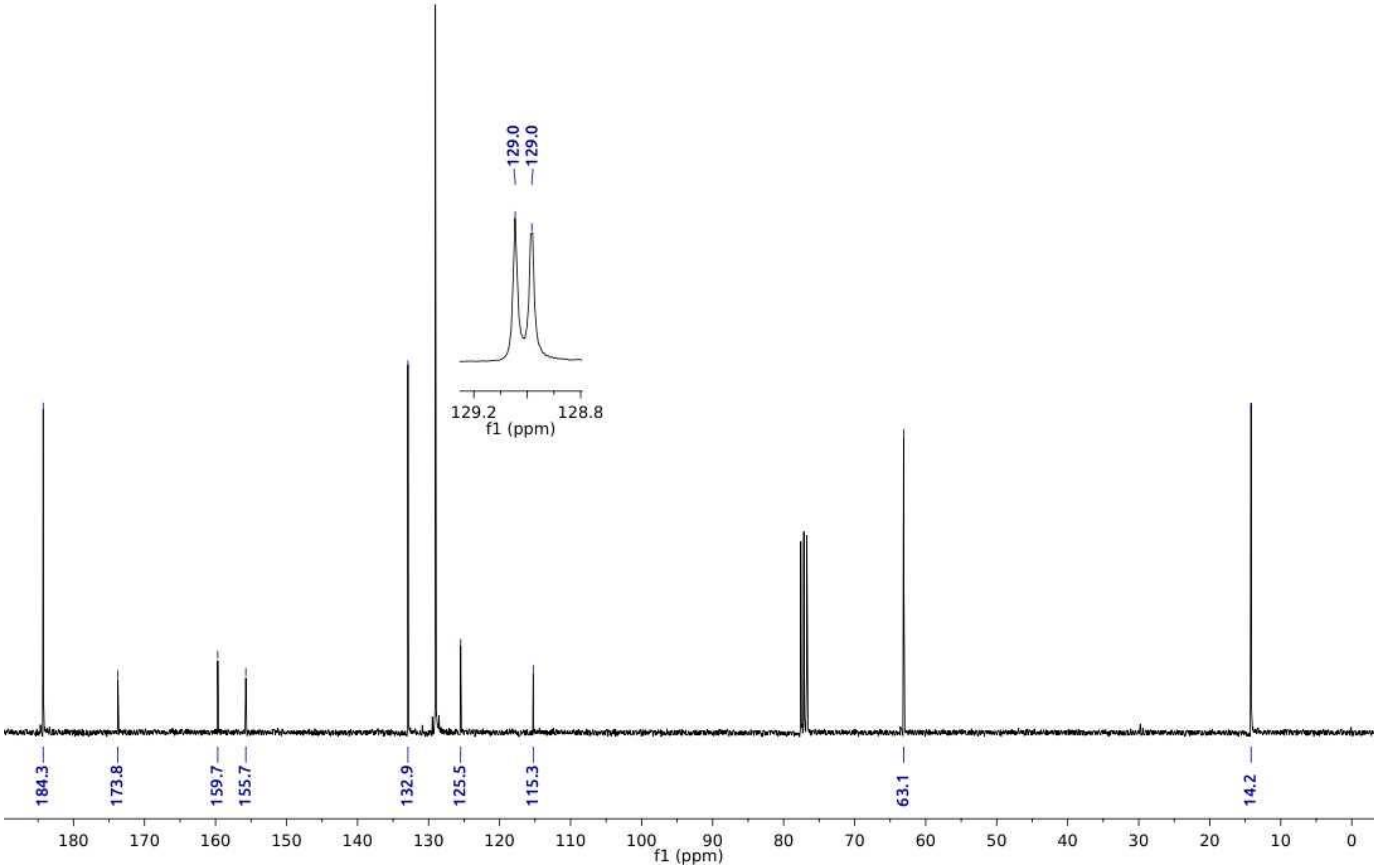

Figure SI-40. ^{13}C NMR spectrum of **5c** ($CDCl_3$, 75.46 MHz)

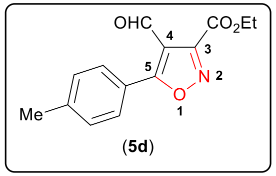

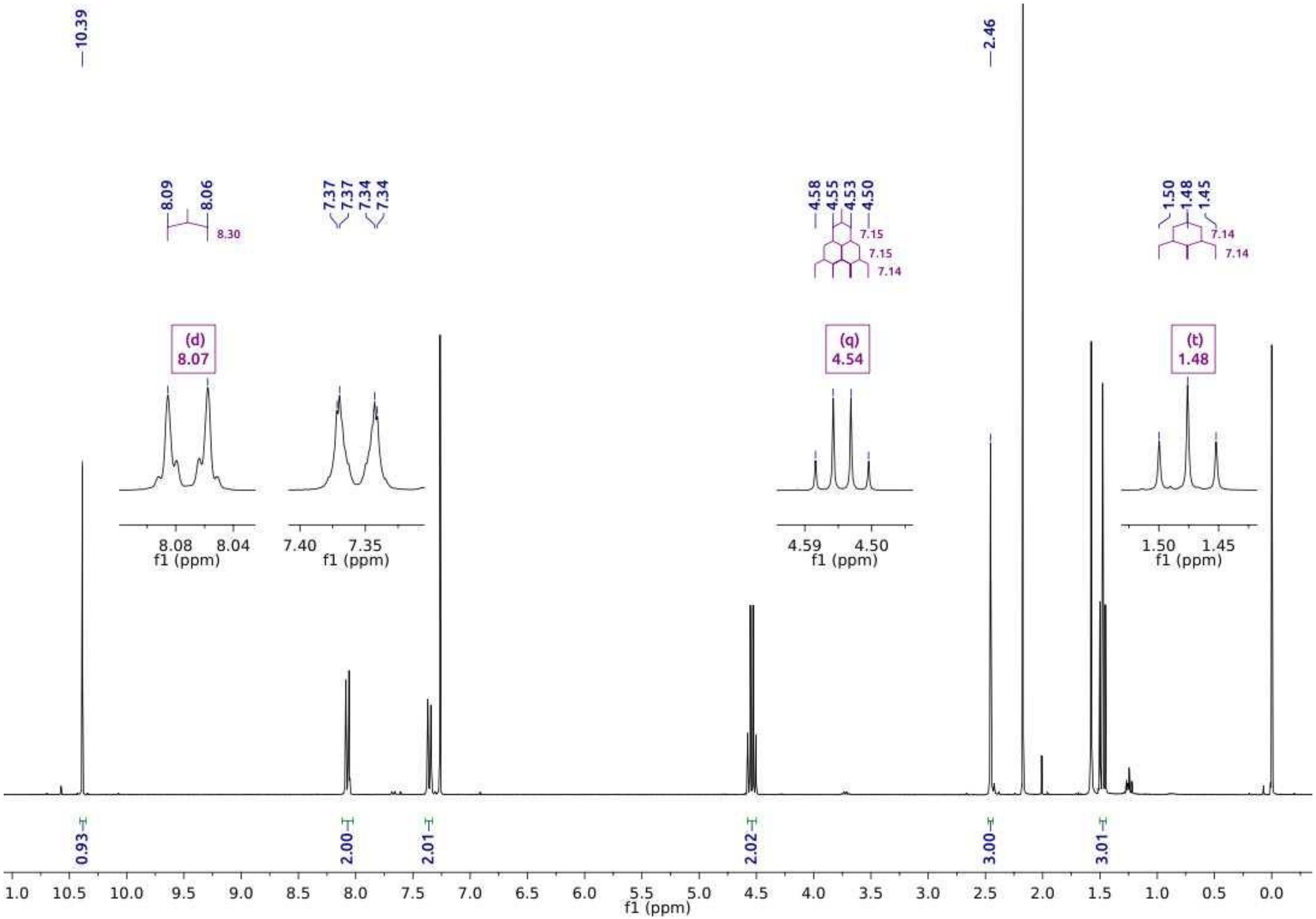

Figure SI-41. ^{1}H NMR spectrum of **5d** ($CDCl_3$, 300.06 MHz)

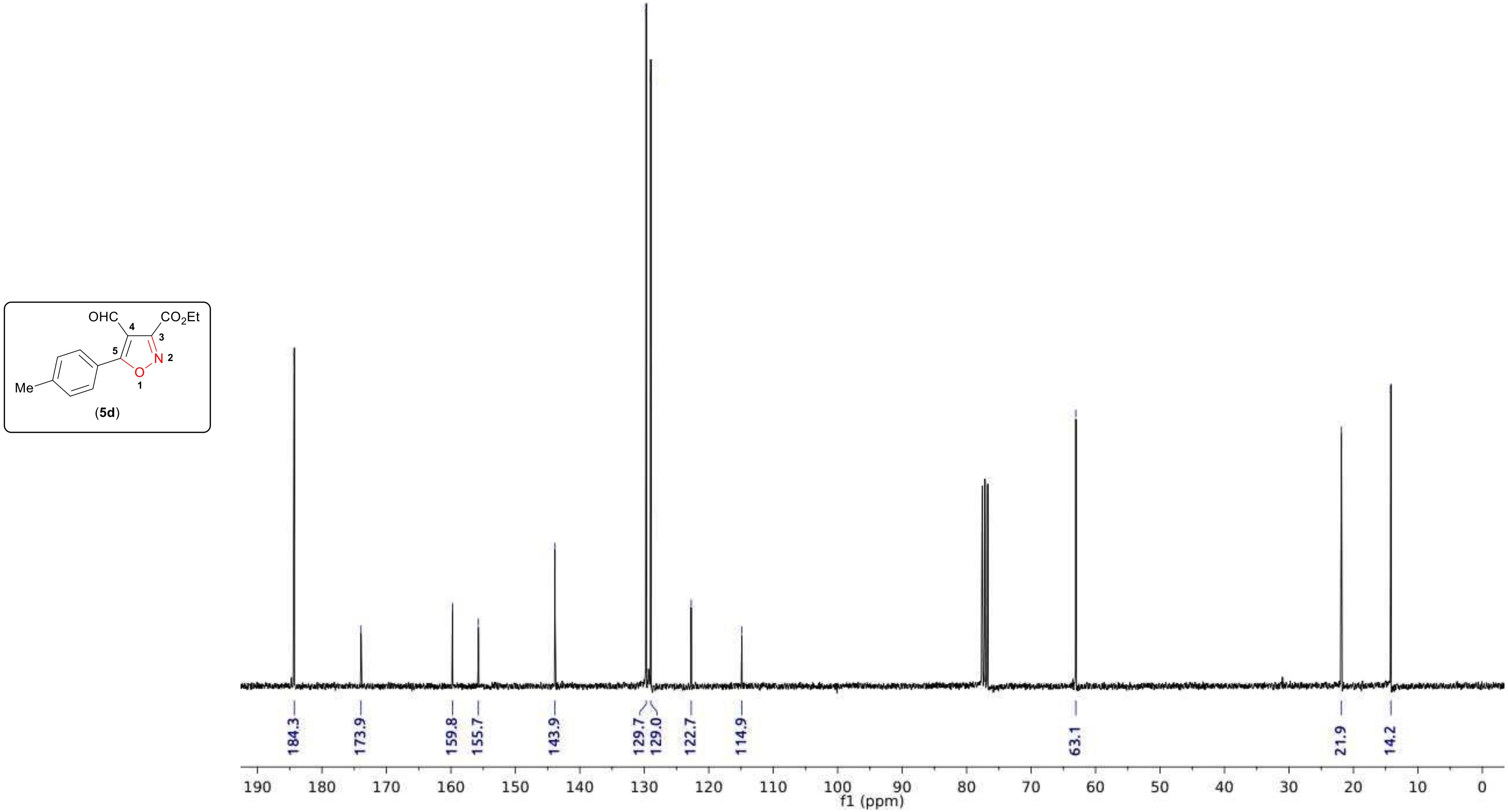

Figure SI-42. ^{13}C NMR spectrum of **5d** ($CDCl_3$, 75.46 MHz)

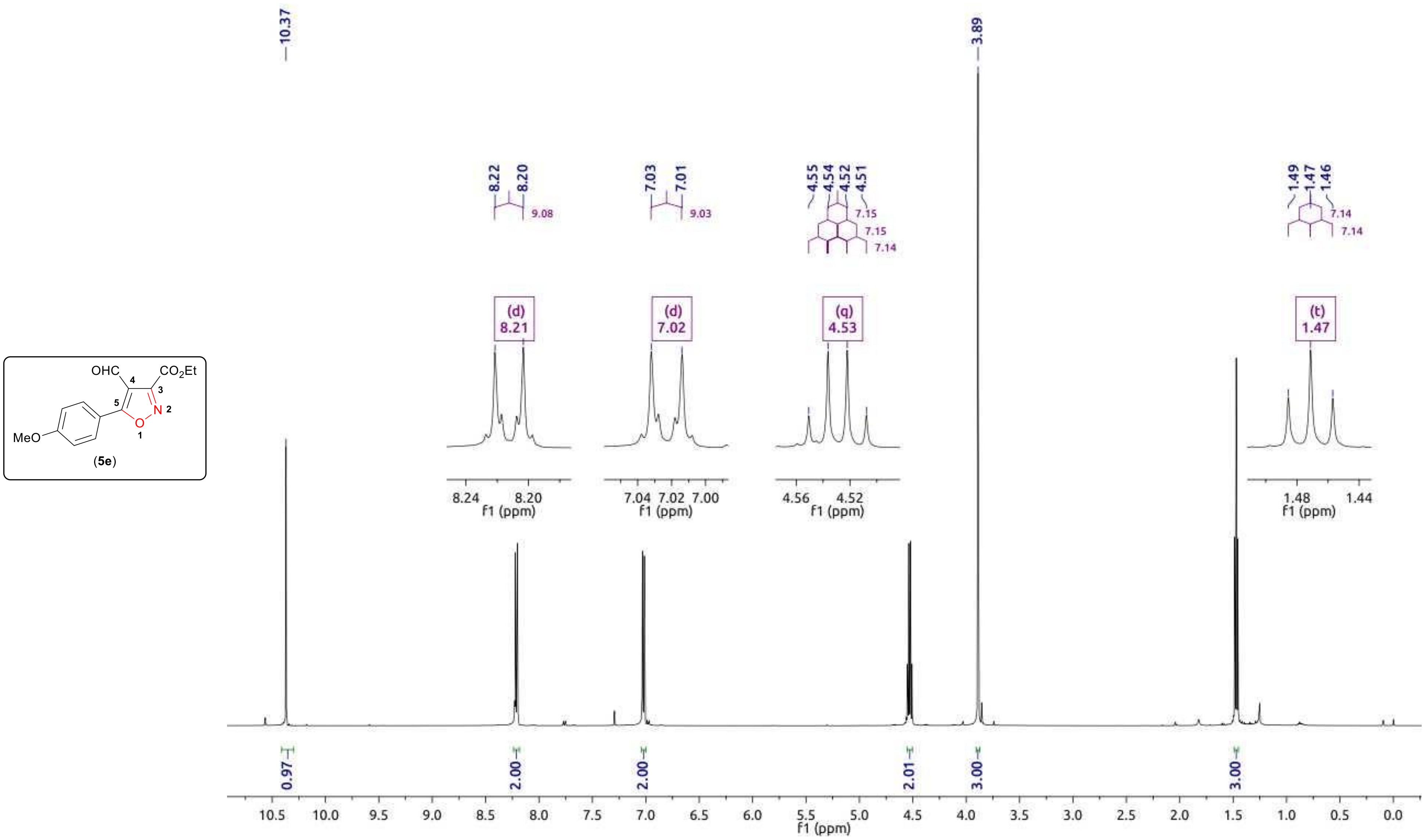

Figure SI-43. ^{1}H NMR spectrum of **5e** ($CDCl_3$, 500.13 MHz)

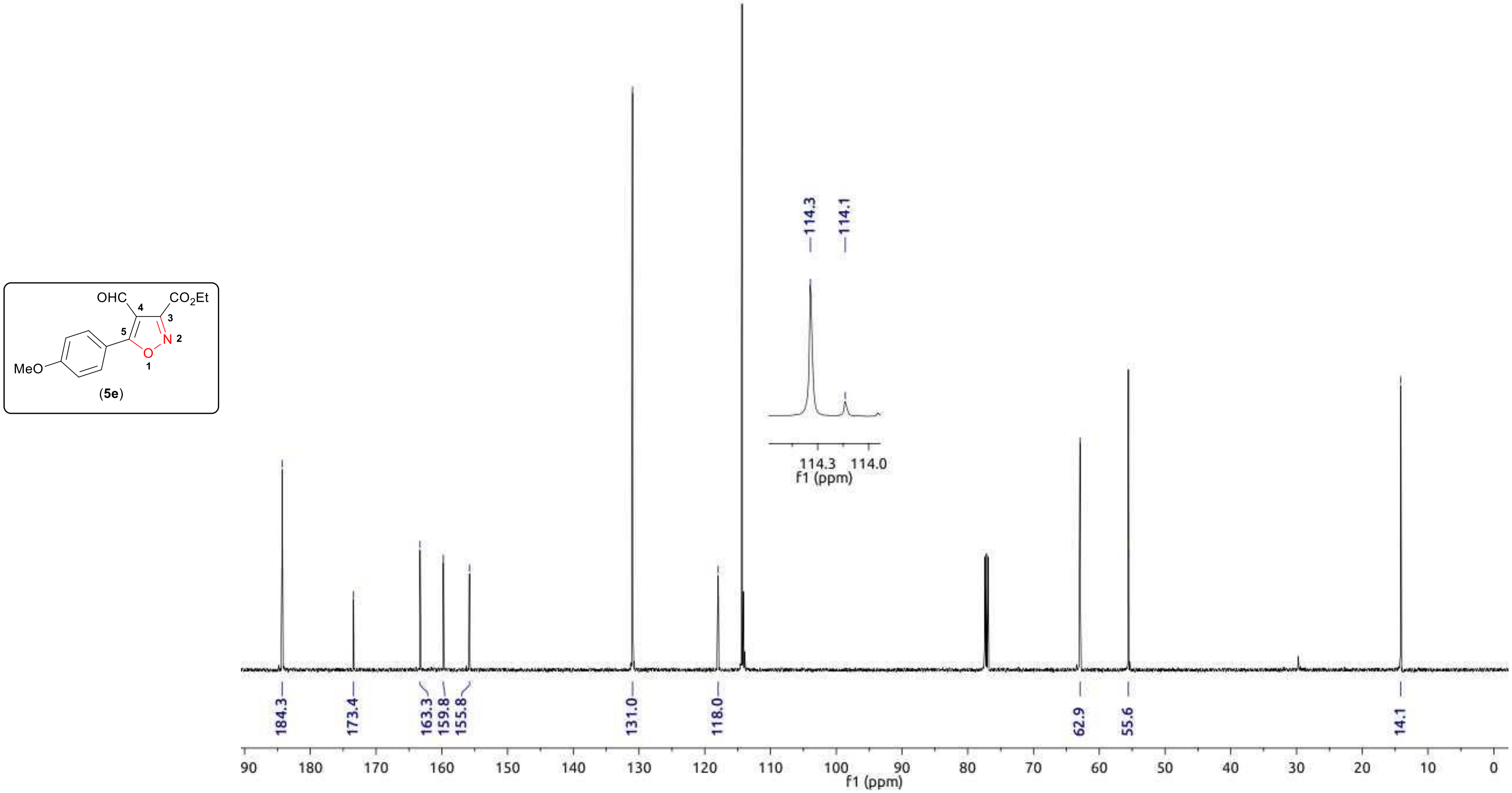

Figure SI-44. ^{13}C NMR spectrum of **5e** ($CDCl_3$, 125.77 MHz)
